# Backbone Thioamidation of a Ribosomal Subunit Protein in Pseudomonadota

**DOI:** 10.1021/acs.biochem.5c00829

**Published:** 2026-03-12

**Authors:** Andrew J. Rice, Yanqing Xue, Andi Liu, Sangeetha Ramesh, Oyin A. Ashiru, Salimat O. Sofela, Douglas A. Mitchell

**Affiliations:** † Department of Biochemistry, Vanderbilt University School of Medicine—Basic Sciences, Nashville, Tennessee 37232, United States; ‡ Department of Microbiology, 14589 University of Illinois at Urbana−Champaign, Urbana, Illinois 61801, United States; § Department of Chemistry, Vanderbilt University, Nashville, Tennessee 37232, United States

## Abstract

The ribosome is an intensively studied machine responsible for protein synthesis. Recent high-resolution structures of the *Escherichia coli* ribosome unexpectedly revealed a thioamide on the large subunit protein uL16. This unusual and easily overlooked modification replaces oxygen with sulfur in the peptide backbone, and here, the modification is proximal to the peptidyl transferase center (PTC). The responsible enzyme has remained unidentified, although methanogenic YcaO enzymes are known to catalyze thioamidation of methyl-coenzyme M reductase. Here, we use several approaches to assign *Ec*YcaO as the enzyme responsible for uL16 thioamidation. We began by individually predicting the structures of all *E. coli* proteins complexed with *Ec*YcaO, revealing that *Ec*uL16 was the only protein forming a high-confidence, catalytically competent interaction. Furthermore, we performed mutational analysis of the *Ec*YcaO-*Ec*uL16 binding interface, revealing an extensive, electrostatically complementary surface atypical of characterized YcaO enzymes. In log-phase *E. coli*, we observed a complex, nonlinear growth relationship between thioamidation and β-hydroxylation of *Ec*uL16-Arg81, a neighboring PTC modification. Beyond *E. coli*, bioinformatics surveys predict that several thousand Pseudomonadota organisms will equivalently perform uL16 thioamidation. This prediction was validated for two Gram-negative human pathogens, *Klebsiella pneumoniae* and *Pseudomonas aeruginosa*. Overall, this work has elucidated the enzyme responsible for uL16 thioamidation and demonstrated that this unusual modification is widespread in Pseudomonadota. Further, we have laid a critical foundation for understanding both the mechanism by which this modification is formed and its functional consequences. The *in silico* approach leveraged here could also find broader use in identifying gene-encoded substrates for enzymes.

## Graphical abstract

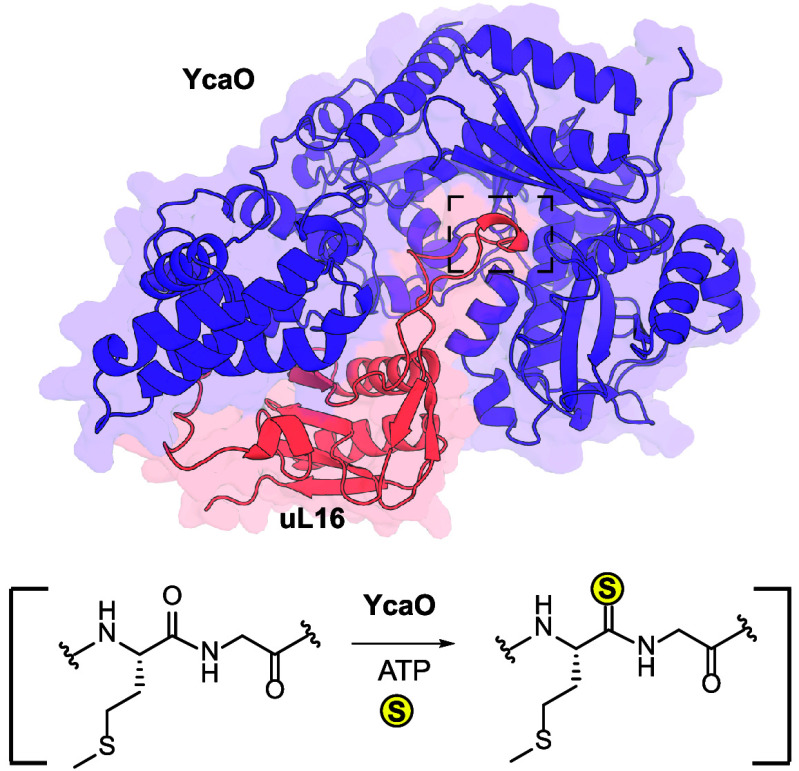

## Introduction

Ubiquitous across all life, the ribosome is a ribonucleoprotein complex essential for protein synthesis. The indispensable function of the ribosome has led to intense evolutionary optimization, producing large and small subunit proteins whose amino acid sequences are highly conserved, with only modest variation across organisms to match their ecological niches.^1^ Despite this conservation, ribosomes retain remarkable plasticity, dynamically adjusting their activity in response to intracellular and extracellular cues. Ribosome function is further tuned through interactions with regulatory proteins, RNAs, or small molecules, exemplified by processes such as ribosomal hibernation.^2,3^

Post-translational modifications can further influence ribosomal function.^4–6^ Certain modifications fine-tune translation, such as methylation of uL3-His245 in eukaryotes, which modulates elongation by inducing pausing events at specific codons.^7,8^ Other modifications can broadly alter ribosomal activity, such as phosphorylation of uL10.^9^ Post-translational modification of ribosomal proteins can also be detrimental when dysregulated. For instance, excess phosphorylation of uS19 by pathogenic LRRK2 variants drives global increases in translation and have been linked to Parkinson’s disease.^10^ Further, trimethylation of Lys22 in eL40 promotes the progression of gastric adenocarcinoma.^11^

Recent advances in structural biology techniques have uncovered previously unrecognized ribosomal modifications. Watson et al. reported a cryogenic electron microscopy (cryo-EM) structure of the *Escherichia coli* ribosome at a global resolution of 2.0 Å.^12^ Analysis of this structure revealed that large subunit protein uL16 (UniProt: P0ADY7) (Table S1) contained a backbone thioamide at residue Met82, replacing the canonical amide bond. Remarkably, this modification had escaped detection despite decades of study and the fact that an adjacent residue, Arg81, undergoes modification to *(3R)*-3-hydroxyarginine (Figure 1).^13,14^

**Figure 1:**
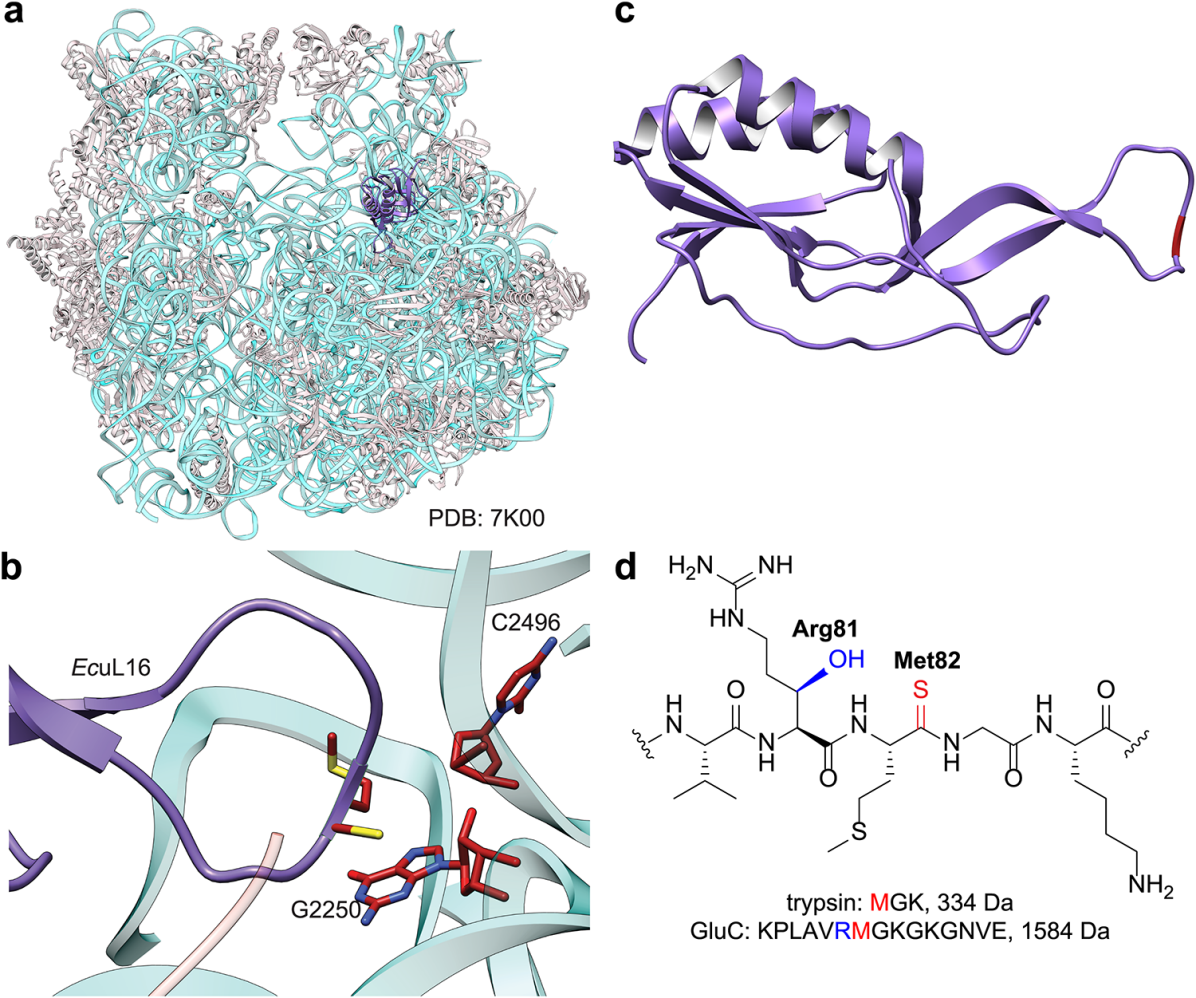
(a) Cryo-EM structure of the *E. coli* ribosome (PDB: 7K00). rRNA is cyan and proteins are gray. *Ec*uL16 is highlighted in purple. (b) The thioamide of *Ec*uL16 (Met82), shown in proximity of the peptidyl transferase center. Two nucleotides implicated for translation are labeled. (c) *Ec*uL16, isolated from the cryo-EM structure in panel a. Met82, which is thioamidated, is depicted in fire-brick red. (d) Line-angle drawing of residues 80–84 of *Ec*uL16. Hydroxylation of Arg81 and thioamidation of Met82 are highlighted in blue and red, respectively. The tryptic and GluC fragments of *Ec*uL16 containing thioamidation are displayed below with their molecular weights.

The thioamidation of *Ec*uL16-Met82 likely went unnoticed due to the limitations of standard proteomic workflows.^15–17^ Complete tryptic digestion of *Ec*uL16 produces a short tripeptide (MGK, Figure 1), which has a mass below a typical detection window. Furthermore, thioamidation introduces a mass increase of 15.977 Da (O replaced with S) that is nearly isobaric with the 15.995 Da increase caused by net addition of oxygen. Because Met residues are susceptible to sulfoxide (+O) formation during proteomic analyses (Figure 1), peptides containing this modification would likely be misassigned as oxidation rather than backbone thioamidation.^18^

Thioamides are exceptionally rare in proteins. To our knowledge, the only other characterized example occurs in methyl-coenzyme M reductase (MCR), a central enzyme in the global carbon cycle.^19^ MCR is ubiquitous in methanogenic archaea and is the enzyme responsible for producing the majority of methane on Earth.^20,21^ We previously established that a YcaO enzyme installs a thioamide proximal to the active site of MCR. Strains of*Methanosarcina acetivorans* deficient in MCR thioamidation are considerably less robust, exhibiting temperature-sensitive phenotypes and significant increases in doubling time.^22^

Prior to the current study, the protein(s) responsible for *Ec*uL16 thioamidation had not been determined, although drawing an analogy to the case of MCR would suggest the modification could be catalyzed by a YcaO enzyme.^22^ Additionally, YcaO enzymes are often encoded within ribosomally synthesized and post-translationally modified peptide (RiPP) natural product biosynthetic gene clusters.^23^ Early structural work on a cyanobactin-producing YcaO showed that these enzymes often recognize disparate regions of the substrate peptide to perform diverse chemical reactions.^24^ These enzymes share a conserved mechanism in which the peptide backbone amide carbonyl undergoes nucleophilic attack, followed by ATP-dependent phosphorylation and leaving-group elimination to generate the modified product (Figure S1).^25,26^ Depending on the identity of the nucleophile, YcaO enzymes can install diverse modifications, including thiazolines, (methyl)­oxazolines, imidazolines,^27^ macrolactamidines,^28^ and thioamides.^29^ Additional superfamily members can perform proteolysis^26^ as well as install nucleobase-like modifications to peptide backbones.^30^ Reflecting this versatility, YcaO enzymes participate in the biosynthesis of multiple RiPP classes in addition to those that act on intact proteins.^31^

The YcaO enzyme from *E. coli* (*Ec*YcaO, UniProt: P75838) is annotated as a ribosomal protein S12 methylthiotransferase accessory factor.^32^ Ribosomal protein uS12 is known to be β-methylthiolated on Asp88 by the methylthiotransferase RimO.^33,34^ In a search for uS12-binding partners, Strader et al. used tagged uS12 protein to copurify *Ec*YcaO and other proteins.^32^ Due to a minor reduction in uS12 modification status in a Δ*ycaO* strain (99% to 84%), *Ec*YcaO was proposed to contribute to uS12 β-methylthiolation (Figure S2). However, the role of *Ec*YcaO in this reaction is unclear, since RimO alone modifies a peptide substrate analog of the uS12 protein *in vitro* (Figure S2)^33,35^ and Δ*rimO* completely lacked uS12 modification *in vivo*.^32^ Several crystal structures of *Ec*YcaO have been solved (PDB: 4Q84, 4Q85, 4Q86), yet its physiological substrate and the functional impact of the modification have remained unknown.^36^ Furthermore, the absence of a nearby small open-reading frame suggests that *Ec*YcaO is unlikely to act on a RiPP precursor peptide.^36^

Here, we utilize several orthogonal approaches to establish *Ec*YcaO as the enzyme responsible for *Ec*uL16 thioamidation. First, we identified *Ec*YcaO and *Ec*uL16 as an interacting pair using unbiased proteome-wide structural predictions with AlphaFold3.^37^ We then isolated ribosomes from various deletion strains, showing the necessity of *Ec*YcaO for *Ec*uL16 thioamidation *in vivo*. Next, we reconstituted thioamidation *in vitro* using purified proteins, revealing that *Ec*YcaO requires a conserved tertiary structure to modify its target, in contrast to the majority of characterized YcaO enzymes, which recognize linear peptides. Leveraging the unusually low isoelectric point of *Ec*YcaO, we surveyed all prokaryotic genomes in GenBank to predict the pervasiveness of uL16 thioamidation. We hypothesize that uL16 thioamidation occurs in thousands of Gram-negative bacteria, including notable ESKAPE pathogens. Finally, we explored the physiological consequence of *Ec*uL16 Met82 thioamidation and uncovered a nonlinear epistatic interaction with Arg81 β-hydroxylation.

## Materials and Experimental Details

### General Materials and Methods

Reagents used for molecular biology experiments were purchased from New England BioLabs (Ipswich, MA), Thermo Fisher Scientific (Waltham, MA), or Gold Biotechnology Inc. (St. Louis, MO).*E. coli* DH5α and BL21­(DE3) strains were used for plasmid production and protein expression, respectively. Sanger sequencing was performed by the Core DNA Sequencing Facility at the University of Illinois at Urbana–Champaign. Whole-plasmid sequencing was performed by Plasmidsaurus. Matrix-assisted laser desorption/ionization time-of-flight mass spectrometry (MALDI-TOF-MS) analysis was performed using a Bruker UltrafleXtreme MALDI TOF-TOF mass spectrometer (Bruker Daltonics) in linear positive and reflector positive modes at the University of Illinois School of Chemical Sciences Mass Spectrometry Laboratory.

### AlphaFold3 Model Generation

All AlphaFold3 models were generated using the publicly available AlphaFold3 webtool (https://alphafoldserver.com/).^37^ The default parameters were used for each run, and the model with the highest interface predicted template modeling (ipTM) score was used each time. No cofactors, post-translational modifications, or higher-order complexes were accounted for in the proteome-wide screens.

### *E. coli* Ribosome Isolation

Crude ribosomes were isolated by ultracentrifugation as described.^38^ Briefly, *E. coli* cells were grown in LB medium to the exponential phase (OD_600_ ∼0.7) and then pelleted. All subsequent steps were carried out at 4 °C. The cell pellet was resuspended in buffer A [50 mM Tris, pH 7.5, 10 mM MgCl_2_, 0.1 M NH_4_Cl, 0.5 mM ethylenediaminetetraacetic acid (EDTA), 1 mM dithiothreitol (DTT)], 1× protease inhibitor cocktail [2 μM leupeptin, 2 μM benzamidine, 2 μM E64, and 30 mg phenylmethylsulfonyl fluoride (PMSF)] and lysed using a homogenizer. The lysate was clarified by centrifugation at 20,000 × *g* for 20 min. The resulting supernatant was carefully layered onto buffer B (50 mM Tris, pH 7.5, 10 mM MgCl_2_, 0.5 M NH_4_Cl, 0.5 mM EDTA, 36% sucrose, 1 mM DTT) and ultracentrifuged in a type 60 Ti rotor at 160,000 × *g* overnight. The ribosome pellet was resuspended in buffer C (50 mM tris, pH 7.5, 10 mM MgCl_2_, 0.1 M NH_4_Cl, 1 mM DTT) and stored at −80 °C until use.

### In-Gel Protease Digestions

In-gel digests of proteins were performed similarly to the following protocol.^39^ Briefly, bands of interest in a sodium dodecyl sulfate-polyacrylamide gel electrophoresis (SDS-PAGE) gel were excised using a clean razor blade and sliced into ∼1 × 1 mm^2^ cubes. These cubes were transferred into a 1.7 mL Eppendorf tube and submerged in 100 μL of a 1:1 mixture of 100 mM ammonium bicarbonate to acetonitrile for 30 min with occasional vortexing. Following this, 500 μL of neat acetonitrile was added. Once gel pieces became white and dehydrated, excess solvent was removed via rotary evaporation. A solution of ammonium bicarbonate (100 μL) containing either trypsin or endoproteinase GluC (13 ng μL^–1^) was added to resubmerge the gel pieces, which were then kept at 4 °C for 30 min. Once gel pieces were rehydrated, they were kept wet by the addition of excess 100 mM ammonium bicarbonate if necessary and kept at 4 °C for 2 h. Samples were then reacted at 37 °C for 18 h. Following this, the supernatant was directly utilized for analysis by MALDI-TOF-MS or further utilized in HR-MS/MS.

### High-Resolution Tandem Mass Spectrometry (HR-MS/MS)

The endoproteinase GluC-digested samples were analyzed by a ThermoFisher Scientific Orbitrap Fusion ESI-MS coupled with a Dionex Ultimate nanoUPLC system. Chromatography was performed with a flow rate of 0.3 μL/min. Solvent A: 0.1% formic acid in water, solvent B: 0.1% formic acid in 80% acetonitrile with water. Gradient: 0 min, 5% B; 10 min, 5% B; 20 min, 12.5% B; 65 min, 45% B; 70 min, 62.5% B; 74 min, 94% B; 77 min, 94% B; 88 min, 5% B; 100 min, 5% B. The MS was calibrated and tuned with Pierce LTQ Velos ESI Positive Ion Calibration Solution (ThermoFisher). The MS was operated using the following parameters: AGC target, 50,000; precursor scan resolution, 120,000; Orbitrap resolution (MS/MS): 50,000; quadrupole isolation window: 0.7; activation mode, HCD; collision energy stepped, 15, 20, 25%. Data analysis was conducted using the Qualbrowser application of Xcalibur software (ThermoFisher Scientific).

### Cloning

Cloning was performed using the Gibson method. Briefly, primers of 44–60 nucleotides in length were constructed to anneal the desired plasmids. DNA templates containing the vector of interest were amplified by PCR using these primers. To amplify *rplP*, *Ec*YcaO, and *Ec*RoxA DNA fragments, colony PCR was utilized using a single*E. coli* DH5α colony. Amplified DNA fragments were subjected to gel extraction (QIAquick gel extraction kit, Qiagen) and cloned into the pET28b or pRSFDuet vectors by Gibson assembly. Gibson constructs were transformed into chemically competent DH5α cells. All constructs were Sanger sequenced by the Core DNA Sequencing Facility at the University of Illinois at Urbana–Champaign to confirm successful mutagenesis.

### Hexahistidine-Tagged *Ec*uL16 Protein Expression and Purification

Plasmid vectors containing N-terminally hexahistidine-tagged *Ec*uL16 (His_6_-*Ec*uL16), C-terminally hexahistidine-tagged *Ec*uL16 (*Ec*uL16-His_6_), or variants thereof, were transformed into chemically competent BL21­(DE3) *E. coli* cells and grown on solid lysogeny broth Miller (LB) media containing 50 μg/mL kanamycin overnight. Single colonies were used to inoculate 10 mL of LB medium containing 50 μg/mL kanamycin and grown at 37 °C with shaking at 250 rpm. After 18 h, 1 L of LB was inoculated with the starter culture after addition of 50 μg/mL kanamycin. Cells were grown to an optical density (OD_600_) of 0.6–0.8. Cells were then placed on ice for 15 min, 0.4 mM (final) isopropyl β-d-1-thiogalactopyranoside (IPTG) was added, and the cultures grown for 3 h at 37 °C. Cells were harvested by centrifugation at 4,500 × *g* for 20 min, washed once with phosphate-buffered saline and recentrifuged at 4,500 × *g* for 20 min. Cell pellets were flash-frozen in liquid nitrogen and stored at −80 °C for a maximum of 1 week before use. For coexpression of *Ec*uL16 with either *Ec*YcaO or *Ec*RoxA, an identical procedure was followed.

For purification, cell pellets were thawed on ice and resuspended in 30 mL of lysis buffer [50 mM Tris, 0.5 M NaCl, 2.5% glycerol (*v/v*), pH 8.0] containing 4 mg/mL lysozyme, 2 μM leupeptin, 2 μM benzamidine, 2 μM E64, and 30 mg PMSF. Cells were then gently rocked for 30 min at 4 °C before 4 × 45 s of sonication, with 10 min of rocking between sonication rounds. Cellular debris was removed by centrifugation (20,000 × *g*, 90 min) and the supernatant was applied to pre-equilibrated nickel nitriloacetic acid (Ni-NTA) resin (1 mL of resin per L of cells). The column was washed with 10 column volumes (CV) of lysis buffer, then 10 CV of wash buffer (50 mM Tris, 1 M NaCl, and 30 mM imidazole, pH 8). His_6_-tagged proteins were eluted using 15 CV of elution buffer (50 mM Tris-HCl, 1 M NaCl, and 250 mM imidazole pH 8). Eluent was concentrated using an Amicon ultracentrifugal filter (EMD Millipore) with a 3 kDa molecular weight cutoff. Protein was buffer exchanged 1000-fold with protein storage buffer [50 mM 4-(2-hydroxyethyl)-1-piperazineethanesulfonic acid (HEPES), 300 mM NaCl, 2.5% glycerol (*v/v*) pH 7.5]. Protein concentrations were estimated using 280 nm absorbance, with theoretical extinction coefficients calculated using the ExPasy ProtParam Tool at: http://web.expasy.org/protparam.

### MBP-*Ec*YcaO Protein Expression and Purification

pET28a vectors containing MBP-*Ec*YcaO, or variants thereof, were transformed into chemically competent BL21­(DE3) *E. coli* cells and grown on solid lysogeny broth Miller (LB) media containing 50 μg/mL kanamycin overnight. Single colonies were utilized to inoculate 10 mL of LB medium containing 50 μg/mL kanamycin and grown at 37 °C with shaking at 220 rpm. After 18 h, 1 L of LB was inoculated with the starter culture after addition of 50 μg/mL kanamycin. Cells were grown to an optical density (OD_600_) of 0.6–0.8. Cells were then placed on ice for 15 min and 0.4 mM (final concentration) isopropyl β-d-1-thiogalactopyranoside (IPTG) was added. Cultures grown for 3 h at 37 °C. Cells were harvested by centrifugation at 4,500 × *g* for 20 min, washed once with phosphate-buffered saline and recentrifuged at 4,500 × *g* for 20 min. Cell pellets were flash-frozen in liquid nitrogen and stored at −80 °C for a maximum of 1 week before use.

For purification, cell pellets were thawed on ice and resuspended in 30 mL of lysis buffer [50 mM Tris-HCl, 0.5 M NaCl, 2.5% glycerol (*v/v*), and 0.1% triton X-100 (*v/v*) pH 7.5] containing 4 mg/mL lysozyme, 2 μM leupeptin, 2 μM benzamidine, 2 μM E64, and 30 mg PMSF. Cells were rocked for 30 min at 4 °C before 4 × 45 s of sonication, with 10 min of rocking between rounds of sonication. Cellular debris was removed by centrifugation (20,000 × *g*, 60 min) and the supernatant was applied to pre-equilibrated amylose resin (5 mL of resin per L of cells). The column was washed with 10 column volumes (CV) of lysis buffer, then 10 CV of wash buffer [50 mM Tris-HCl, 0.5 M NaCl, and 2.5% glycerol (*v/v*) pH 7.5]. MBP-tagged proteins were eluted using 5 CV of elution buffer [50 mM Tris-HCl, 300 mM NaCl, 10 mM maltose, and 2.5% glycerol (*v/v*) pH 7.5]. Eluent was concentrated using an Amicon ultracentrifugal filter (EMD Millipore) with an appropriate molecular weight cutoff. Protein was buffer exchanged with 10× volume of protein storage buffer [50 mM 4-(2-hydroxyethyl)-1-piperazineethanesulfonic acid (HEPES), 300 mM NaCl, 2.5% glycerol (*v/v*) pH 7.5]. Protein concentrations were estimated using both Bradford assay and by 280 nm absorbance, with theoretical extinction coefficients calculated using the ExPasy ProtParam Tool.

### Oxidation of *Ec*uL16 Endoproteinase GluC-Derived Peptide

Briefly, endoproteinase GluC-digested *Ec*uL16 peptide was mixed with 1 mM H_2_O_2_ and allowed to react for 5 min at ambient temperature. Following this, the reaction was analyzed by MALDI-TOF-MS.

### *In Vitro* Thioamidation of *Ec*uL16 by *Ec*YcaO

*In vitro* assays were performed using 1 μM MBP-*Ec*YcaO with full-length nonthioamidated uL16 purified from BL21­(DE3) Δ*ycaO* in a total of 50 μL of 50 mM Tris pH 7.5, 10 mM MgCl_2_, 1 mM ATP, and 1 mM Na_2_S. Reactions were allowed to proceed at 37 °C overnight and quenched by the addition of 5 μL of a 20% SDS solution. The reaction mixture was resolved by SDS-PAGE, and the target uL16 band was excised, subjected to in-gel digestion with endoproteinase GluC, and analyzed by MALDI-TOF-MS.

### Construction of *E. coli* In-Frame Gene Deletion Mutants

*E. coli* in-frame gene deletions were constructed using the established λ-Red recombination system.^40^ The primers used for gene deletion purposes are listed in Table S4. An apramycin-resistant Δ*ycaO* mutant was derived from *E. coli* BL21­(DE3). *E. coli* BL21­(DE3) Δ*ycaO*/Δ*roxA* mutant was generated from the Δ*ycaO* strain, yielding a strain that was also spectinomycin-resistant. *E. coli* K12 Δ*ycaO*/Δ*roxA* was constructed from BW25113 Δ*ycaO* (kanamycin-resistant, Keio collection),^41^ which resulted in a strain that was also chloramphenicol-resistant. All genotypes were verified by PCR and by whole genome sequencing (Plasmidsaurus).

### Site-Directed Mutagenesis

Site-directed mutagenesis was performed using the QuikChange method. Briefly, PCR was performed using Q5 high-fidelity polymerase and long primers containing the appropriate codon mutation(s). Crude reactions (10 μL) were analyzed using agarose (0.7%) gel electrophoresis to confirm a linear product of the expected size. If the band was not observed, 10 μL of GC enhancer and/or betaine (5 M stock conc.) were used to supplement the PCR in further attempts. Once the expected band was confirmed, reactions were digested with 1 μL of DpnI overnight and transformed into chemically competent DH5α cells. All constructs were Sanger sequenced by the Core DNA Sequencing Facility at the University of Illinois at Urbana–Champaign to confirm successful mutagenesis.

### Fluorescent-Labeling of *Ec*uL16

C-terminal 5-iodoacetamidofluorescein (5-IAF) labeling of *Ec*uL16 was conducted according to the manufacturer’s instructions (Thermo Scientific). Briefly, reduced *Ec*uL16-His_6_-Cys was reacted with a 10-fold molar excess of 5-IAF in 200 mM ammonium bicarbonate at room temperature. The labeling progress was monitored using LC-MS. Excess 5-IAF was removed by dialysis through a PD-10 column. The labeled *Ec*uL16 was diluted in a binding buffer [20 mM HEPES, pH 7.5, 150 mM NaCl, 2.5% (v/v) glycerol] to prepare a stock concentration of 100 nM for subsequent binding assays.

### Fluorescence Polarization (FP)

The binding of 5-IAF labeled uL16 to MBP-*Ec*YcaO proteins was assessed by equilibrium FP at room temperature in nonbinding-surface, 384-black-well polystyrene microplates (Corning) using a Spark plate reader (Tecan) with default settings. For each titration, MBP-*Ec*YcaO or its variants were serially diluted into the binding buffer, mixed with 2 nM fluorescein-labeled *Ec*uL16, and equilibrated for 0.5 h with shaking at room temperature before data acquisition. For competitive assays, a constant MBP-*Ec*YcaO concentration (around *K*_d_) was mixed with 2 nM labeled *Ec*uL16 in the binding buffer. Serial dilutions of nonlabeled *Ec*uL16 variants were then titrated and treated as described above. Data from triplicate titrations were background-subtracted and fit to a nonlinear dose–response curve in OriginPro 2024b (OriginLab) to calculate the dissociation constants (*K*_d_) and half-maximal inhibitory concentration (IC_50_).

### Expression and Analysis of *Ec*uL16 Ala Variants

*E. coli* BL21­(DE3) was used to overexpress each His_6_-*Ec*uL16 Ala variant as described in the above section, with the following change. Growth medium LB (250 mL) was used to overexpress each variant. Variants were purified as described above and in-gel digested with trypsin and/or GluC. In every sample other than the R81A variant, trypsin produces a fragment on which RoxA-catalyzed hydroxylation is isolated from YcaO-catalyzed thioamidation, while GluC digestion produces a fragment where both modifications can be observed.

### Bioinformatic Analysis of YcaO Proteins

Briefly, a sequence similarity network (SSN) of the YcaO protein superfamily (IPR003776) was constructed using UniRef90. This SSN was visualized using Cytoscape^42^ and at an alignment score of 47. The isoelectric point (pI) of each node’s primary sequence was determined using the standard Henderson–Hasselbalch charge-balance method, in which charges from the N-terminus, C-terminus, and seven titratable side chains were accounted for.

### Heterologous Expression and Analysis of uL16 and YcaO Encoded in *Klebsiella pneumoniae* and *Pseudomonas aeruginosa*

Genes encoding uL16 and YcaO from *K. pneumoniae* and *P. aeruginosa* were cloned into pRSFDuet vectors. N-terminally His_6_-tagged uL16 proteins were recombinantly expressed either alone or in combination with their cognate YcaO proteins (untagged) in *E. coli* BL21­(DE3) Δ*ycaO*/Δ*roxA*. uL16 proteins were purified using Ni-NTA resin, visualized by Coomassie blue-staining after SDS-PAGE analysis, and subjected by in-gel digestion with endoproteinase GluC. The resulting digested products were analyzed by MALDI-TOF and HR-MS/MS.

### Growth Curve Assays

Growth curves (OD_600_) were monitored for*E. coli* K-12 BW25113 WT, Δ*ycaO*, Δ*roxA*, and Δ*ycaO*/Δ*roxA.* Continuous growth monitoring was performed in a 96-well plate using a Tecan Spark plate reader, as previously described.^43^ Briefly, freshly transformed colonies were inoculated into 0.5 mL of LB medium and grown overnight at 37 °C with shaking at 250 rpm. Aliquots (1 μL) of each overnight culture were diluted into 200 μL of fresh medium, either LB or M9 (containing 0.4% glucose (w/v), 1× M9 salts (BD Difco), 1 mM MgSO_4_, and 0.1 mM CaCl_2_). Cultures were incubated at 37 °C with moderate orbital shaking. OD_600_ measurements were recorded every 10 min, with well-specific blanks (LB and M9) used to correct for background. Each strain was tested in 3 biological replicates. Growth curves were processed and visualized using Origin 2024b software.

## Results

### *Ec*YcaO Proteome-Wide AlphaFold3 Scanning

Most genomically predicted enzymes lack an identifiable substrate.^44^ While prokaryotic RiPPs simplify this challenge by encoding the substrate near the enzyme, *Ec*YcaO has no nearby short peptide-encoding gene, leaving its natural partner unknown. However, *Ec*YcaO has several experimentally determined, high-resolution structures available that defined a unique ATP-binding site.^36^ The YcaO catalytic mechanism has also been extensively investigated.^25,23,45^ Recent publications have shown that AlphaFold3 and related algorithms have utility in predicting protein–protein interactions.^46–48^ Given this background, we asked if AlphaFold3 could detect *Ec*YcaO interacting proteins and if the orientation of the interacting partner could suggest catalytic competence.

Based on prior literature, we suspected that *Ec*YcaO may interact with *Ec*uL16, as it was the most likely enzyme responsible for thioamidation of Met82.^12^ However, an earlier publication implicated *Ec*uS12 as a potential interacting partner.^32^ Since these are both ribosomal subunit proteins, we individually assessed all 55 ribosomal subunit proteins from *E. coli* against *Ec*YcaO. The first *in silico* protein–protein interaction screen included ATP and 2 Mg^2+^ ions modeled into the *Ec*YcaO active site, consistent with the known structure (PDB: 4Q85). However, the resulting interface predicted template modeling (ipTM) scores indicated that many of the ribosomal proteins confidently interacted with EcYcaO, with only bS1 not scoring as a plausible interactor (Figure S3). An unexpected correlation between the ipTM score and the size of the query protein was observed. Suspecting that YcaO’s cofactors may have inflated the confidence score, we performed an otherwise identical scan that excluded ATP and Mg^2+^, which alleviated the issue (Figure S4). Thus, all future modeling was performed without these cofactors. From the initial panel, we noted that only two ribosomal subunit proteins produced a confident protein–protein interaction (ipTM score) with *Ec*YcaO, specifically *Ec*uL16 and *Ec*uL29 (Figure S5). Separately, complexation of *Ec*uS12 and *Ec*RimO with *Ec*YcaO gave nonconfident scores (ipTM = 0.31 and 0.16, respectively), suggesting that if *Ec*YcaO interacts with these proteins, it may be an indirect association (Figures S6–S7). Inspection of the *Ec*uL29 complex revealed a predicted association with the outer face of *Ec*YcaO and no part of *Ec*uL29 was plausibly positioned within the active site (ipTM = 0.8) (Figure S5). Notably, *Ec*uL29 has been previously reported to interact with *Ec*YcaO.^49^ In contrast, the model of *Ec*uL16 showed insertion of an extended loop into the *Ec*YcaO active site (ipTM = 0.8). This loop includes the known site of thioamidation, Met82. When ATP and 2 Mg^2+^ ions were included (ipTM = 0.87) (Figure S8) Met82 was positioned in a biochemically suggestive manner with the γ-phosphate of ATP, which itself is flanked by four biochemically validated ATP-binding residues.^36^ The *Ec*uL16-*Ec*YcaO interaction surface extends significantly beyond loop insertion, with broad surfaces of each protein appearing to form a complex protein–protein interaction.

We then expanded the AlphaFold3 screen to individually model each of the 4,403 proteins encoded by the *E. coli* K12 proteome against *Ec*YcaO. The resulting ipTM score of the top model produced for each query protein was tabulated (Figure 2 and Supporting Data Set 1). Only 3 out of 4,403 (0.068%) proteins produced confident ipTM values, two of which were already identified in the panel that assessed ribosomal subunit proteins (i.e., *Ec*uL16 and *Ec*uL29). The third protein was *Ec*YcaO itself, which lends more credence to this method as *Ec*YcaO is a known homodimer (Figure 2).^36^ While numerous models produced plausible ipTM values between 0.6 and 0.8 (Table S2), none have known involvement with *Ec*YcaO. Upon rescanning the plausible hits against homodimeric *Ec*YcaO, no significant trends were observed; however, it was notable that a trimeric model of YcaO received a substantially reduced score (Table S3). Furthermore, manual inspection of all models with plausible ipTM scores revealed that none were positioned within the active site of *Ec*YcaO, supporting the hypothesis these are unlikely substrates for *Ec*YcaO.

**Figure 2:**
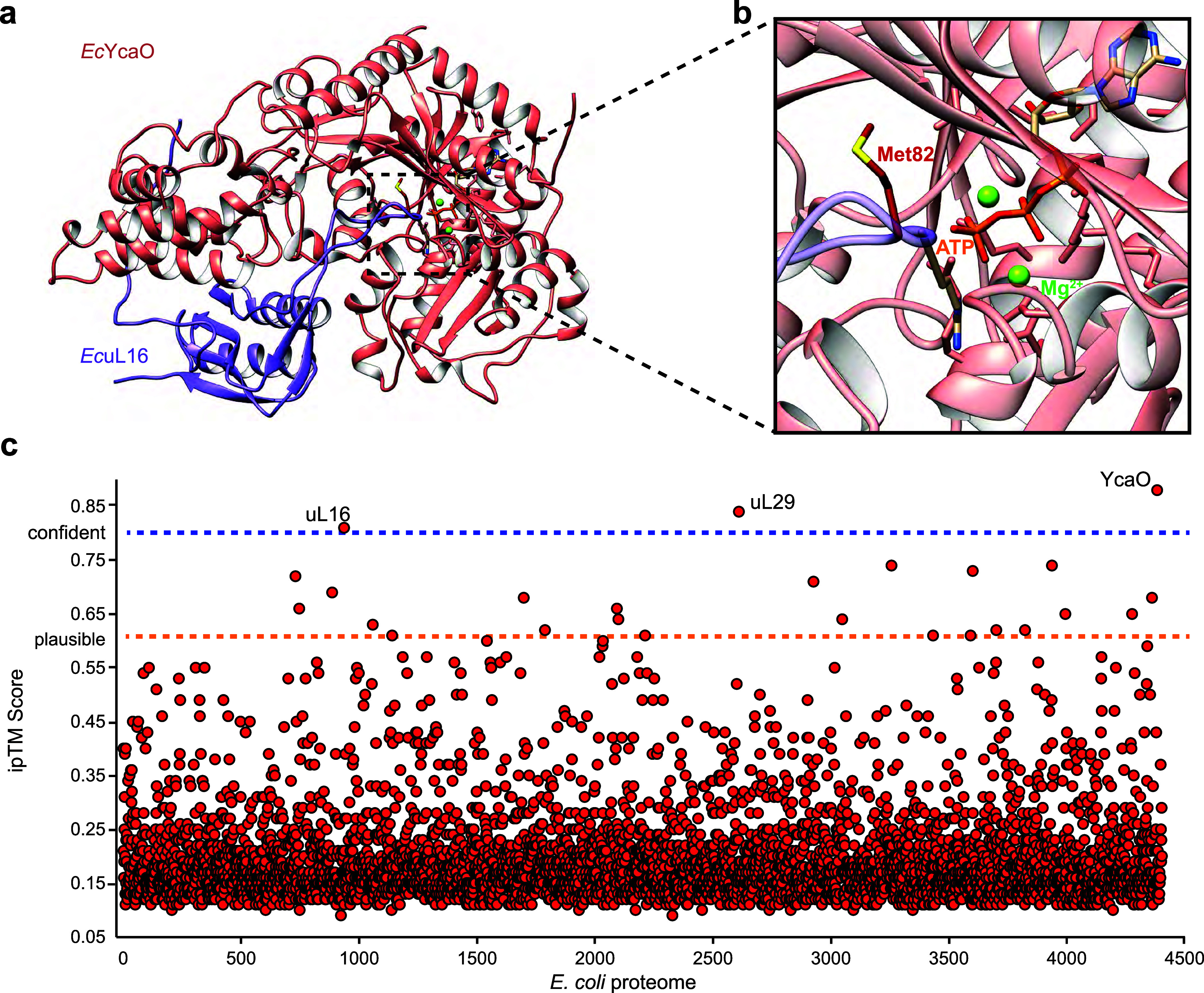
(a) AlphaFold3 model of *Ec*YcaO (salmon) and *Ec*uL16 (purple). The inset (b) displays the location of Met82 in *Ec*uL16, with the side-chain in fire-brick red. ATP is in orange, and Mg^2+^ ions are depicted as green spheres. (c) Proteome-wide AlphaFold3 predictions using *Ec*YcaO as the bait protein. The interface predicted template modeling (ipTM) score of each protein in the *E. coli* proteome (*n* = 4,403), when modeled with *Ec*YcaO, is displayed. Proteins with an ipTM value ≥ 0.8 are labeled (Figures S3 and S6).

To more extensively evaluate the reliability of the proteome-wide interaction survey, we inverted the analysis, this time using *Ec*uL16 as the query protein (Figure S9 and Supporting Data Set 2). This reciprocal screen produced three confident candidates: PncC, EbgC, and *Ec*YcaO (Figures S10–S11). Retrieval of *Ec*YcaO as a top hit in this screen was expected. However, PncC, a nicotinamide-nucleotide amidohydrolase (Uniprot: P0A6G3), and EbgC, a subunit of the evolved β-galactosidase (Uniprot: P0AC73), lack any plausible rationale for association with uL16 and likely represent false-positives.^50,51^ Neither model places uL16 in a catalytically suggestive pose. Notably, *Ec*RoxA (UniProt: P27431), the enzyme that hydroxylates uL16-Arg81, yielded a plausible ipTM score of 0.57 (Figure S12). Additionally, the loop of uL16 that contains Arg81 is oriented into the active site of RoxA. While this computational method can certainly capture bona fide interacting partners, the accuracy is imperfect, as illustrated here.

### *ycaO* Is Required for *Ec*uL16 Thioamidation

With increased confidence that *Ec*YcaO is responsible for *Ec*uL16 thioamidation, we compared ribosomes from wild-type (WT) *E. coli* BW25113 and the corresponding Δ*ycaO* Keio strain.^52^ Matrix-assisted laser desorption/ionization time-of-flight mass spectrometry (MALDI-TOF-MS) analysis of purified ribosomal proteins revealed a mass difference of ∼16 Da between the *Ec*uL16 proteins from the two strains (Figure S13). To localize the modification, an in-gel digestion was performed using endoproteinase GluC, which was predicted to yield a ∼1600 Da peptide more suitable for analysis than the short (3-residue) tryptic fragment containing Met82. Endoproteinase GluC fragments confirmed the absence of thioamidation at *Ec*uL16-Met82 in the Δ*ycaO* strain (Figure 3). High-resolution tandem mass spectrometry (HR-MS/MS) further distinguished between the loss of a thioamide (calculated error = 0.1 ppm) and loss of an oxygen atom (calculated error = 11.2 ppm), strongly supporting the former assignment (Figures S14 and S15). While hydroxylation of Arg81 by *Ec*RoxA is isobaric with Met82 oxidation, we also examined uL16 from a Δ*roxA* strain^52^ and found that thioamidation persisted in the absence of hydroxylation (Figure S13). Together, these results establish that uL16 thioamidation requires *Ec*YcaO.

**Figure 3:**
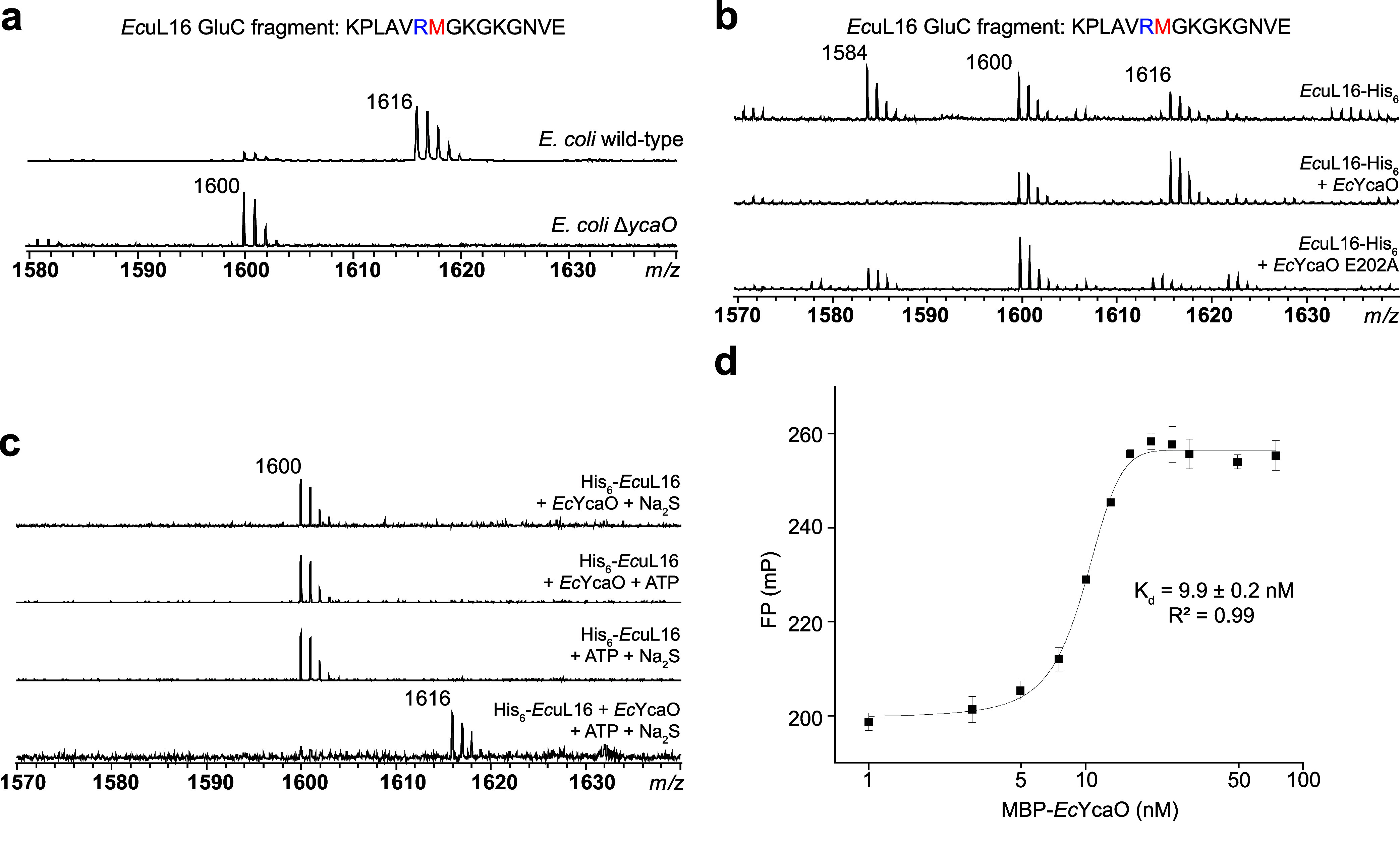
(a) MALDI-TOF-MS data displaying GluC-digested *Ec*uL16 isolated from crude ribosomes from wild-type and Δ*ycaO* *E. coli*. Expected *m*/*z* values for unmodified uL16 fragment, hydroxylated or thioamidated fragment, and hydroxylated and thioamidated fragment, are 1584, 1600, and 1616, respectively. (b) MALDI-TOF-MS data from the overexpression and GluC- digestion of His_6_-tagged *Ec*uL16 in *E. coli* BL21 (DE3), coexpressed with either *Ec*YcaO or *Ec*YcaO-E202A. The *m*/*z* 1614 peak likely represents a missed GluC cleavage corresponding to residues 136–150 of *Ec*YcaO, which copurifies with *Ec*uL16-His_6_. (c) MALDI-TOF-MS of *in vitro*, GluC-digested *Ec*uL16 thioamidation reactions utilizing *Ec*YcaO. Expected *m*/*z* values for unmodified uL16 fragment, hydroxylated or thioamidated fragment, and hydroxylated and thioamidated fragment, are 1584, 1600, and 1616, respectively. (d) Fluorescence polarization data for fluorescein-labeled *Ec*uL16-His_6_Cys with MBP-*Ec*YcaO.

For the sake of rigor, we additionally analyzed *Ec*uL29 in both the Δ*ycaO* and Δ*roxA* strains by MALDI-TOF-MS. No mass shift was observed for *Ec*uL29 (Figure S13). Thus, while the AlphaFold3 model exhibits a confident ipTM score, there is no evidence *Ec*uL29 is modified by *Ec*YcaO or *Ec*RoxA.

### *Ec*YcaO Directly Thioamidates *Ec*uL16

To assess whether *Ec*YcaO directly thioamidates *Ec*uL16, we overexpressed *Ec*uL16 in BL21 (DE3) with either an N-terminal or a C-terminal His tag (His_6_-*Ec*uL16 and *Ec*uL16-His_6_, respectively) (Table S4). After immobilized metal affinity chromatography (IMAC) purification (Figure S16), the proteins were digested with endoproteinase GluC and analyzed by MALDI-TOF-MS (Figure S17). Both variants displayed Arg81 hydroxylation and Met82 thioamidation, although the C-terminal tag modestly reduced the extent of modification. To confirm that Met82 carried a thioamide rather than an oxidation product, samples were briefly treated with hydrogen peroxide.^53^ This generated a +16 Da peak (*m*/*z* 1632), consistent with Met *S*-oxidation, thereby validating the prior presence of Arg81 hydroxylation and Met82 thioamidation (Figure S17).

We suspected that the incomplete modification of *Ec*uL16 reflected limiting levels of native YcaO and RoxA. Therefore, we overexpressed *Ec*YcaO and *Ec*uL16-His_6_ (Figure S18), followed by IMAC and endoproteinase GluC digestion. Analysis by MALDI-TOF-MS revealed complete thioamidation of Met82, providing further evidence that *Ec*YcaO catalyzes thioamidation of *Ec*uL16 (Figure 3). Analogously, coexpression of *Ec*RoxA and *Ec*uL16-His_6_ resulted in complete β-hydroxylation of Arg81 in *Ec*uL16-His_6_ (Figure S18). Notably, untagged *Ec*YcaO and *Ec*RoxA were both copurified by His_6_-tagged *Ec*uL16 (Figures S19–S21), underscoring that both enzymes have noticeable affinity for *Ec*uL16. Replacement of any ATP-binding residues^36^ in *Ec*YcaO and simultaneous overexpression of this variant with *Ec*uL16-His_6_ resulted in incomplete thioamidation (Figures 3 and S22). Additionally, expression of His_6_-*Ec*uL16 in *E. coli* BL21 (DE3) Δ*ycaO*, and *ΔycaO/*Δ*roxA*, constructed via standard λ-Red recombination,^54^ reaffirmed the expected activity of both enzymes. Hydroxylation was only observed with the addition of *Ec*RoxA, and thioamidation with *Ec*YcaO (Figure S23). Thus, *Ec*YcaO is responsible for *Ec*uL16 thioamidation.

We next sought to reconstitute *Ec*YcaO-mediated thioamidation *in vitro*. Previously, we demonstrated that a methanogen-derived, thioamide-forming YcaO could modify short peptides from McrA that contained the thioamidated residue.^29^ *Ec*YcaO was expressed and purified as an N-terminal maltose-binding protein (MBP) fusion (Figure S16). We initially attempted to reconstitute thioamidation using short peptide substrates. Neither an 11-residue peptide, consisting of the 5 residues on each side of Met82 (*Ec*uL16_77–87_), nor a 21-residue peptide, consisting of the 10 residues on each side (*Ec*uL16_72–92_), was modified under our reaction conditions (Figure S24). However, reactions using full-length His_6_-*Ec*uL16 isolated from *E. coli* BL21 (DE3) Δ*ycaO* resulted in thioamidation only when the reaction was supplied with Na_2_S, MgCl_2_, and ATP (Figure 3). Endoproteinase GluC-digested *Ec*uL16 was not modified when provided as a mixture of peptides (Figure S25), suggesting that *Ec*YcaO recognizes the tertiary structure of *Ec*uL16 rather than a linear motif near Met82.

### *Ec*YcaO Recognizes the Tertiary Structure of *Ec*uL16

Many YcaO enzymes utilize a partner protein containing a RiPP Recognition Element (RRE)^55^ to bind their substrate peptide.^23,56,57^ *Ec*YcaO does not contain an RRE, but other YcaO superfamily members recognize a short stretch of residues within the vicinity of the modified residue.^28,29^ As shown above, *Ec*YcaO binds *Ec*uL16 directly (Figures S19 and S20). Coupled with the necessity of full-length, folded protein for *in vitro* modification, we suspected the binding interaction may encompass a larger area of *Ec*uL16. To this end, we assessed *Ec*uL16 binding to *Ec*YcaO. First, residues 70–93 of *Ec*uL16 were synthesized via solid-phase peptide synthesis (SPPS) with an N-terminal fluorescein isothiocyanate (FITC) group tethered by a 6-aminohexanoic acid linker (FITC-*Ec*uL16_70–93_). These residues comprise the long loop of uL16, with Arg81 and Met82 appearing at the point most distal to the globular fold (Figure S26). These positions extend into the tRNA-binding corridor when *Ec*uL16 is ribosome-bound.^12^ Upon titration of FITC-*Ec*uL16_70–93_ with MBP-*Ec*YcaO, no interaction was detected by fluorescence polarization (FP). This result demonstrates that residues 70–93 of *Ec*uL16 are insufficient to bind *Ec*YcaO, which was expected given the necessity of full-length *Ec*uL16 for *in vitro* modification by *Ec*YcaO (Figures S24 and S25). We then prepared *Ec*uL16-His_6_Cys, which contains a single Cys immediately after the C-terminal hexahistidine tag (Figure S16). After expression in *E. coli* BL21 (DE3) *ΔycaO/*Δ*roxA*, the protein was purified and then labeled with 5-iodoacetofluorescein (5-IAF). FP-based binding assays between labeled *Ec*uL16-His_6_Cys and MBP-*Ec*YcaO revealed a significant interaction (*K*_d_ = 9.8 ± 0.2 nM) (Figure 3).

Most characterized YcaO enzymes modify RiPP precursor peptides, which are highly sequence divergent. In contrast, *Ec*uL16 is highly conserved. Given this, we assessed whether individual residues of *Ec*uL16 played a significant role in the binding interaction. We performed an Ala scan of representative residues that residue within the loop region of *Ec*uL16. Each Ala variant was expressed and purified from *E. coli* BL21 (DE3) *ΔycaO/*Δ*roxA*, and the affinity toward MBP-*Ec*YcaO assessed via competitive FP (Table 1 and Figure S27,). Notably, His_6_-*Ec*uL16 gave a *K*_i_ value of ∼39 nM (Table 1), indicating weaker binding than *Ec*uL16-His_6_Cys. Most other examined variants showed only 2–3-fold weaker association to *Ec*YcaO under the conditions tested, supporting that the interaction occurs over a more extensive region of the two proteins. However, the uL16-G85A variant exhibited the most perturbed binding, with a *K*_i_ value >200 nM (Table 1).

**Table 1:** *K*_i_ Values for Ala Variants of His_6_-*Ec*uL16^a^

uL16 Variant	*K*_i_ (nM)
Wild-type	39 ± 3
P72A	77 ± 12
T74A	76 ± 6
L78A	49 ± 4
R81A	91 ± 20
M82A	92 ± 4
G83A	71 ± 4
G85A	>200
V89A	116 ± 13

a Variants were assessed via competitive FP for binding to MBP-*Ec*YcaO. The inhibitory constants are reported as the average with error given as the standard error of the mean (*n* = 3).

The catalytic activity of *Ec*YcaO was then tested against the panel of Ala-substituted uL16 loop variants through *in vivo* expression in *E. coli* BL21 (DE3). To assess modification status, we required a method that would unambiguously delineate nearly isobaric modifications on adjacent residues. We surmised that parallel digestions with trypsin and endoproteinase GluC would solve this issue, given that trypsin cleaves between Arg81 and Met82, while GluC generates a fragment containing both potential modification sites. Upon performing the digests and analyzing the mass spectral data, the uL16-G85A variant was effectively modified by *Ec*YcaO despite having a lower affinity (Tables S5, S6 and Figures S28, S29). Qualitatively, the G83A variant was a poor substrate for both *Ec*RoxA and *Ec*YcaO. We further noted that the *Ec*uL16-R81A was thioamidated but not hydroxylated, and the *Ec*uL16-M82A variant was both hydroxylated and thioamidated.

To further assess the interaction with, *Ec*uL16, we prepared 12 Ala-substituted variants of *Ec*YcaO at various positions with plausible involvement at the binding interface (Figures S16 and S30) and assessed their capacity to bind to labeled *Ec*uL16-Cys-His_6_ through direct FP (Table 2 and Figure S31). Each Ala variant bound tightly to *Ec*uL16-Cys-His_6_ (*K*_d_ < 20 nM). These data indicated that the individual contribution of a single residue was minimal to the overall binding interaction.

**Table 2:** *K*_d_ Values for Ala Variants of MBP-*Ec*YcaO^a^

YcaO-Variant	*K*_d_ (nM)
Wild-type	9.8 ± 0.2
L55A	17.3 ± 0.7
F57A	13.8 ± 0.5
R79A	15.9 ± 0.6
D88A	13.6 ± 1.0
R295A	11.1 ± 0.7
T317A	10.9 ± 0.8
E320A	11.3 ± 0.5
D325A	10.7 ± 0.6
L329A	9.7 ± 0.4
R444A	13.5 ± 0.6
R463A	14.9 ± 1.1
F491A	14.8 ± 1.1

a Variants were assessed via FP for binding to FITC-labeled EcYcaO-His_6_Cys. The dissociation constants are reported as the average with error given as the standard error of the mean (*n* = 3).

### YcaO-Mediated uL16 Thioamidation is Widespread

To assess the frequency of uL16 thioamidation among prokaryotes, we generated a sequence similarity network (SSN)^58^ of all available YcaO enzymes in UniProt release 2025_03. At an alignment score of 47, *Ec*YcaO groups with 1,225 predicted YcaO proteins at a high convergence score (Figure 4). A distinctive feature of *Ec*YcaO is its low isoelectric point (pI = 4.4) (Figure 4). This correlates with the substrate of *Ec*YcaO being *Ec*uL16 which has a high isoelectric point (pI = 11.2). The available crystallographic and modeling data indicate that the acidic residues of *Ec*YcaO localize primarily within the active site pocket and the surface of the protein that interacts with the highly basic region of *Ec*uL16 (Figure 4). We hypothesized that electrostatic complementarity would be conserved among any YcaO that performs uL16 thioamidation. Leveraging this distinctive feature, we colored the SSN according to pI of the YcaO enzyme. Doing so revealed that the majority of YcaO enzymes that group with *Ec*YcaO are highly acidic relative to the average YcaO (Figures 4 and S32). We suspect that acidic YcaO enzymes in this group perform uL16 thioamidation in their host organisms. A few examples of YcaO enzymes that group with *Ec*YcaO are not highly acidic. Analysis of the local genomic context of these YcaO enzymes strongly suggests involvement in canonical RiPP biosynthesis (Supporting Data Set 3 and Figure S33).

**Figure 4:**
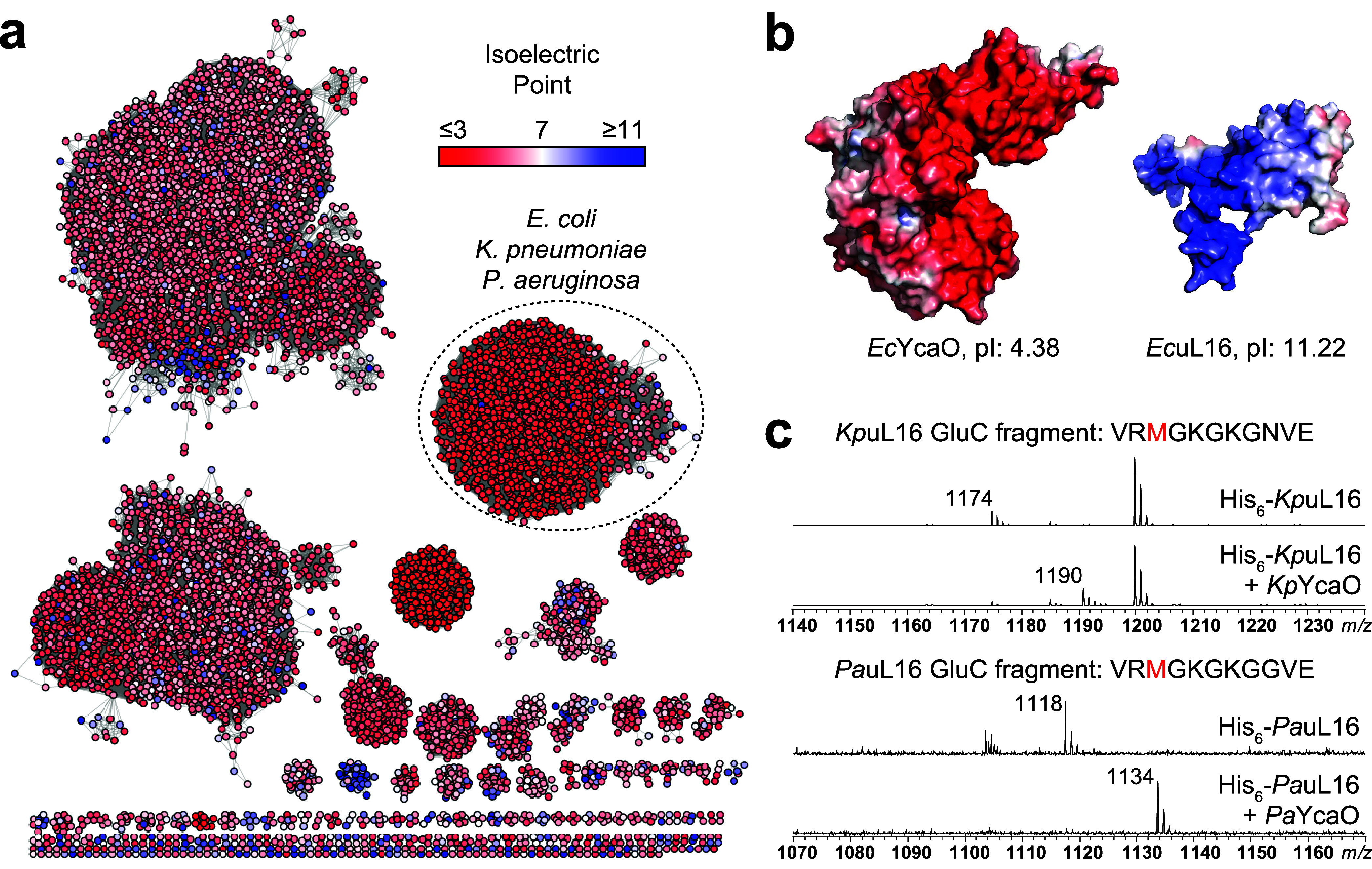
Predicting the prevalence of uL16 thioamidation in bacteria. (a) Sequence similarity network of IPR003776 (alignment score 47, RepNode 90). The isoelectric point value was mapped onto each node and colored as indicated. The circled group contains predominantly acidic YcaOs likely associated with uL16 in other organisms. (b) Electrostatic surface potential representation of *Ec*YcaO and *Ec*uL16. (c) uL16 proteins were expressed either alone or in combination with their associated YcaO enzyme in *E. coli* BL21 (DE3) *ΔycaO/*Δ*roxA*, digested with endoproteinase GluC, and analyzed by MALDI-TOF-MS. For *Kp*uL16, the unmodified fragment *m*/*z* is 1174 Da, while the thioamidated fragment is 1190. For *Pa*uL16, the unmodified fragment *m*/*z* is 1118 Da, while the thioamidated fragment is 1134.

To evaluate if other proteins in the *Ec*YcaO SSN group modify uL16, we cloned the uL16-encoding genes from *K. pneumoniae* (i.e., *Kp*uL16 [UniProt: A6TEW5] [98% similar, 97% identical to *Ec*uL16]) and *P. aeruginosa* (i.e., *Pa*uL16 [UniProt: Q9HWE2] [86% similar, 77% identical to *Ec*uL16]). These genes were expressed either alone or in combination with their cognate YcaO-encoding genes in*E. coli* *BL21­(DE3) ΔycaO/*Δ*roxA*. The endoproteinase GluC fragments of *Kp*uL16 and *Pa*uL16 displayed a + 16 Da shift only when coexpressed with *Kp*YcaO (UniProt: A6T6Z8) and *Pa*YcaO (UniProt: Q9I2H8), respectively (Figure 4). Thioamidation was unambiguously assigned to the expected Met residues in each fragment by HR-MS/MS (Figures S34 and S35).

### Functional Insight into *Ec*uL16 Modifications

The fitness ramifications of *Ec*uL16 thioamidation are unknown. While a few minor phenotypic consequences were noted for Δ*ycaO* during a genome-wide deletion assessment of*E. coli*,^59^ there is no direct evidence that uL16 thioamidation governs these phenotypes. To assess the role of this modification, we first compared the growth rates of *E. coli* BW25113 to an otherwise identical strain lacking *ycaO*. In both nutrient-rich (LB) and -poor media (M9), the growth rates were identical (Figure 5). Given the proximity of the thioamide to the β-hydroxyl group installed on Arg81, we then assessed growth rates for Δ*roxA* and *ΔycaO/*Δ*roxA* strains. Previous work demonstrated that deletion of *roxA* resulted in a growth rate comparable rate to the parental strain under nutrient-rich conditions but significantly reduced growth rate using a nutrient-poor medium.^13^ Intriguingly, the *ΔycaO/*Δ*roxA* strain restored the growth rate to WT levels (Figure 5). This epistatic effect between uL16 thioamidation and hydroxylation implies that these post-translational modifications interact to finely tune the translational apparatus, and that simply having the thioamide without the adjacent hydroxyl group may be responsible for the slower growth rate in nutrient-poor media. Furthermore, the co-occurrence of the putatively uL16-associated *ycaO* and *roxA* genes supports a synergistic role (Figure S36).^60^ A deeper investigation into the precise mechanism behind this phenotype is ongoing.

**Figure 5:**
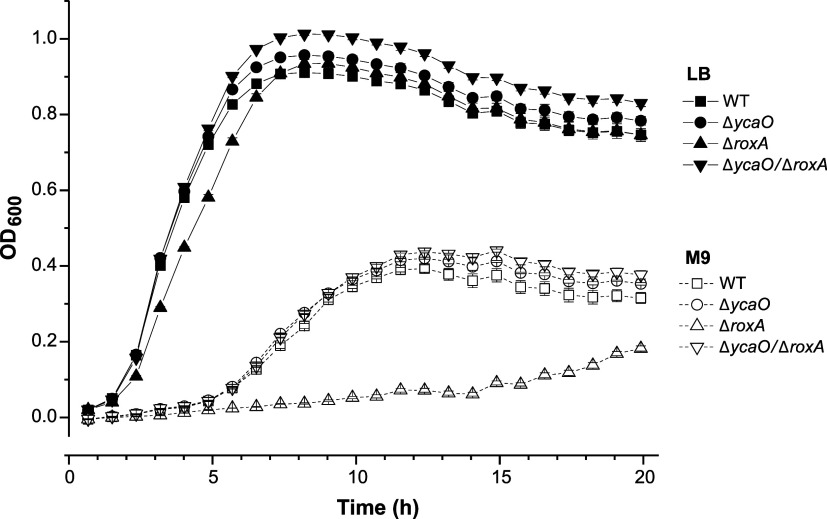
Growth curves of *E. coli* WT and deletion strains. Bacteria were grown for 20 h in either nutrient-rich (LB, filled symbols) or nutrient-poor (M9, open symbols) media. Data are plotted as average values with error given as standard error of the mean (*n* = 3).

## Discussion

Our results unambiguously establish *Ec*YcaO as the enzyme responsible for *Ec*uL16-Met82 thioamidation. Taken together with MCR, this work provides the second example of YcaO-catalyzed thioamidation of a protein, a modification which now spans both the archaeal and bacterial domains. YcaO modifications are traditionally attributed to RiPP biosynthetic pathways such as linear azol­(in)­e-containing peptides, cyanobactins, thiopeptides, bottromycins, and thioamitides.^23,31^ However, our work suggests that additional protein substrates for YcaO remain undiscovered, even in well characterized organisms. Structural alignment of *Ec*YcaO with several structurally characterized YcaO enzymes involved in RiPP biosynthesis reveals a 150-residue tetratricopeptide repeat domain that mediates dimerization and might also contribute to *Ec*uL16 binding (Figure S37).

Out of 4,403 proteins, a proteome-wide AlphaFold3 protein–protein interaction analysis successfully identified three confident YcaO-interacting partners. While *Ec*YcaO was previously postulated as a candidate for uL16 thioamidation,^12^ this method supported uL16 as a compelling candidate both for YcaO engagement and catalytic competence. To test the method further, we performed the reciprocal analysis and evaluated the *E. coli* K12 proteome against *Ec*uL16. Only two false-positives and several false-negatives were apparent (Supporting Data Set 2). A lack of ribosomal proteins indicates that more than two may be needed to successfully predict a model, and/or that inclusion of 23S rRNA is critical for accurate predictions.

Notably, inclusion of known YcaO cofactors ATP and Mg^2+^ significantly raised the model ipTM score, perhaps due to their inclusion in YcaO structures deposited in the Protein Data Bank. We caution against the sole use of ipTM score in interpreting models with cofactors included, as it may misrepresent confidence in the protein–protein interface.^61^ Similar methods have utilized additional metrics or correction factors.^48,62–64^ Despite some clear limitations, the accuracy suggests this high-throughput computational approach may have further utility in predicting enzyme–substrate interactions. To more rigorously establish this method, additional proteins will need to be similarly analyzed in future studies. Notably, experimental approaches, such as yeast-two-hybrid assays, can be prone to generating false positives.^65–68^ Perhaps a proteome-wide AlphaFold-enabled prediction will alleviate some of these issues by serving as a corroborating tool.

The present study also provides a case study in how the sequence conservation of an enzyme sequence is influenced by the extent to which a gene-encoded substrate diverges. For instance, YcaO enzymes commonly modify RiPP precursor peptides, which tend to rapidly diverge even within the same RiPP class. To maintain effective substrate processing, YcaO enzymes have likewise diverged by acquiring numerous compensatory mutations or off-loading the task of maintaining substrate specificity to a partner domain such as the RiPP Recognition Element.^55,57^ In contrast, the sequence of uL16 is under immense selective pressure to be preserved owing to its critical role in protein translation. Accordingly, YcaO enzymes that modify uL16 are not rapidly diverging, as they have evolved to modify a relatively static substrate. Data to support these evolutionary claims are evident from our SSN analysis, with the uL16-modifying YcaO group having a high convergence ratio and RiPP-associated YcaO groups being considerably lower.

While this reports shows the successful *in vitro* biochemical reconstitution of *Ec*YcaO enzymatic activity, our reactions utilized a nonphysiological concentration of sulfide.^69^ While some thioamide-forming YcaO enzymes are encoded by organisms that reside in high-sulfide environments, those that do not tend to employ a thiocarboxylated protein carrier (homologues of ThiS) and TfuA hydrolase to efficiently transfer sulfide equivalents to their substrates.^70^ Importantly, *E. coli* do not encode a TfuA homologue, nor is TfuA encoded near any suspected uL16-modifying YcaO enzyme. Therefore, more investigation will be needed to determine how sulfide equivalents are delivered to the uL16 recipient protein. Given the prevalence of uL16-modifying YcaO enzymes in Pseudomonadota, we suspect that proteins from primary metabolic pathways may be co-opted for this purpose. Work is currently in progress to elucidate this sulfur transfer pathway.

Regarding the function of uL16 thioamidation, the proximity of Met82 to the PTC (Figure S38) suggests a putative role in translational rate and/or fidelity. Thioamides are often considered isosteric with amides, but the increased C = S bond length (∼1.66 A relative to ∼1.22 for C=O) and a more limited conformational flexibility must also be considered.^71^ Furthermore, the geometries in the high-resolution cryo-EM structure of the *E. coli* ribosome (PDB: 7K00) appear consistent with the formation of a hydrogen bond (H-bond) between the β-hydroxyl group of Arg81 and Met82 of *Ec*uL16. While thioamidation makes Met82 a weaker H-bond acceptor, it may cause unforeseen issues when the thioamide is present without the nearby β-hydroxy group. Notably, when observing the electrostatic potential maps of amides versus thioamides, a “bald spot” of electron density is observed along the C=S bond.^72^ This influences the ideal angle with which H-bonds may form, shifting them by upward of 16°.^71^ We also cannot rule out effects from potential H-bonding of the Gly83 amide N–H, which is a stronger H-bond donor upon thioamidation of Met82. Piecing together the precise role of these modifications in ribosome function will require significant further assessment.

## Conclusion

We have demonstrated through multiple lines of evidence that *Ec*YcaO is responsible for *Ec*uL16 thioamidation. Unlike previously characterized YcaO substrates, such as MCR or RiPP precursor peptides, modification of *Ec*uL16 requires recognition of a broad tertiary surface rather than a linear motif or leader peptide. Our combination of biochemical, genetic, and computational approaches enabled the identification of related YcaO enzymes from two Gram-negative ESKAPE pathogens that similarly modify their uL16 proteins.

As numerous YcaO homologues are encoded outside of RiPP biosynthetic gene clusters and lack obvious precursor peptides, our findings raise the possibility that additional members of this enzyme family may natively act on structured proteins. Demonstrating that several Pseudomonadota YcaO enzymes modify uL16 therefore broadens the established scope of YcaO activity and points toward a more diverse set of biological functions than previously recognized.

## Supplementary Material





## Notes

Accession identifiers of proteins mentioned in this study; primers used in this study; AlphaFold3 models; additional mass spectrometry data; SDS-PAGE analyses; proteolytic digestions of *Ec*YcaO; fluorescence polarization data; putative RiPP biosynthetic gene clusters; and isoelectric point comparison of sequence similarity network groups (DOCX)
(1) ipTM scores for the *E. coli* proteome-wide scan using *Ec*YcaO as the query protein; (2) ipTM scores for the *E. coli* proteome-wide scan using *Ec*uL16 as the query protein; (3) genome neighborhood analysis of *Ec*YcaO SSN group members with neutral or basic isoelectric points (Supporting Data Sets 1–3) (XLSX)

## ⊥

Immunome Inc, Bothwell, Washington 98011, United States

## #

Department of Plant Pathology, University of California Davis, Davis, California 95616, United States
