# Supplementary material for: Backbone Thioamidation of a Ribosomal Subunit Protein in Pseudomonadota
Source: Biochemistry. 2026 Mar 12;65(7):1056–69. doi: 10.1021/acs.biochem.5c00829 (PMC13063428; doi:10.1021/acs.biochem.5c00829)
Supplement: Supplementary file 1 [file bi5c00829_si_001.docx]

*Supporting Information for:*

**Backbone thioamidation of a ribosomal subunit protein in Pseudomonadota**

Andrew J. Rice^1†^, Yanqing Xue^1†^, Andi Liu^2,3^, Sangeetha Ramesh^2,4^, Oyin A. Ashiru^2^, Salimat O. Sofela^1^, Douglas A. Mitchell^1,5*^

^1^Department of Biochemistry, Vanderbilt University School of Medicine—Basic Sciences. Nashville, TN 37232, USA.

^2^Department of Microbiology, University of Illinois at Urbana-Champaign. Urbana, IL 61801, USA.

^3^Current: Immunome, Inc. Bothwell, WA 98011, USA.

^4^Current: Department of Plant Pathology, University of California Davis, Davis, CA 95616, USA.

^5^Department of Chemistry, Vanderbilt University. Nashville, TN 37232, USA.

^†^ These authors contributed equally.

* Corresponding author: douglas.mitchell@vanderbilt.edu

**Table of Contents**

Table S1: Accession identifiers of proteins mentioned in this studyS4

Figure S1: Examples of select YcaO-catalyzed post-translational modificationsS5

Figure S2: RimO catalyzes methylthiolation of Asp88 in uS12S6

Figure S3: AlphaFold3 modeling of *Ec*YcaO with ATP, 2 x Mg^2+^, and all ribosomal proteins S7

Figure S4: AlphaFold3 modeling of *Ec*YcaO with all ribosomal subunit proteins S8

Figure S5: AlphaFold3 model for *Ec*YcaO and *Ec*uL29S9

Figure S6: AlphaFold3 model for *Ec*YcaO and *Ec*uS12S10

Figure S7: AlphaFold3 model for *Ec*YcaO and RimOS11

Figure S8: AlphaFold3 modeling of *Ec*YcaO and EcuL16 with ATP, 2 x Mg^2+^ S12

Table S2: Proteins which produced an ipTM score ≥ 0.6 with *Ec*YcaO S13

Table S3: Proteins modeled with 2 copies of *Ec*YcaO S14

Figure S9: AlphaFold3 all-by-one analysis using *Ec*uL16 S15

Figure S10: AlphaFold3 model for *Ec*uL16 and *Ec*PncC S16

Figure S11: AlphaFold3 model for *Ec*uL16 and *Ec*EbgC S17

Figure S12: AlphaFold3 model for *Ec*uL16 and *Ec*RoxA S18

Figure S13: MALDI-TOF-MS of ribosomal proteins isolated from various *E. coli* strains S19

Figure S14: HR-MS of *Ec*uL16 isolated from *E. coli* *∆ycaO* ribosomes S20

Figure S15: HR-MS/MS of *Ec*uL16 isolated from *E. coli* *∆ycaO* ribosomes S21

Table S4: Primers used in this studyS22

Figure S16: Coomassie blue-stained SDS-PAGE gels of proteins used in this study S24

Figure S17: MALDI-TOF-MS for *Ec*uL16 endoproteinase GluC fragment oxidation S25

Figure S18: MALDI-TOF-MS for *Ec*uL16-His_6_ overexpression with *Ec*YcaO or *Ec*RoxAS26

Figure S19: SDS-PAGE gel of His_6_-tagged *Ec*uL16 overexpression with *Ec*YcaO or *Ec*RoxA S27

Figure S20: In-gel trypsin digestion of *Ec*YcaOS28

Figure S21: In-gel trypsin digestion of *Ec*RoxA S29

Figure S22: MALDI-TOF-MS for *Ec*uL16 overexpressed with ATP-binding deficient *Ec*YcaOS30

Figure S23: MALDI-TOF-MS for *Ec*uL16 overexpressed in *E. coli* BL21 (DE3) deletion strains S31

Figure S24: MALDI-TOF-MS for *Ec*YcaO reacted with *Ec*uL16 11-mer and 21-mer peptides S32

Figure S25: MALDI-TOF-MS for *Ec*YcaO reacted with endoproteinase GluC-digested *Ec*uL16S33

Figure S26: Structural depiction of the loop region of *Ec*uL16S34

Figure S27: Competitive FP data for *Ec*uL16 loop region Ala variantsS35

Table S5: Expected fragments of *Ec*uL16 Ala variants when digested with trypsinS36

Figure S28: Trypsin fragments of modified EcuL16 Ala variantsS37

Table S6: Expected fragments of *Ec*uL16 Ala variants when digested with endoproteinase GluCS38

Figure S29: Endoproteinase GluC fragments of modified EcuL16 Ala variants S39

Figure S30: MBP-*Ec*YcaO Ala variants mapped onto AlphaFold3 structure S40

Figure S31: FP data for MBP-*Ec*YcaO Ala variants S42

Figure S32: Isoelectric point comparison of sequence similarity network groupsS43

Figure S33: Putative RiPP biosynthetic gene clustersS44

Figure S34: HR-MS/MS for thioamidated *Kp*uL16 S45

Figure S35: HR-MS/MS for thioamidated *Pa*uL16S46

Figure S36: Genome-wide co-occurrence of *ycaO* and *roxA* S47

Figure S37: Structural alignment of *Ec*YcaO with reported YcaO structuresS48

Figure S38: Met82 of *Ec*uL16 is oriented towards the peptidyl transferase center (PTC)S49

Supporting References S50

**Table S1: Accession identifiers of proteins mentioned in this study.** A table displaying the name of each protein, as well as the associated accession identified, is provided.

| **UniProt Identifier** | **Protein** | **Native Organism** |
| --- | --- | --- |
| P0ADY7 | *Ec*uL16 | *Escherichia coli* K12 |
| P75838 | *Ec*YcaO | *Escherichia coli* K12 |
| P27431 | *Ec*RoxA | *Escherichia coli* K12 |
| P0A6G3 | PncC | *Escherichia coli* K12 |
| P0AC73 | EbgC | *Escherichia coli* K12 |
| A6TEW5 | *Kp*uL16 | *Klebsiella pneumoniae* ATCC 700721 |
| A6T6Z8 | *Kp*YcaO | *Klebsiella pneumoniae* ATCC 700721 |
| Q9HWE2 | *Pa*uL16 | *Pseudomonas aeruginosa* PAO1 |
| Q9I2H8 | *Pa*YcaO | *Pseudomonas aeruginosa* PAO1 |

**Figure S1.** Examples of select YcaO-catalyzed post-translational modifications. YcaO enzymes can utilize a diverse array of biological nucleophiles to perform nucleophilic attack of the peptide backbone, forming several unique chemical structures. Shown from top to bottom are macrolactamidine formation, thiazoline formation, proteolysis, and thioamide formation. Nuc = nucleophile.

**
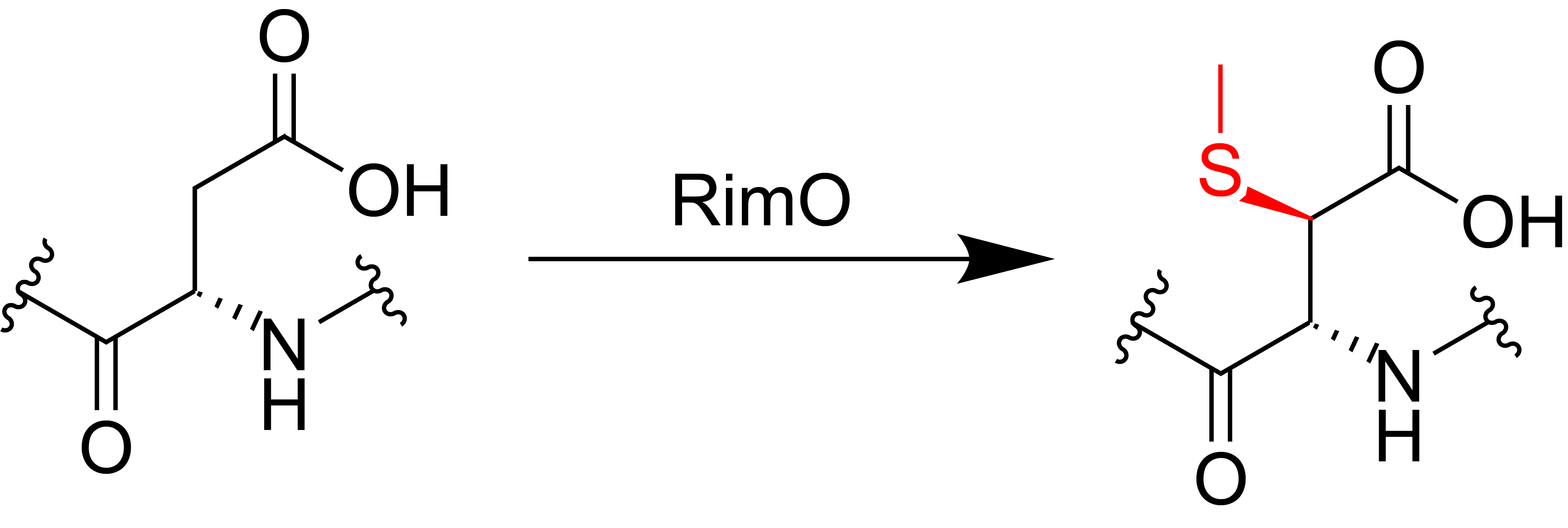
**

**Figure S2.** RimO catalyzes methylthiolation of Asp88 in uS12. RimO methylthiolates the β-carbon of the universally conserved Asp88 residue in uS12.^1–3^


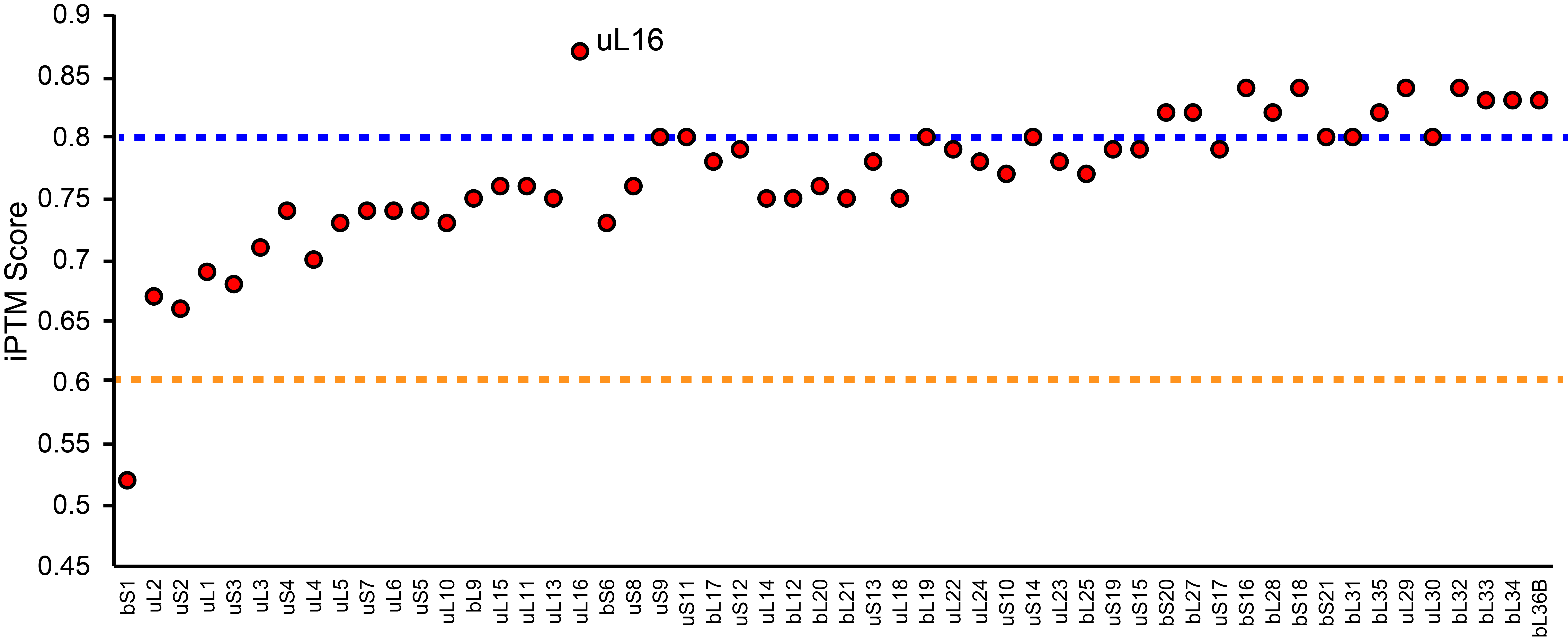


**Figure S3.** AlphaFold3 modeling of *Ec*YcaO with ATP, 2 x Mg^2+^ against all ribosomal proteins. A strong correlation is noted where smaller proteins produce a higher ipTM score, with *Ec*uL16 being an outlier. The confidence with which AlphaFold3 places the cofactors included here obscures the confidence for predicting protein-protein interactions with *Ec*YcaO and the individual ribosomal proteins.

**
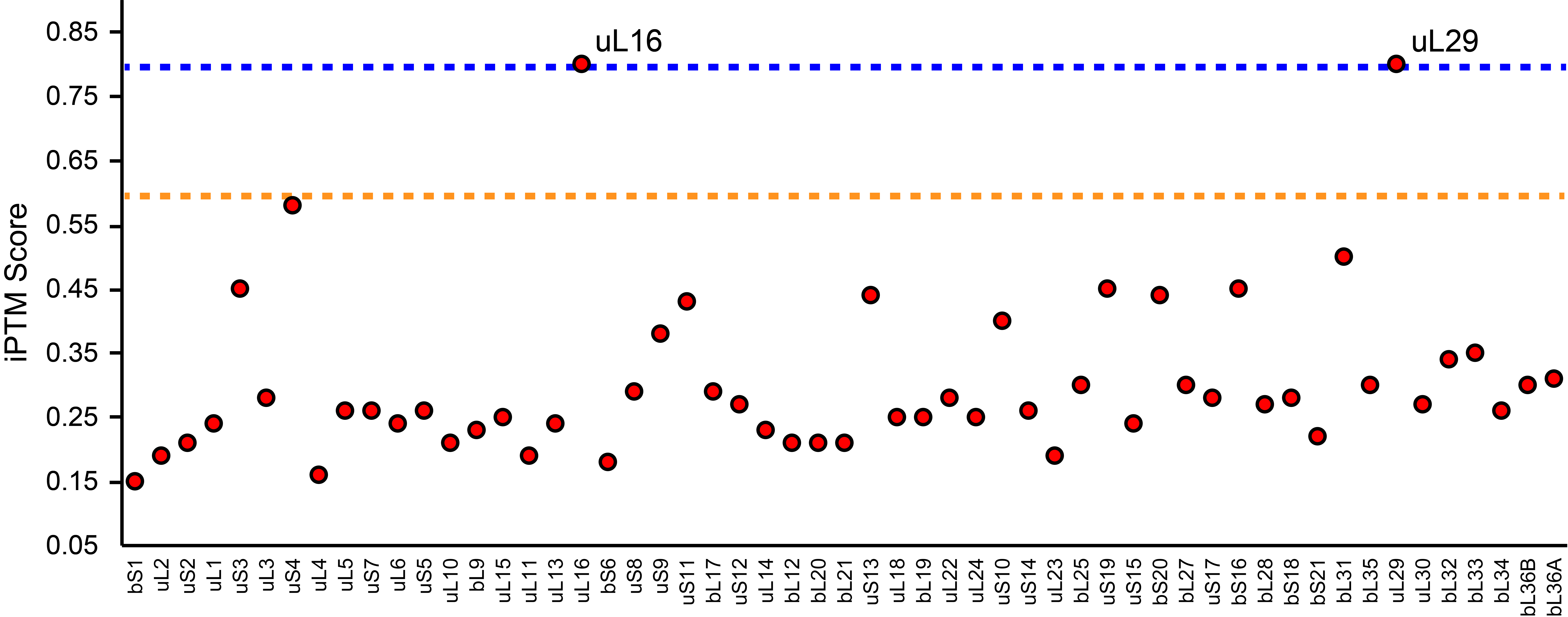
**

**Figure S4.** AlphaFold3 modeling of *Ec*YcaO with all ribosomal subunit proteins. Both *Ec*uL16, a known substrate of *Ec*YcaO, and *Ec*uL29, a previously identified interactor of *Ec*YcaO^4^, score confidently, while all other models fall below the threshold for a plausible structure. In contrast to Fig. S3, exclusion of cofactors results in higher confidence results since no trend between molecular weight and ipTM score was observed.

**
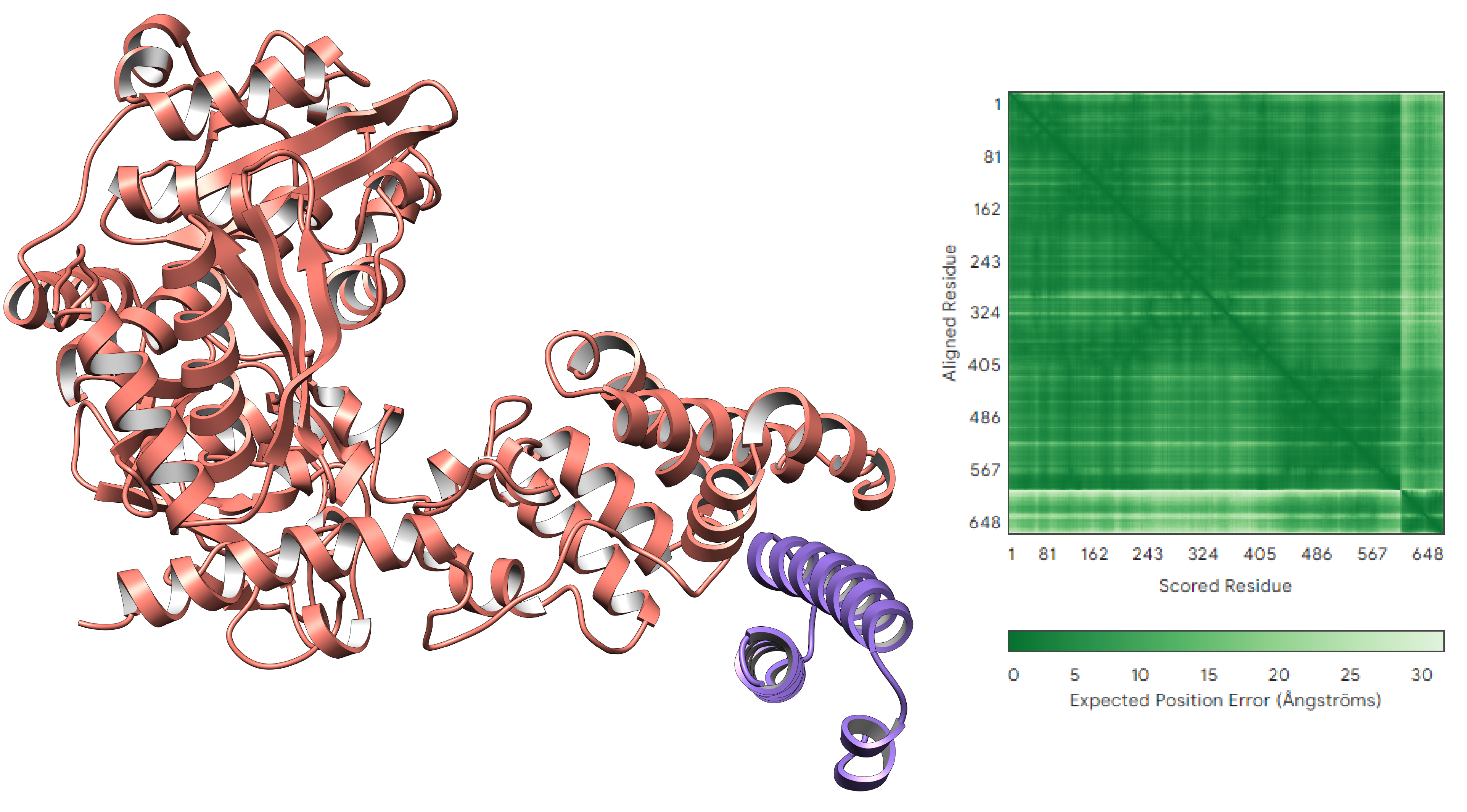
**

**Figure S5.** AlphaFold3 model for *Ec*YcaO and *Ec*uL29. *Ec*YcaO is depicted in salmon, and *Ec*uL29 is depicted in purple. The ipTM score for this model is 0.84 and the PAE plot for expected position error is on the right. The protein structural image was generated using Chimera.^5^

**
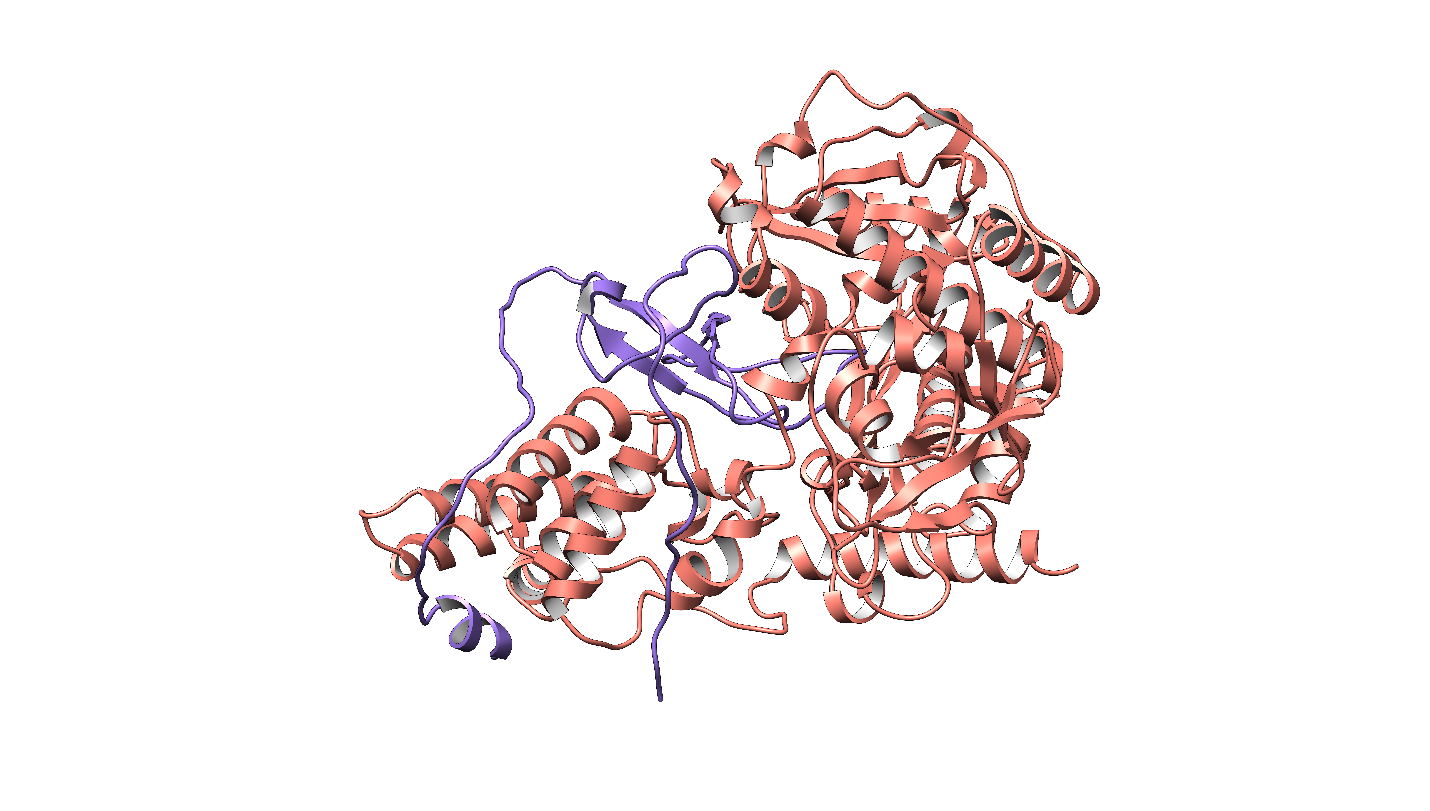

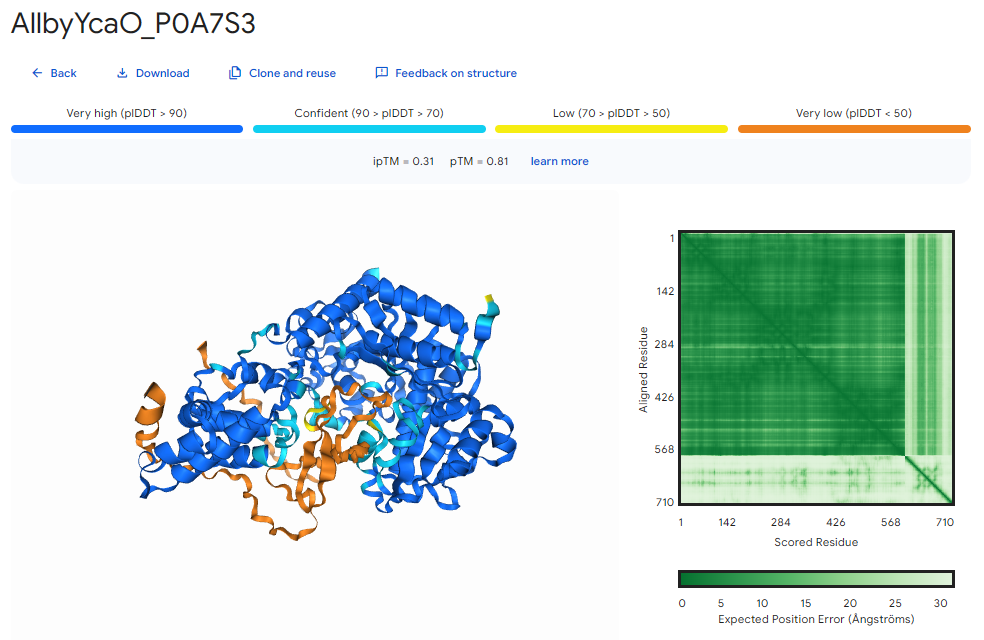
**

**Figure S6.** AlphaFold3 model for *Ec*YcaO and *Ec*uS12. *Ec*YcaO is depicted in salmon, and *Ec*uS12 is depicted in purple. The ipTM score for this model is 0.31 and the PAE plot for expected position error is on the right. The protein structural image was generated using Chimera.^5^

**
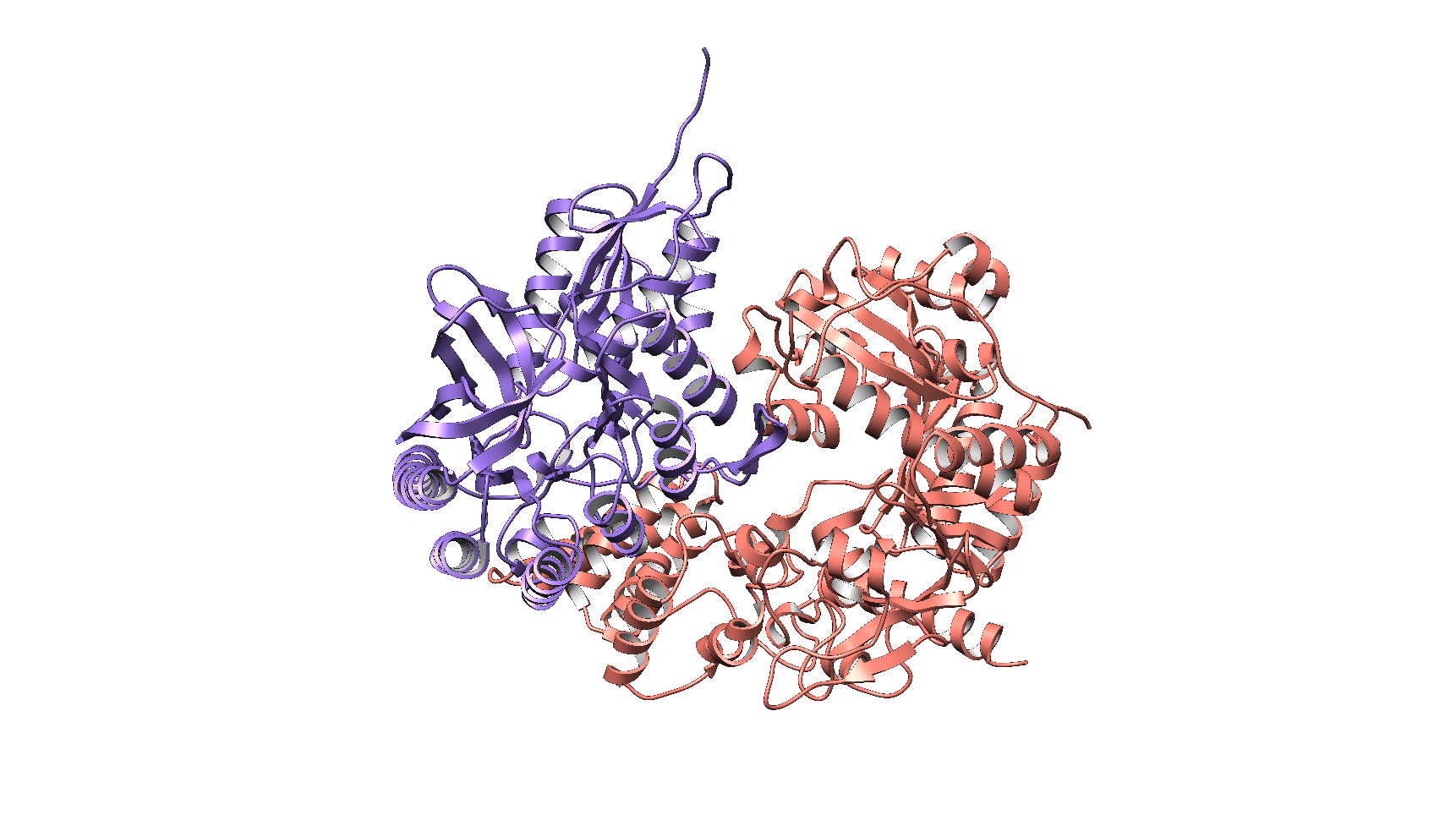

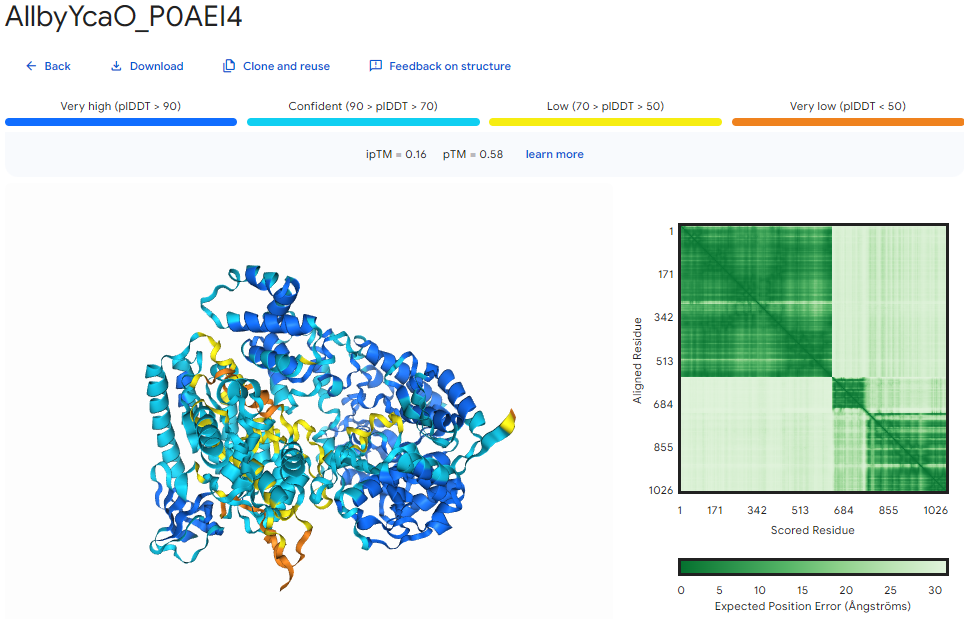
**

**Figure S7.** AlphaFold3 model for *Ec*YcaO and RimO. *Ec*YcaO is depicted in salmon, and *Ec*RimO is depicted in purple. The ipTM score for this model is 0.16, and the PAE plot for expected position error is on the right. The protein structural image was generated using Chimera.^5^


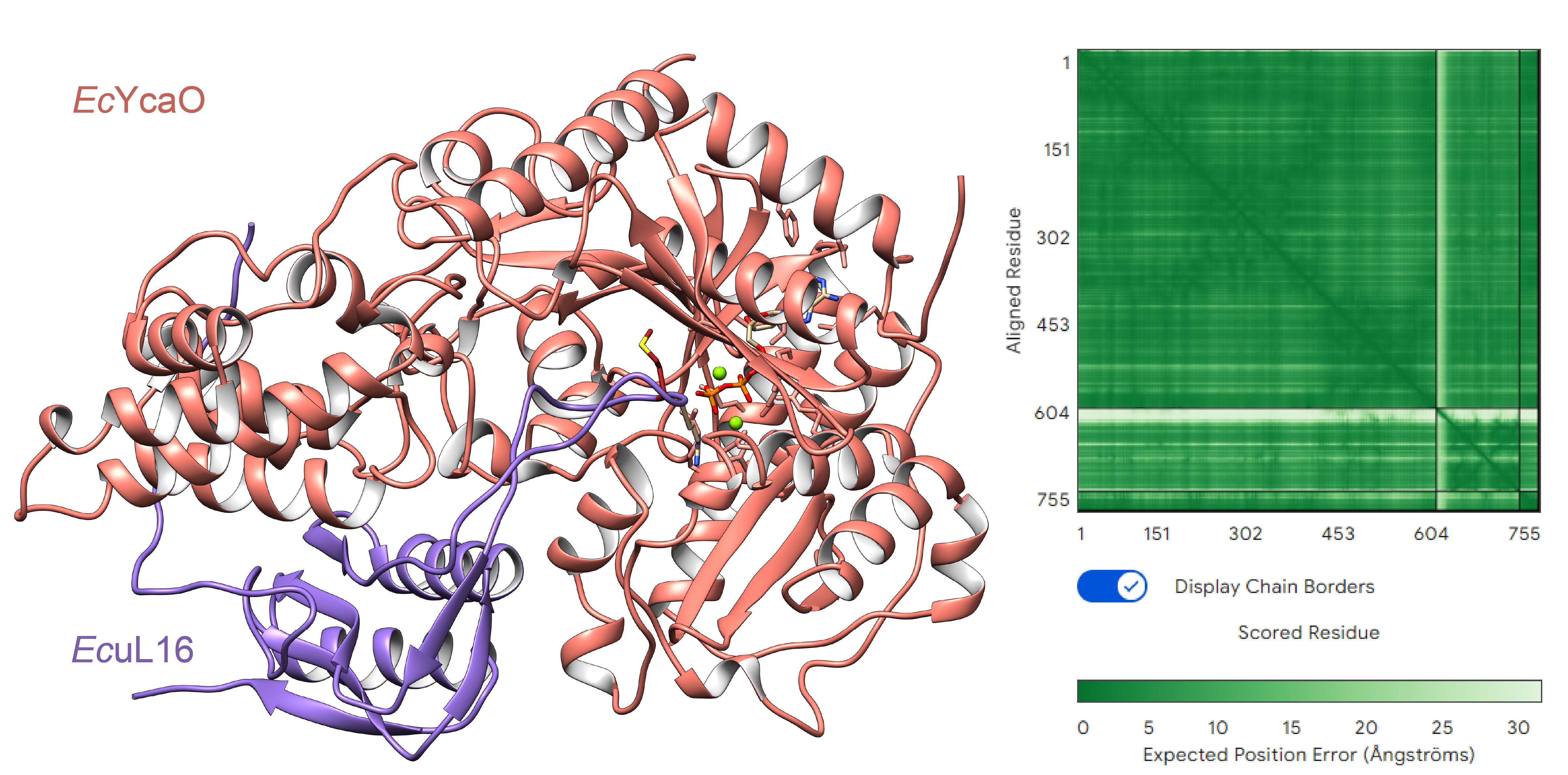


**Figure S8.** AlphaFold3 modeling of *Ec*YcaO and EcuL16 with ATP, 2 x Mg^2+^. *Ec*YcaO is depicted in salmon, and *Ec*uL16 is depicted in purple. The ipTM score for this model is 0.87, and the PAE plot for expected position error is on the right. The protein structural image was generated using Chimera.^5^

**Table S2.** Proteins producing an ipTM score ≥ 0.6 with *Ec*YcaO.

| **Uniprot ID** | **Protein** | **ipTM Score** |
| --- | --- | --- |
| P75838 | YCAO_ECOLI Ribosomal protein S12 methylthiotransferase accessory factor YcaO | 0.88 |
| P0A7M6 | RL29_ECOLI Large ribosomal subunit protein uL29 | 0.84 |
| P0ADY7 | RL16_ECOLI Large ribosomal subunit protein uL16 | 0.81 |
| P25746 | HFLD_ECOLI High frequency lysogenization protein HflD | 0.74 |
| P0DSH2 | BAXL_ECOLI Putative translational regulatory protein BaxL | 0.74 |
| A0A385XK32 | A0A385XK32_ECOLI Putative YjfA | 0.73 |
| P0AFI5 | PBP7_ECOLI D-alanyl-D-alanine endopeptidase | 0.72 |
| P0AE67 | CHEY_ECOLI Chemotaxis protein CheY | 0.71 |
| P77427 | YBEU_ECOLI Uncharacterized protein YbeU | 0.69 |
| Q47272 | YLCG_ECOLI Uncharacterized protein YlcG | 0.68 |
| P37750 | WBBJ_ECOLI Putative lipopolysaccharide biosynthesis O-acetyl transferase WbbJ | 0.68 |
| P15081 | GUTM_ECOLI Glucitol operon activator protein | 0.66 |
| P77731 | ALLA_ECOLI Ureidoglycolate lyase | 0.66 |
| P76194 | SUFE_ECOLI Cysteine desulfuration protein SufE | 0.65 |
| P60720 | LIPB_ECOLI Octanoyltransferase | 0.65 |
| Q47129 | FEAR_ECOLI Transcriptional activator FeaR | 0.64 |
| P30864 | YAFC_ECOLI Uncharacterized HTH-type transcriptional regulator YafC | 0.64 |
| P39409 | YJJW_ECOLI Putative glycyl-radical enzyme activating enzyme YjjW | 0.63 |
| P76541 | EUTL_ECOLI Bacterial microcompartment shell protein EutL | 0.62 |
| A5A621 | YPFM_ECOLI Uncharacterized protein YpfM | 0.62 |
| P0ACT2 | ENVR_ECOLI Probable acrEF/envCD operon repressor | 0.62 |
| P17443 | MURG_ECOLI UDP-N-acetylglucosamine--N-acetylmuramyl-(pentapeptide) pyrophosphoryl-undecaprenol N-acetylglucosamine transferase | 0.61 |
| P0A780 | NUSB_ECOLI Transcription antitermination protein NusB | 0.61 |
| P0AC65 | NRDH_ECOLI Glutaredoxin-like protein NrdH | 0.61 |
| P77667 | SUFA_ECOLI Iron-sulfur cluster assembly protein SufA | 0.61 |
| P15032 | RECE_ECOLI Exodeoxyribonuclease 8 | 0.60 |
| P0DSE9 | YCHT_ECOLI Protein YchT | 0.60 |

**Table S3.** Proteins modeled with dimeric *Ec*YcaO. For proteins-protein interactions with an ipTM score of 0.6 or higher, we generated an additional model using two copies of *Ec*YcaO. The specific ipTM score of our output is listed to the right of each protein.

| **Uniprot ID** | **Protein** | **ipTM Score** | **2x *Ec*YcaO ipTM Score** |
| --- | --- | --- | --- |
| P75838 | YCAO_ECOLI Ribosomal protein S12 methylthiotransferase accessory factor YcaO | 0.88 | 0.45 |
| P0A7M6 | RL29_ECOLI Large ribosomal subunit protein uL29 | 0.84 | 0.88 |
| P0ADY7 | RL16_ECOLI Large ribosomal subunit protein uL16 | 0.81 | 0.79 |
| P25746 | HFLD_ECOLI High frequency lysogenization protein HflD | 0.74 | 0.83 |
| P0DSH2 | BAXL_ECOLI Putative translational regulatory protein BaxL | 0.74 | 0.9 |
| A0A385XK32 | A0A385XK32_ECOLI Putative YjfA | 0.73 | 0.83 |
| P0AFI5 | PBP7_ECOLI D-alanyl-D-alanine endopeptidase | 0.72 | 0.61 |
| P0AE67 | CHEY_ECOLI Chemotaxis protein CheY | 0.71 | 0.86 |
| P77427 | YBEU_ECOLI Uncharacterized protein YbeU | 0.69 | 0.56 |
| Q47272 | YLCG_ECOLI Uncharacterized protein YlcG | 0.68 | 0.82 |
| P37750 | WBBJ_ECOLI Putative lipopolysaccharide biosynthesis O-acetyl transferase WbbJ | 0.68 | 0.78 |
| P15081 | GUTM_ECOLI Glucitol operon activator protein | 0.66 | 0.74 |
| P77731 | ALLA_ECOLI Ureidoglycolate lyase | 0.66 | 0.66 |
| P76194 | SUFE_ECOLI Cysteine desulfuration protein SufE | 0.65 | 0.85 |
| P60720 | LIPB_ECOLI Octanoyltransferase | 0.65 | 0.85 |
| Q47129 | FEAR_ECOLI Transcriptional activator FeaR | 0.64 | 0.61 |
| P30864 | YAFC_ECOLI Uncharacterized HTH-type transcriptional regulator YafC | 0.64 | 0.61 |
| P39409 | YJJW_ECOLI Putative glycyl-radical enzyme activating enzyme YjjW | 0.63 | 0.8 |
| P76541 | EUTL_ECOLI Bacterial microcompartment shell protein EutL | 0.62 | 0.63 |
| A5A621 | YPFM_ECOLI Uncharacterized protein YpfM | 0.62 | 0.88 |
| P0ACT2 | ENVR_ECOLI Probable acrEF/envCD operon repressor | 0.62 | 0.77 |
| P17443 | MURG_ECOLI UDP-N-acetylglucosamine--N-acetylmuramyl-(pentapeptide) pyrophosphoryl-undecaprenol N-acetylglucosamine transferase | 0.61 | 0.75 |
| P0A780 | NUSB_ECOLI Transcription antitermination protein NusB | 0.61 | 0.69 |
| P0AC65 | NRDH_ECOLI Glutaredoxin-like protein NrdH | 0.61 | 0.78 |
| P77667 | SUFA_ECOLI Iron-sulfur cluster assembly protein SufA | 0.61 | 0.72 |
| P15032 | RECE_ECOLI Exodeoxyribonuclease 8 | 0.6 | 0.46 |
| P0DSE9 | YCHT_ECOLI Protein YchT | 0.6 | 0.9 |


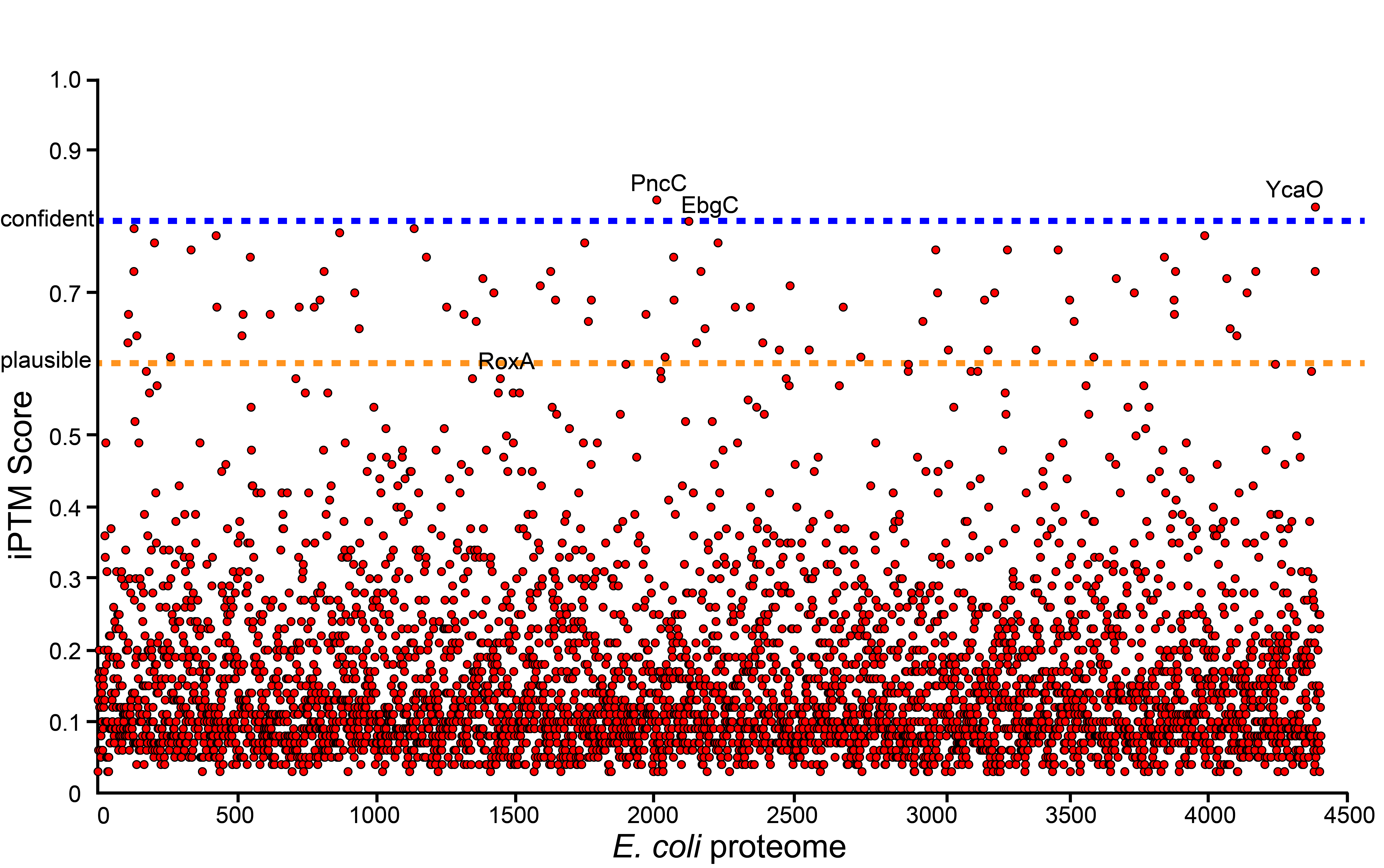


**Figure S9.** AlphaFold3 all-by-one analysis using *Ec*uL16. *Ec*uL16 was modeled in combination with every protein in the *E. coli* proteome (*n* = 4,403). Each red circle corresponds to a protein’s ipTM score. Proteins with an ipTM score ≥0.8, along with RoxA, are labeled.


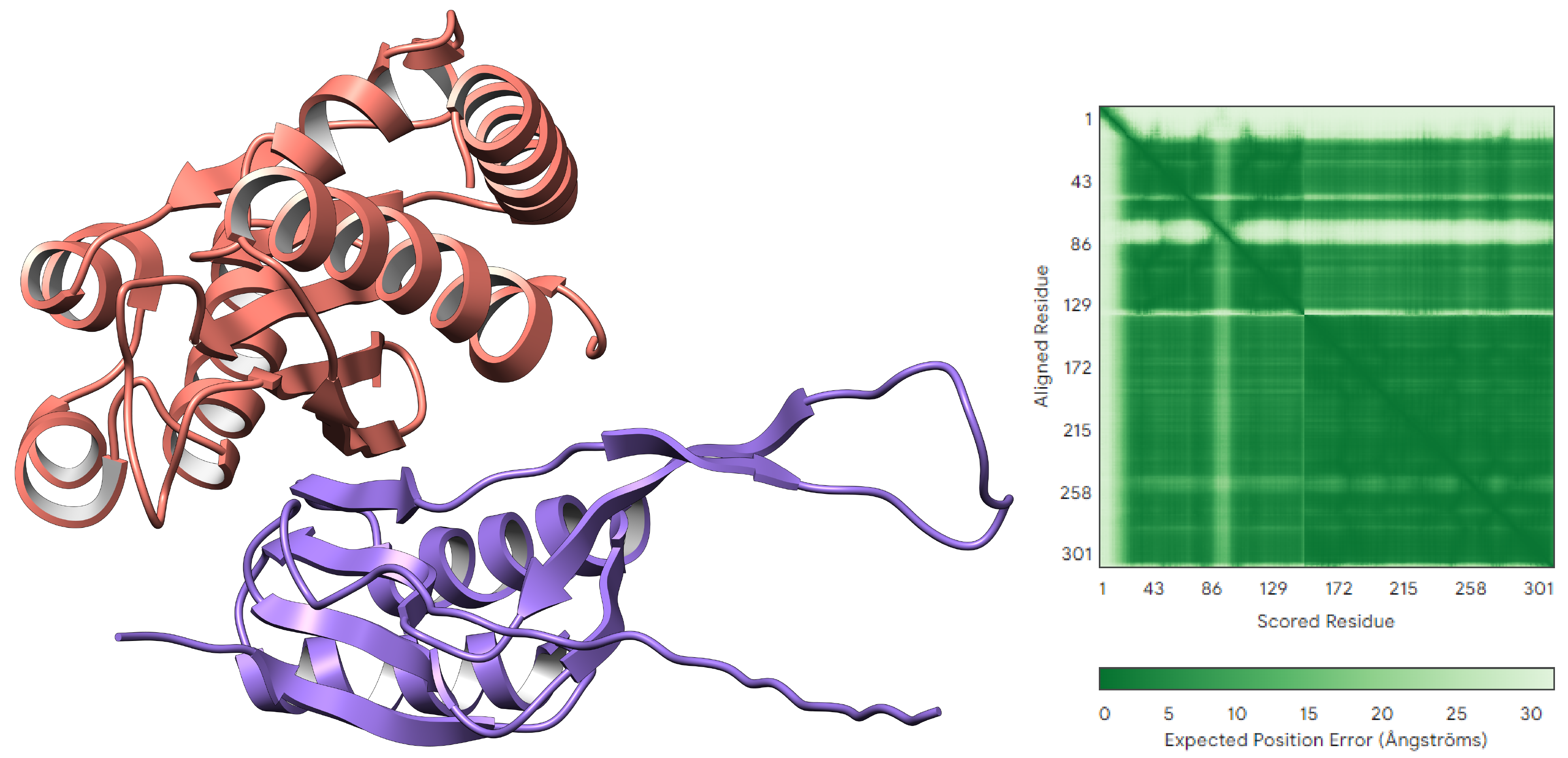


**Figure S10.** AlphaFold3 model for *Ec*uL16 and *Ec*PncC. *Ec*uL16 is depicted in purple, and *Ec*PncC is depicted in salmon. The ipTM score for this model is 0.82, and the PAE plot for expected position error is on the right. The protein structural image was generated using Chimera.^5^


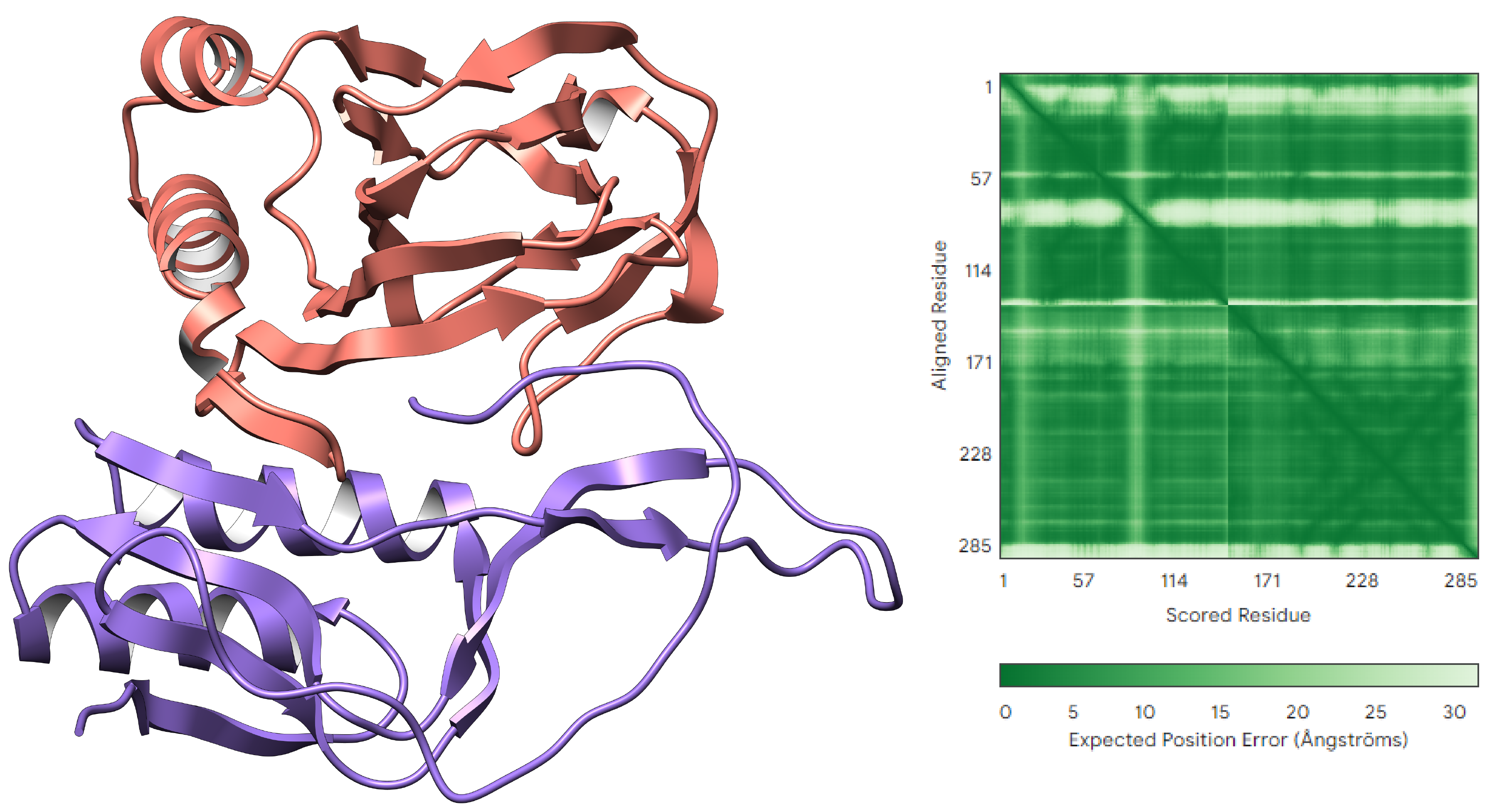


**Figure S11.** AlphaFold3 model for *Ec*uL16 and *Ec*EbgC. *Ec*uL16 is depicted in purple, and *Ec*EbgC is depicted in salmon. The ipTM score for this model is 0.80, and the PAE plot for expected position error is on the right. The protein structural image was generated using Chimera.^5^


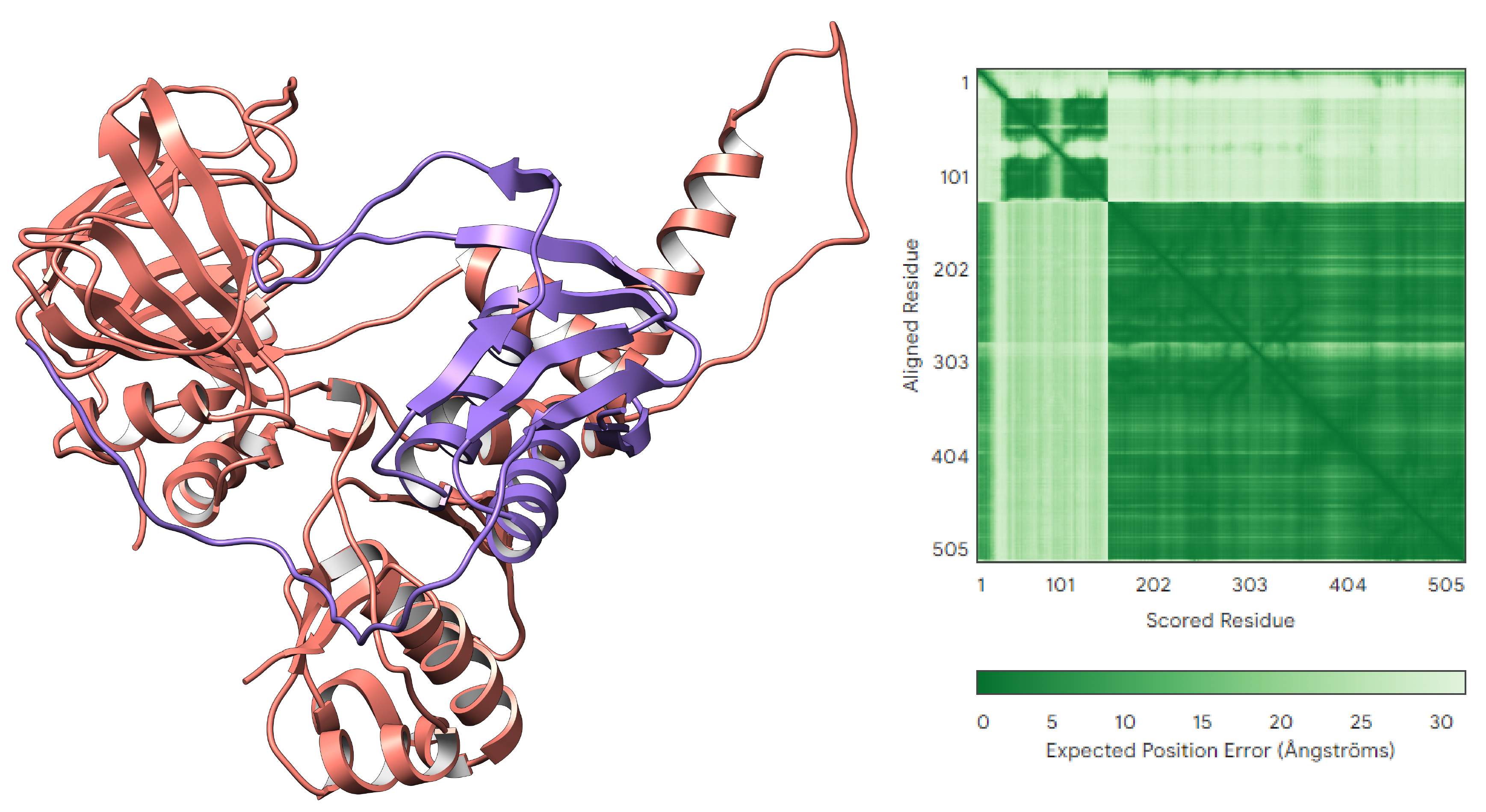


**Figure S12.** AlphaFold3 model for *Ec*uL16 and *Ec*RoxA. *Ec*uL16 is depicted in purple, and *Ec*RoxA is depicted in salmon. The ipTM score for this model is 0.57 (plausible range), and the PAE plot for expected position error is shown on the right. The loop of uL16 that contains the Arg residue modified by RoxA is placed in a catalytically competent location. The protein structural image was generated using Chimera.^5^

**
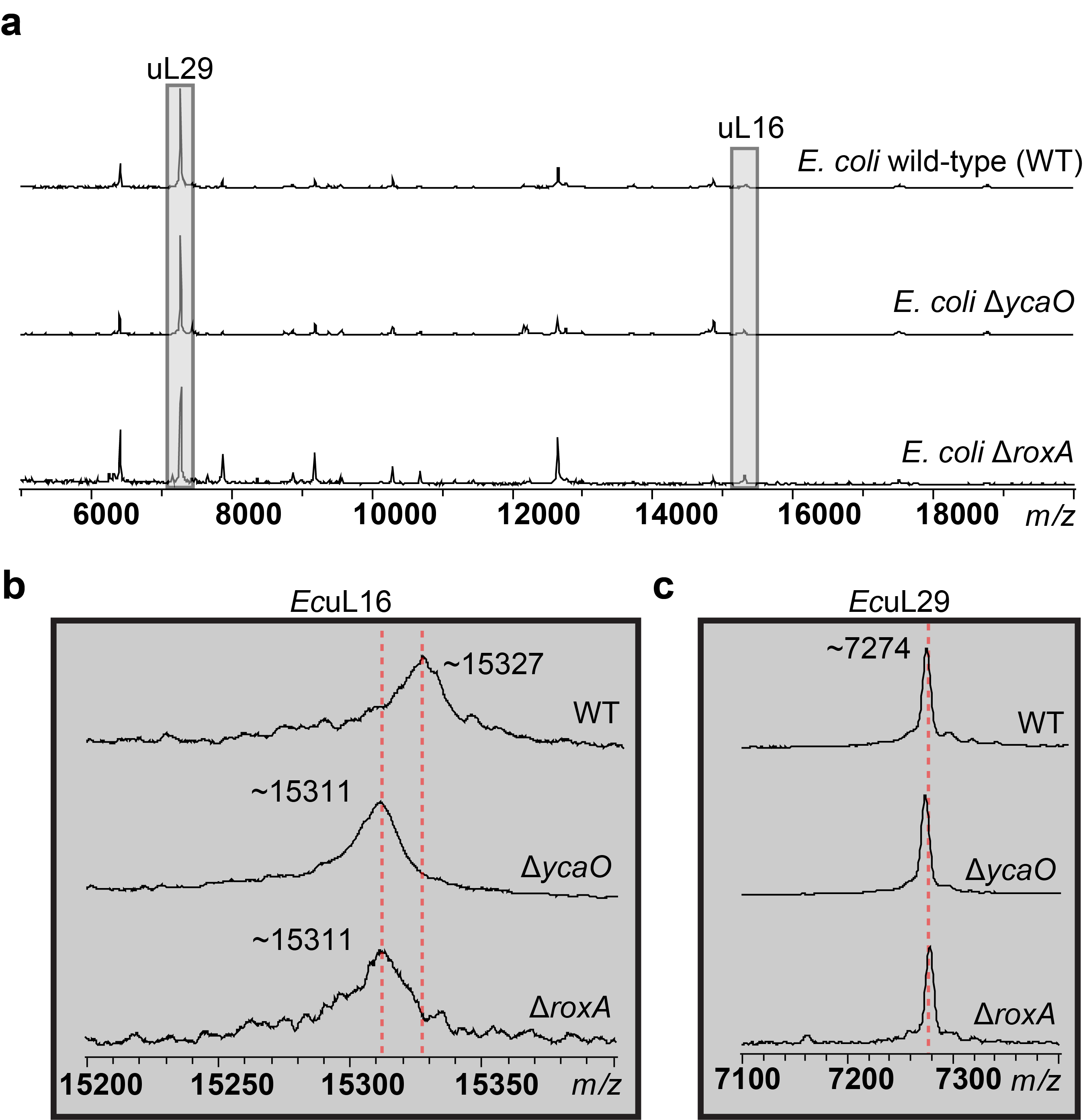
**

**Figure S13.** MALDI-TOF-MS of ribosomal proteins isolated from various *E. coli* strains. (a) MALDI-TOF MS analysis of ribosomal proteins purified from WT, *ΔycaO*, and *ΔroxA*. The regions near the peaks corresponding to uL16 and uL29 are highlighted and enlarged in panels (b) and (c), respectively. The m/z shift between uL16 obtained from WT is ~16 Da heavier than that obtained from the *ΔycaO* and *ΔroxA* strains. Meanwhile, the observed m/z of uL29 was consistent across the three samples

**
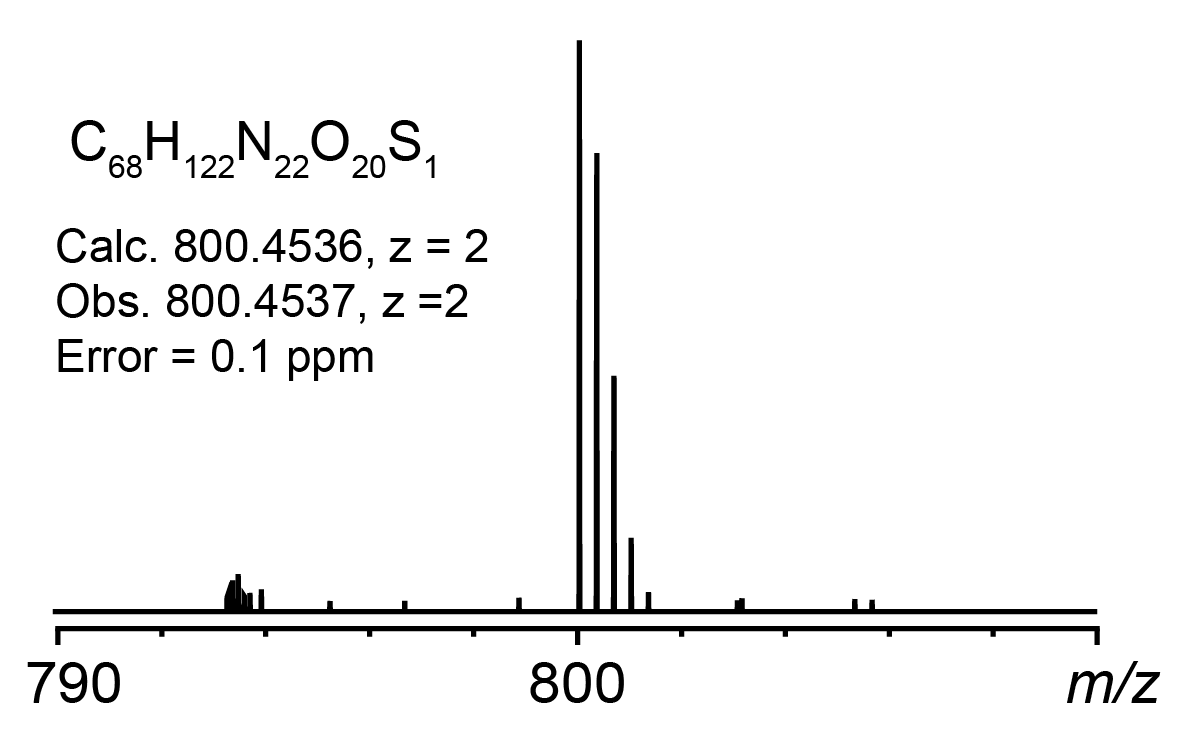
**

**Figure S14.** HR-MS of *Ec*uL16 isolated from *E. coli* *∆ycaO* ribosomes. *Ec*uL16 was isolated from crude *E. coli* *∆ycaO* ribosomes by SDS-PAGE, in-gel digested with endoproteinase GluC, and analyzed by HR-MS/MS as described in the Methods. The ppm error for the loss of thioamidation (0.1 ppm) vs that of oxygen loss (11.2 ppm) supports a lack of thioamidation as responsible for the 16 Da mass loss in this peptide. R(O) represents the β-hydroxylation of Arg catalyzed by RoxA. Figure S15 shows the tandem MS analysis for this peptide.


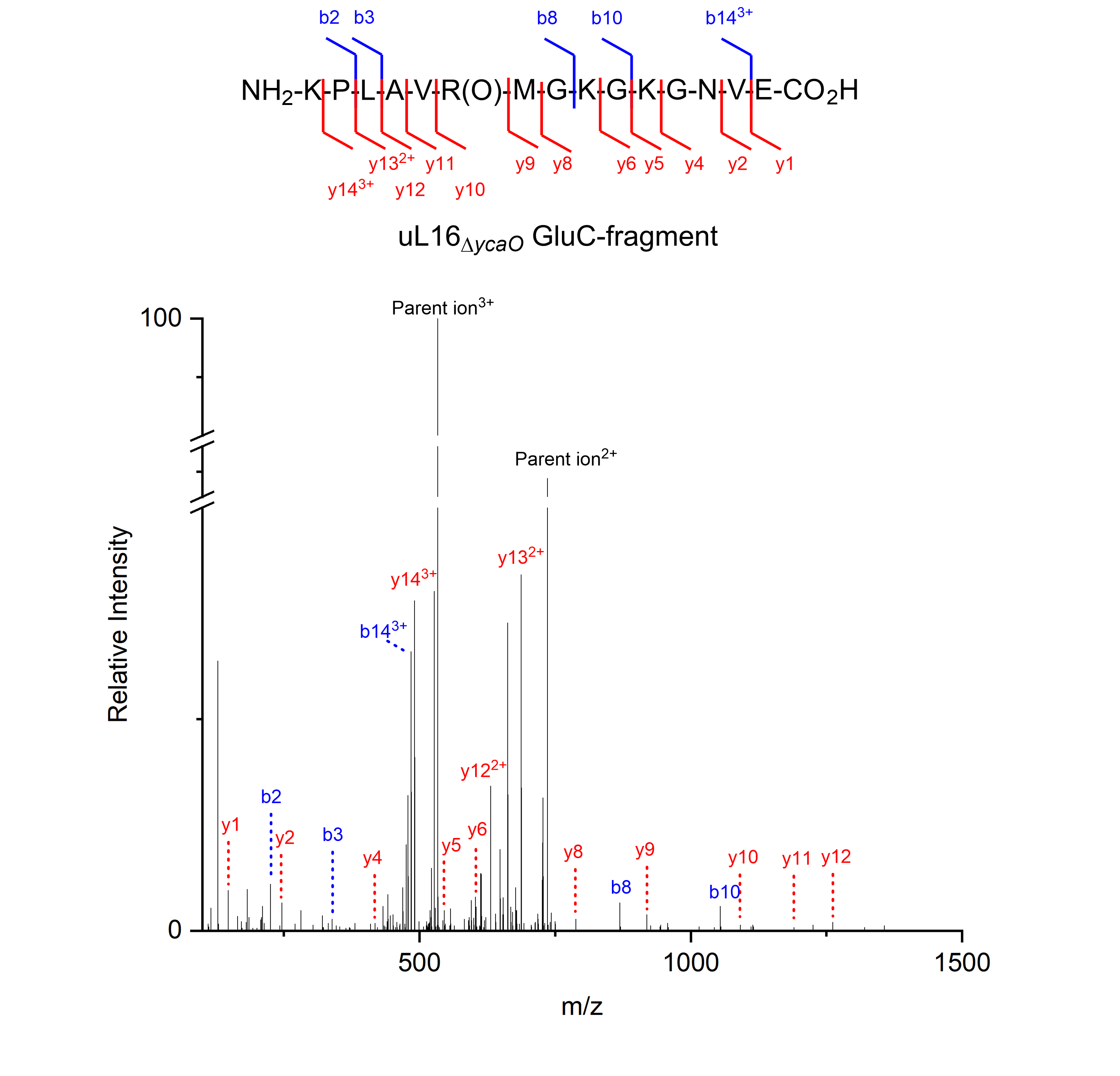


**Figure S15.** HR-MS/MS of *Ec*uL16 isolated from *E. coli* *∆ycaO* ribosomes. *Ec*uL16 was isolated from crude *E. coli* *∆ycaO* ribosomes by SDS-PAGE, in-gel digested with endoproteinase GluC, and analyzed by HR-MS/MS as described in the Methods.

**Table S4**. Primers used in this study. The name, purpose, and sequence of each primer is provided.

| **Name** | **Purpose** | **Sequence (5’ to 3’)** |
| --- | --- | --- |
| ycaO.F1 | Deletion forward primer for BL21(DE3) Δ*ycaO* | attattgcgcgctttccgtacgactaaagtgattttcatgacgcaaacatttatccccgg |
| ycaO.R1 | Deletion reverse primer for BL21(DE3) Δ*ycaO* | taatcattaagcctacattgcgtaggctatttgattttattttgcccagaatgctgctttggc |
| ycaO.F2 | Verification forward primer for BL21(DE3) Δ*ycaO* | atgacgcaaacatttatccccggc |
| ycaO.R2 | Verification reverse primer for BL21(DE3) Δ*ycaO* | ttattttgcccagaatgctgctttggc |
| roxA.F1 | Deletion forward primer for BL21(DE3) Δ*ycaO/*Δ*roxA* | atggaataccaactcactcttaactggcccgattttgtagcgaccgagtgagctagcta |
| roxA.R1 | Deletion reverse primer for BL21(DE3) Δ*ycaO/*Δ*roxA* | ttacccttcgaagaaccagtatccgctattgaccaggacattatttgccgactaccttgg |
| roxA.F2 | Verification forward primer for BL21(DE3) Δ*ycaO/*Δ*roxA* | atggaataccaactcactcttaactggccc |
| roxA.R2 | Verification reverse primer for BL21(DE3) Δ*ycaO/*Δ*roxA* | ttacccttcgaagaaccagtatccgc |
| roxA.F3 | Deletion forward primer for K-12 Δ*ycaO/*Δ*roxA* | atggaataccaactcactcttaactggcccgatttttgatcggcacgtaagaggtt |
| roxA.R3 | Deletion reverse primer for K-12 Δ*ycaO/*Δ*roxA* | ttacccttcgaagaaccaatacccgctattgaccagttacgccccgccctgccact |
| roxA.F4 | Verification forward primer for K-12 Δ*ycaO/*Δ*roxA* | atggaataccaactcactcttaactggcc |
| roxA.R4 | Verification reverse primer for K-12 Δ*ycaO/*Δ*roxA* | ttacccttcgaagaaccaatacccgc |
| KpuL16.F | Cloning of *Kp*uL16 | cacagccaggatccgaattcaatgttacaaccaaagcgtacaaaattcc |
| KpuL16.R | Cloning of *Kp*uL16 | gcattatgcggccgcaagcttttacatcaccgtcttagttacaaaggtgg |
| KpYcaO.F | Cloning of *Kp*YcaO | ttagtatattagttaagtataagaaggagatatacatatgacccaaacgtttattcccgg |
| KpYcaO.R | Cloning of *Kp*YcaO | gcggtttctttaccagactcgagttattttgcccaaaacgccgcttt |
| PauL16.F | Cloning of *Pa*uL16 | cacagccaggatccgaattcaatgctgcaacccaagcgtac |
| PauL16.R | Cloning of *Pa*uL16 | gcattatgcggccgcaagctttcacatcaccgtccgcttaac |
| PaYcaO.F | Cloning of *Pa*YcaO | tatattagttaagtataagaaggagatatacatatggtggaagtaaaggtcaattttctc |
| PaYcaO.R | Cloning of *Pa*YcaO | gtttctttaccagactcgagggtacctcacttgcgcttgcggatcg |
| uL16_P72A_F | *Ec*uL16 mutagenesis | cccggacaaagcgatcactgaaaagccgctggcagtgcgtatg |
| uL16_P72A_R | *Ec*uL16 mutagenesis | cttttcagtgatcgctttgtccgggaacacacggatccagatc |
| uL16_T74A_F | *Ec*uL16 mutagenesis | gacaaaccgatcgctgaaaagccgctggcagtgcgtatggg |
| uL16_T74A_R | *Ec*uL16 mutagenesis | gcggcttttcagcgatcggtttgtccgggaacacacggatc |
| uL16_L78A_F | *Ec*uL16 mutagenesis | cactgaaaagccggcggcagtgcgtatgggtaaaggtaaagg |
| uL16_L78A_R | *Ec*uL16 mutagenesis | catacgcactgccgccggcttttcagtgatcggtttgtccgg |
| uL16_R81A_F | *Ec*uL16 mutagenesis | cgctggcagtggctatgggtaaaggtaaaggtaacgtggag |
| uL16_R81A_R | *Ec*uL16 mutagenesis | cctttacccatagccactgccagcggcttttcagtgatcg |
| uL16_M82A_F | *Ec*uL16 mutagenesis | ggcagtgcgtgcgggtaaaggtaaaggtaacgtggagtattg |
| uL16_M82A_R | *Ec*uL16 mutagenesis | ctttacctttacccgcacgcactgccagcggcttttcagtgatc |
| uL16_G83A_F | *Ec*uL16 mutagenesis | cagtgcgtatggctaaaggtaaaggtaacgtggagtattgg |
| uL16_G83A_R | *Ec*uL16 mutagenesis | cctttacctttagccatacgcactgccagcggcttttcagtg |
| uL16_G85A_F | *Ec*uL16 mutagenesis | gtatgggtaaagctaaaggtaacgtggagtattgggttgcc |
| uL16_G85A_R | *Ec*uL16 mutagenesis | cgttacctttagctttacccatacgcactgccagcggcttttc |
| uL16_V89A_F | *Ec*uL16 mutagenesis | gtaaaggtaacgcggagtattgggttgccttgattcagccg |
| uL16_V89A_R | *Ec*uL16 mutagenesis | acccaatactccgcgttacctttacctttacccatacgcac |
| L16HCys_R | *Ec*uL16 mutagenesis | cttaagcattatttagcagtggtgatgatggtgatggctgctgccc |
| L16HCys_F | *Ec*uL16 mutagenesis | tcatcaccactgctaaataatgcttaagtcgaacagaaagtaatc |
| YcaO_Gib2F | Cloning of *Ec*YcaO | gccaaagcagcattctgggcaaaataagcagatctcaattggatatc |
| YcaO_Gib2R | Cloning of *Ec*YcaO | gatatccaattgagatctgcttattttgcccagaatgctgctttggc |
| YcaO_Gib1R | Cloning of *Ec*YcaO | tgccggggataaatgtttgcgtcatatgtatatctccttcttatac |
| YcaO_Gib1F | Cloning of *Ec*YcaO | gtataagaaggagatatacatatgacgcaaacatttatccccggca |
| EcYcaO_L55A_F | *Ec*YcaO mutagenesis | aagagtgcgcagcgtgttttaccaacggtaaaggcgcaacc |
| EcYcaO_L55A_R | *Ec*YcaO mutagenesis | ttggtaaaacacgctgcgcactctttgtcgcgaatatgtac |
| EcYcaO_F57A_F | *Ec*YcaO mutagenesis | gcgcactgtgtgctaccaacggtaaaggcgcaaccaagaaag |
| EcYcaO_F57A_R | *Ec*YcaO mutagenesis | accgttggtagcacacagtgcgcactctttgtcgcgaatatg |
| EcYcaO_R79A_F | *Ec*YcaO mutagenesis | gaatatttcgaggctctctcaaccaactacttttttgcggac |
| EcYcaO_R79A_R | *Ec*YcaO mutagenesis | ttggttgagagagcctcgaaatattcaccgagtgcagaagc |
| EcYcaO_D88A_F | *Ec*YcaO mutagenesis | acttttttgcggccttctggctgggcgaaaccatcgccaac |
| EcYcaO_D88A_R | *Ec*YcaO mutagenesis | cccagccagaaggccgcaaaaaagtagttggttgagagacg |
| EcYcaO_R295A_F | *Ec*YcaO mutagenesis | tgctgcaaggtgctggcctgaaagatttggatgtgtttact |
| EcYcaO_R295A_R | *Ec*YcaO mutagenesis | tctttcaggccagcaccttgcagcagctcggtcacggtacg |
| EcYcaO_T317A_F | *Ec*YcaO mutagenesis | tcgctgaacatgccaacctcgaaacgcactttatcgattcc |
| EcYcaO_T317A_R | *Ec*YcaO mutagenesis | gtttcgaggttggcatgttcagcgacttcttcatcatcgaa |
| EcYcaO_D325A_F | *Ec*YcaO mutagenesis | cgcactttatcgcttccagcggtttaatctcctgggacctg |
| EcYcaO_D325A_R | *Ec*YcaO mutagenesis | aaaccgctggaagcgataaagtgcgtttcgaggttggtatg |
| EcYcaO_L329A_F | *Ec*YcaO mutagenesis | attccagcggtgcaatctcctgggacctgttcaagcaggat |
| EcYcaO_L329A_R | *Ec*YcaO mutagenesis | tcccaggagattgcaccgctggaatcgataaagtgcgtttc |
| EcYcaO_R444A_F | *Ec*YcaO mutagenesis | atgactttaccgccgtgcgtgagctgttgggtctggcgacc |
| EcYcaO_R444A_R | *Ec*YcaO mutagenesis | agctcacgcacggcggtaaagtcatcaaaaccttcttcatc |
| EcYcaO_R463A_F | *Ec*YcaO mutagenesis | ggtacaccctggctatcggtgaattaaaagccatgctggcg |
| EcYcaO_R463A_R | *Ec*YcaO mutagenesis | aattcaccgatagccagggtgtaccaaccgttatccgaccc |
| EcYcaO_F491A_F | *Ec*YcaO mutagenesis | ggacgatggaggctaactcatcagtatttagtccggaacgc |
| EcYcaO_F491A_R | *Ec*YcaO mutagenesis | actgatgagttagcctccatcgtccattcggtccagaccag |
| RoxA_Gib1F | Cloning of *Ec*RoxA | gtataagaaggagatatacatatggaataccaactcactcttaactg |
| RoxA_Gib1R | Cloning of *Ec*RoxA | cagttaagagtgagttggtattccatatgtatatctccttcttatac |
| RoxA_Gib2F | Cloning of *Ec*RoxA | ggtattggttcttcgaagggtaagcagatctcaattggatatc |
| RoxA_Gib2R | Cloning of *Ec*RoxA | gatatccaattgagatctgcttacccttcgaagaaccaatacc |
| RoxA_MCS1_F1 | Cloning of *Ec*RoxA | ggatctgagaacctgtacttccaatccgaataccaactcactcttaactg |
| RoxA_MCS1_R1 | Cloning of *Ec*RoxA | cagttaagagtgagttggtattcggattggaagtacaggttctcagatcc |
| RoxA_MCS1_F2 | Cloning of *Ec*RoxA | ggtattggttcttcgaagggtaaggatccgaattcgtcgacaagctt |
| RoxA_MCS1_R2 | Cloning of *Ec*RoxA | aagcttgtcgacgaattcggatccttacccttcgaagaaccaatacc |

**
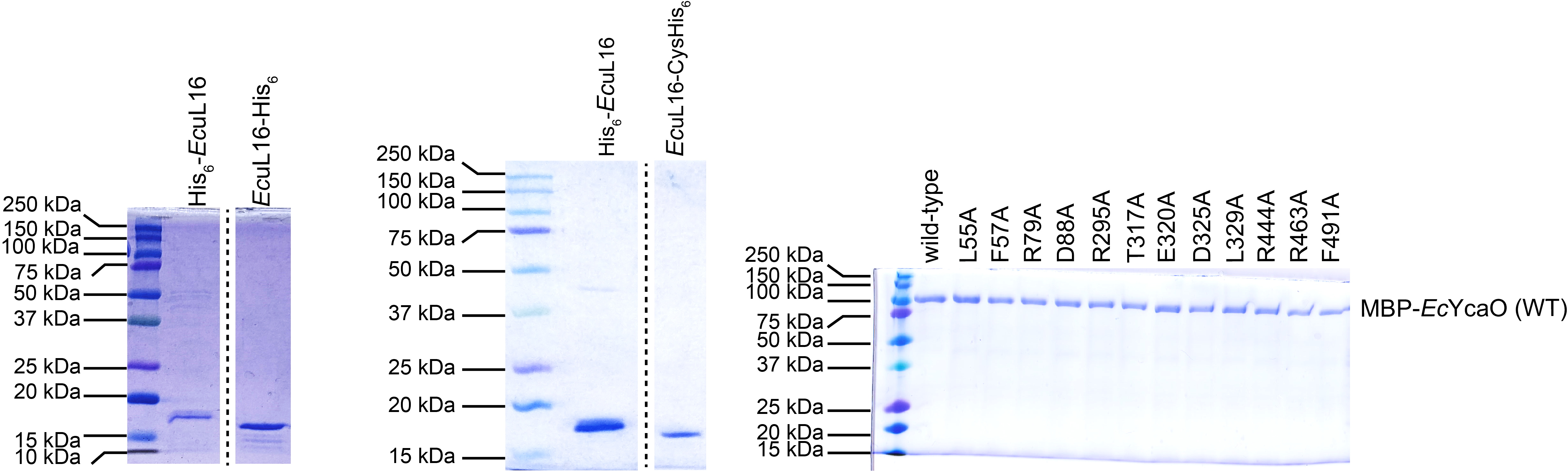
**

**Figure S16.** Coomassie-stained SDS-PAGE gels of proteins purified for this study.

**
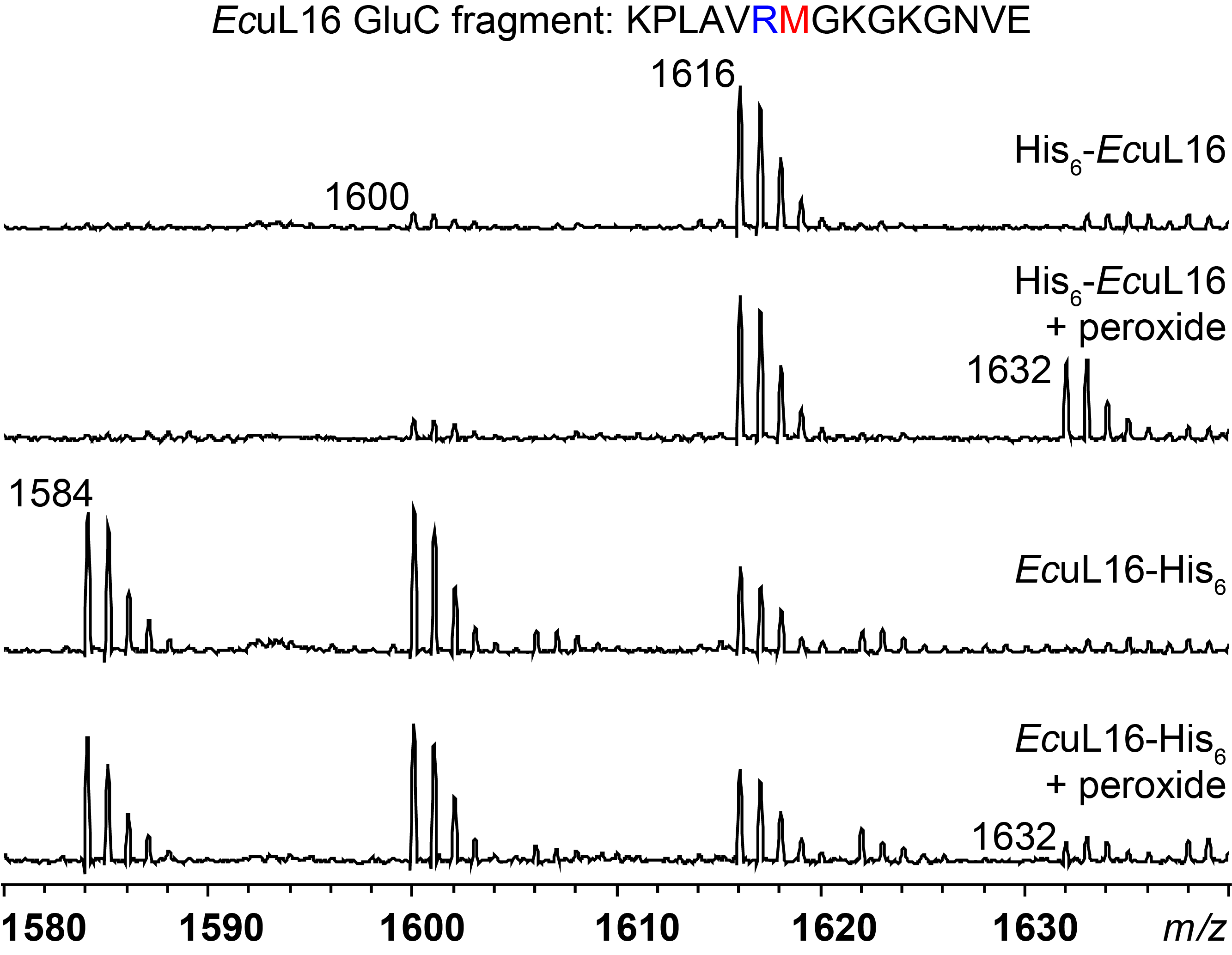
**

**Figure S17.** MALDI-TOF-MS for *Ec*uL16 endoproteinase GluC fragment oxidation. Oxidation of endoproteinase GluC fragments was achieved via a short incubation with hydrogen peroxide as described in the Materials & Methods section. The expected *m/z* for the unmodified endoproteinase GluC-fragment of *Ec*uL16 is 1584, 1600 for 1 x 16 Da modification (thioamidation, hydroxylation, or oxidation), 1616 for 2 x 16 Da modifications, and 1632 for 3 x 16 Da modifications.

**
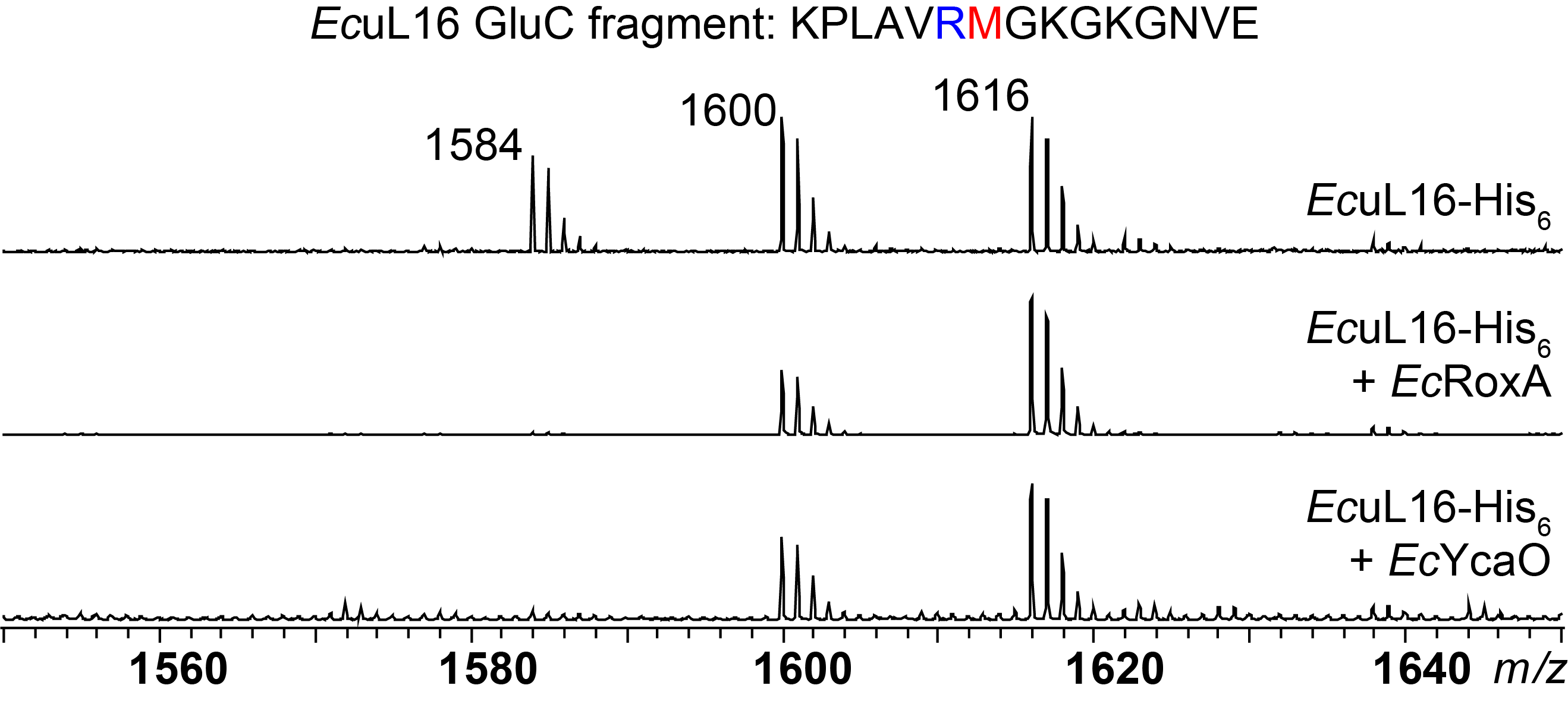
**

**Figure S18.** MALDI-TOF-MS for *Ec*uL16-His_6_ overexpression with *Ec*YcaO or *Ec*RoxA. *Ec*uL16-His_6_ was overexpressed with either *Ec*YcaO or *Ec*RoxA as described. A mass range inclusive of the endoproteinase GluC fragment containing the Arg81 and Met82 PTMs is shown. The expected m/z for the unmodified GluC-fragment of *Ec*uL16 is 1584, 1600 for 1 x 16 Da modification (thioamidation or hydroxylation), and 1616 for 2 x 16 Da modifications (thioamidation & hydroxylation).

**
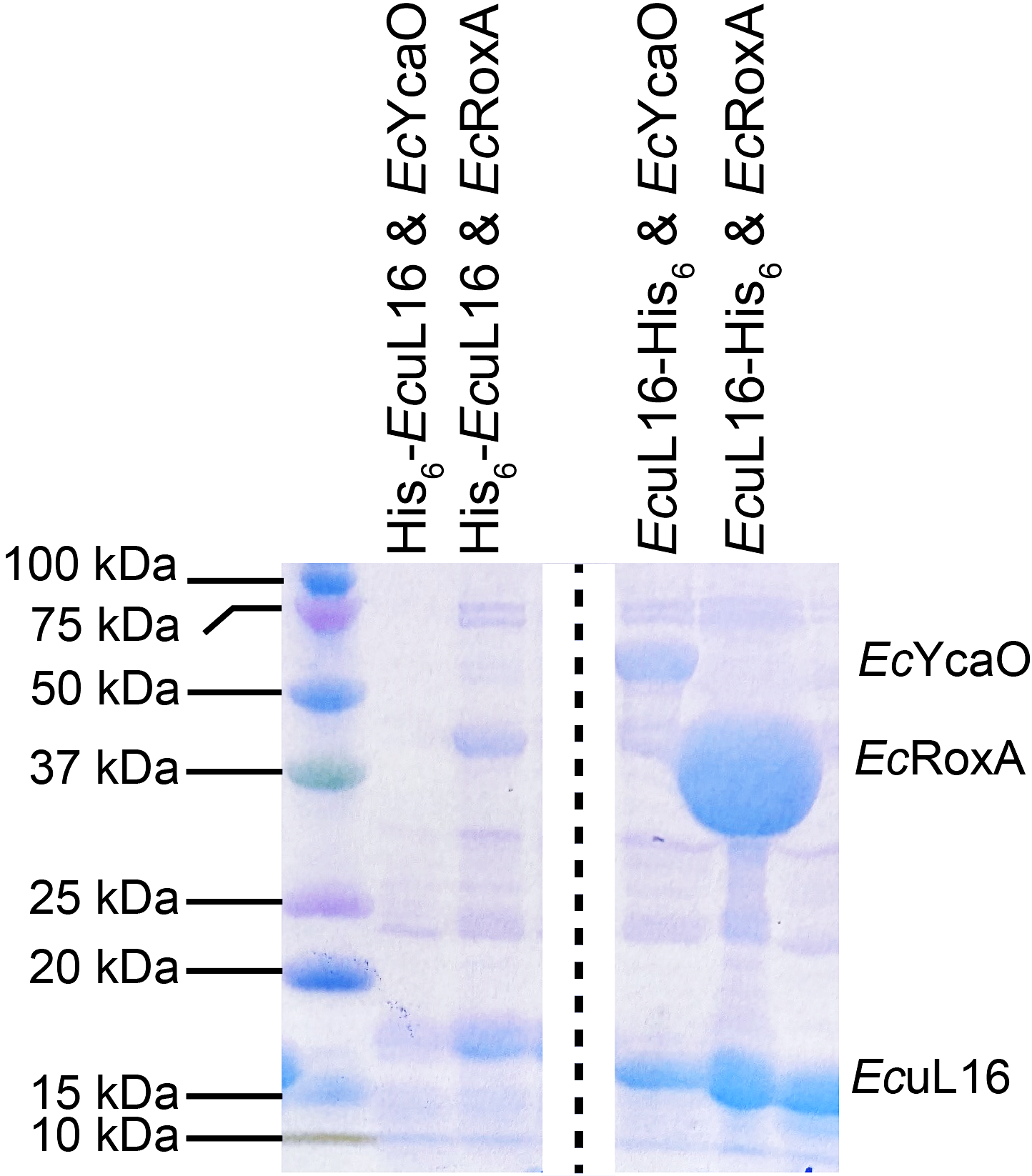
**

**Figure S19.** SDS-PAGE gel of His_6_-tagged *Ec*uL16 overexpression with *Ec*YcaO or *Ec*RoxA. His_6_-tagged *Ec*uL16 was overexpressed with either *Ec*YcaO or *Ec*RoxA. *Ec*YcaO and *Ec*RoxA co-purify with *Ec*uL16.

**
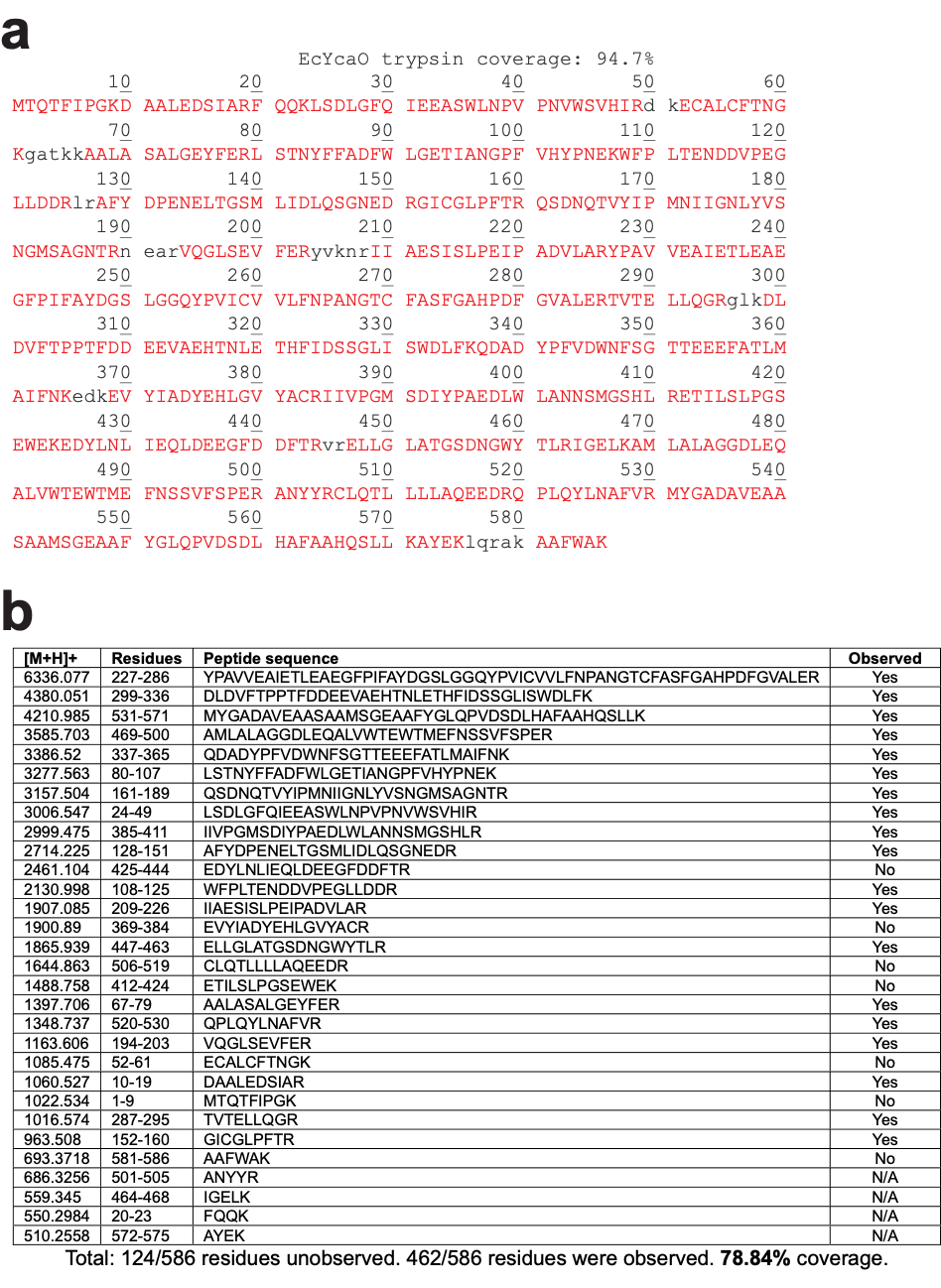
**

**Figure S20.** In-gel trypsin digestion of *Ec*YcaO. *Ec*YcaO co-purified during IMAC-based purification of *Ec*uL16-His_6_. The corresponding SDS-PAGE gel band was excised and trypsinized. (a) sequence of *Ec*YcaO, residues in red are in observable trypsin fragments >500 Da. (b) sequence of each tryptic fragment, along with whether that fragment was observed.

**
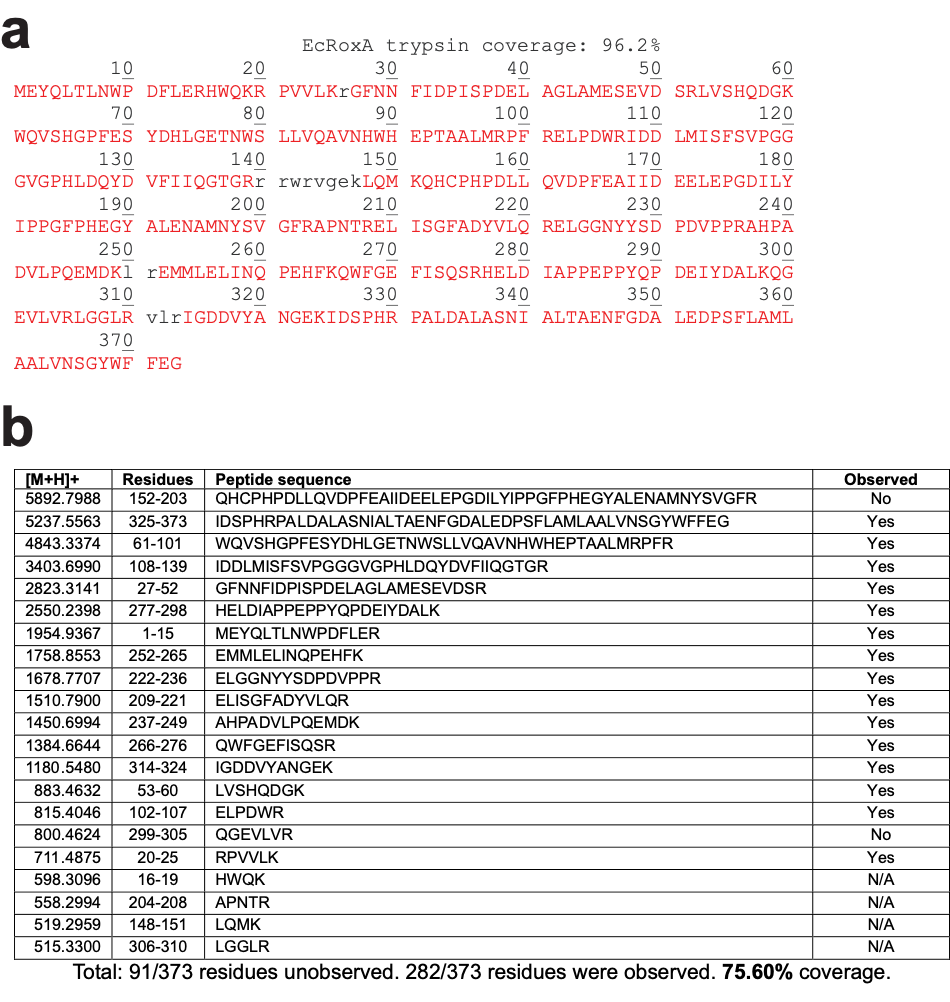
**

**Figure S21.** In-gel trypsin digestion of *Ec*RoxA. *Ec*RoxA co-purified during IMAC-based purification of *Ec*uL16-His_6_. The corresponding SDS-PAGE gel band was excised and trypsinized. (a) sequence of *Ec*RoxA, residues in red are in observable trypsin fragments >500 Da. (b) sequence of each tryptic fragment, along with whether that fragment was observed.

**
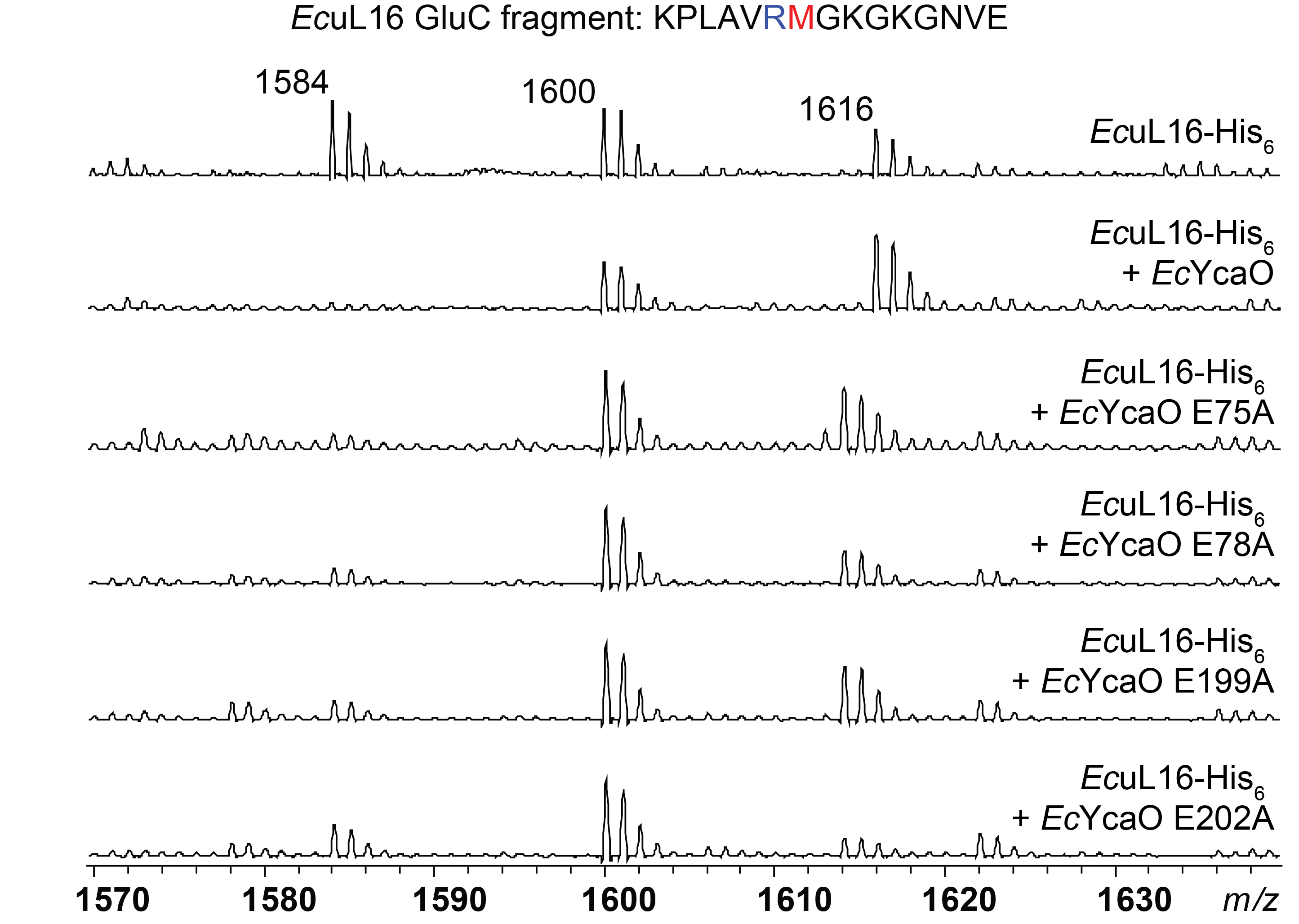
**

**Figure S22.** MALDI-TOF-MS of *Ec*uL16 expressed with ATP-binding deficient *Ec*YcaO. Stacked MALDI-TOF-MS spectra showing the difference in modification status for endoproteinase GluC-digested *Ec*uL16-His_6_ co-expressed with either wild-type *Ec*YcaO or *Ec*YcaO with a mutation that disrupts ATP binding. The expected *m/z* for the unmodified endoproteinase GluC-fragment of *Ec*uL16 is 1584, 1600 for 1 x 16 Da modification (thioamidation or hydroxylation), and 1616 for 2 x 16 Da modifications (thioamidation + hydroxylation). The *m/z* 1614 represents a missed endoproteinase GluC cleavage corresponding to residues 136-150 of *Ec*YcaO, which co-purifies with *Ec*uL16-His_6_.


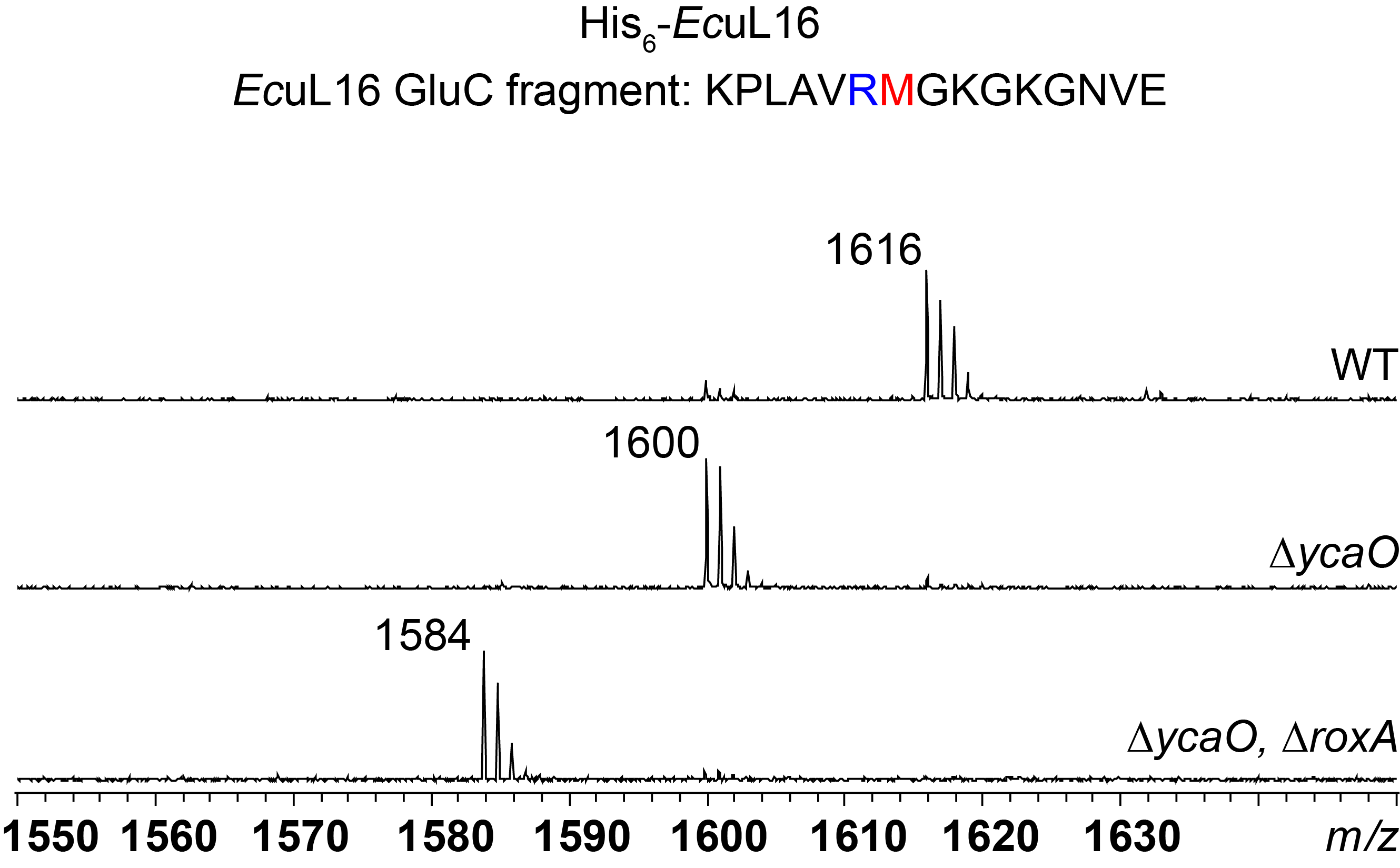


**Figure S23.** MALDI-TOF-MS for *Ec*uL16 expressed in *E. coli* BL21 (DE3) deletion strains. The relevant genotypes for the expression strains are listed on each spectrum. WT = wild-type, BL21 (DE3). The expected *m/z* for the unmodified endoproteinase GluC-fragment of *Ec*uL16 is 1584, 1600 for 1 x 16 Da modification (thioamidation or hydroxylation), and 1616 for 2 x 16 Da modifications (thioamidation + hydroxylation).


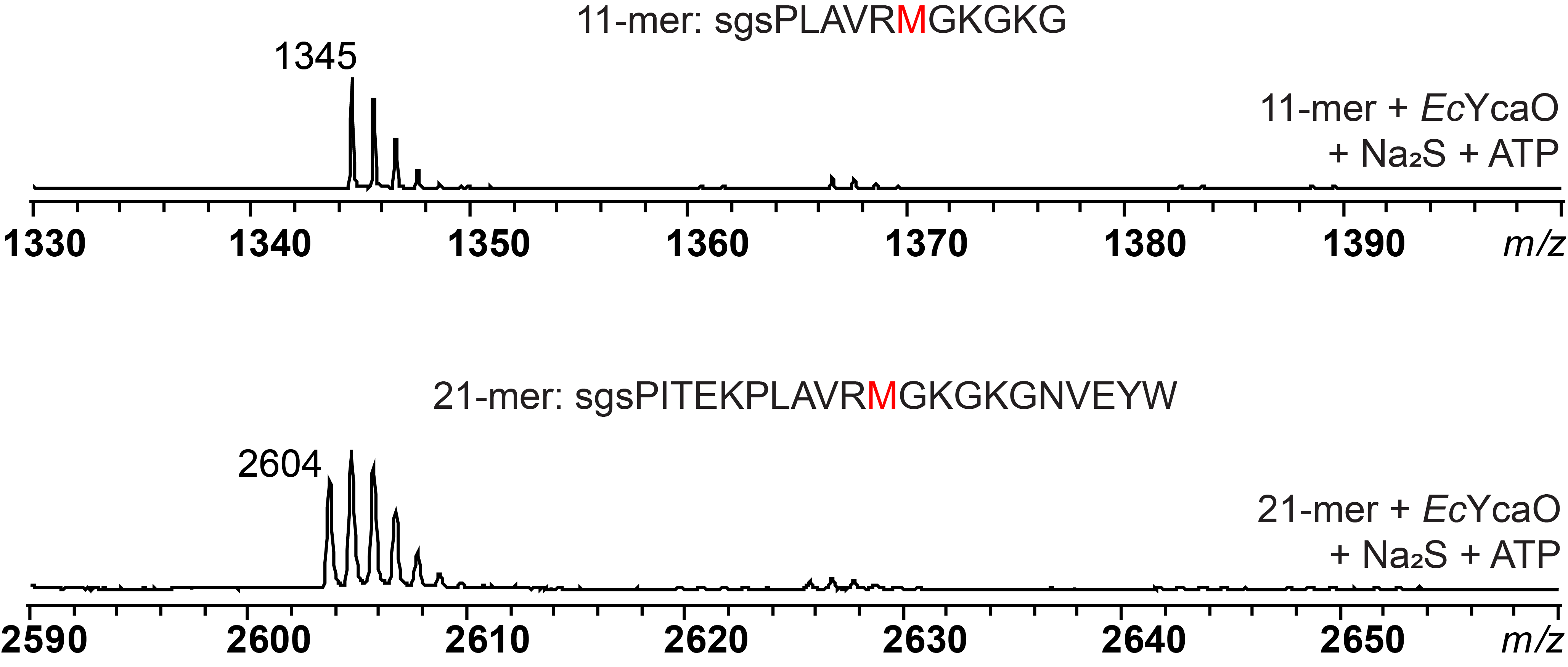


**Figure S24.** MALDI-TOF-MS for *Ec*YcaO reacted with *Ec*uL16 11-mer and 21-mer peptides. The expected m/z values for the 11-mer and 21-mer are 1345 and 2604, respectively. The lowercase “sgs” represents a Ser-Gly-Ser motif scar that remains after TEV protease cleavage.

**
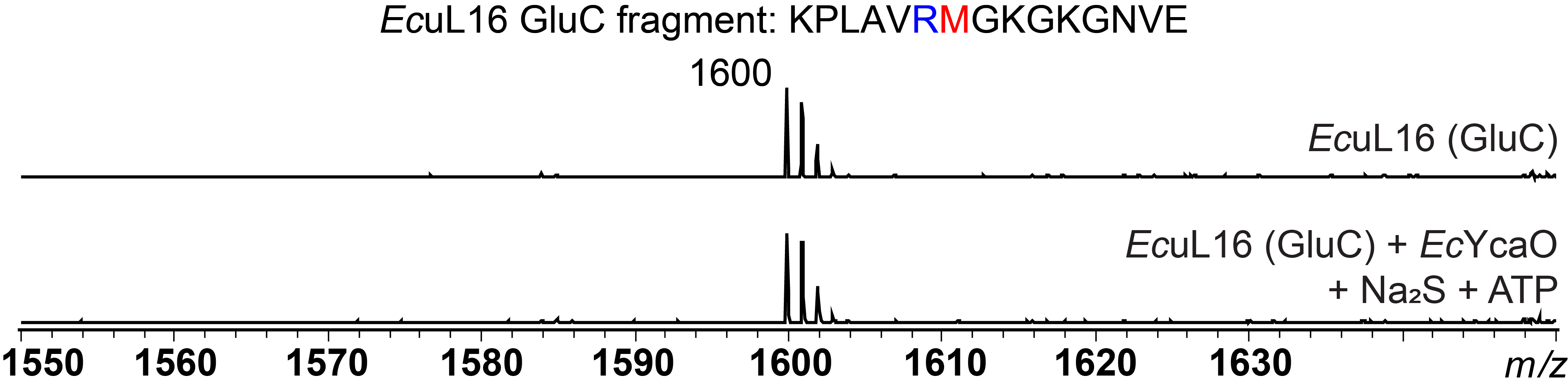
**

**Figure S25.** MALDI-TOF-MS for *Ec*YcaO reacted with endoproteinase GluC-digested *Ec*uL16. The corresponding 1600 Da peptide was not a substrate for *Ec*YcaO *in vitro* under our reaction conditions, as described in the Methods.

**
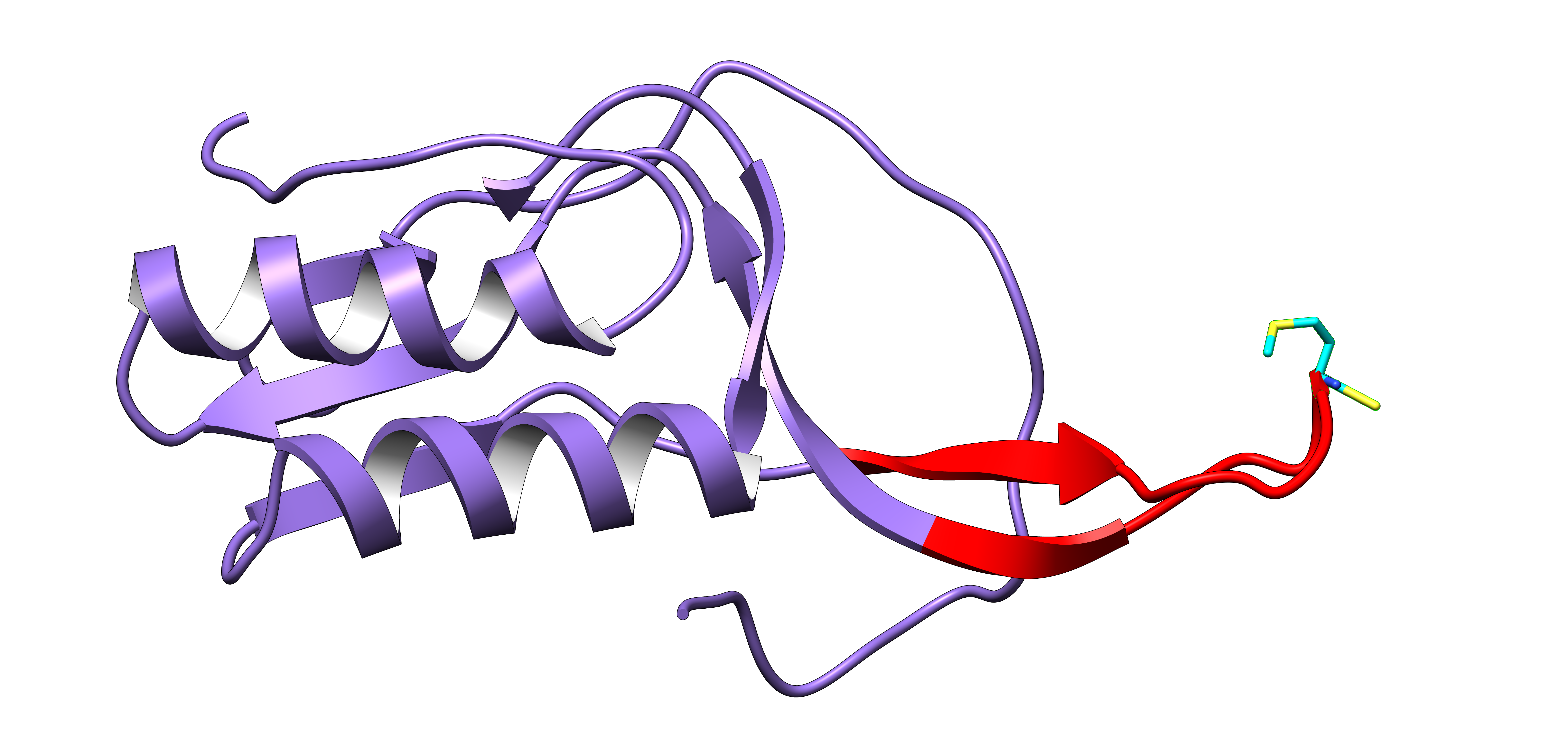
**

**Figure S26.** Structural depiction of the loop region of *Ec*uL16. The structure of *Ec*uL16 was isolated from a cryo-EM structure of the *E. coli* ribosome (PDB: 7K00).^6^ Residues 71-90 are highlighted in red, and the backbone thioamide and side chain of Met82 are displayed in cyan. This figure was generated using Chimera.^5^





**Figure S27.** Competitive FP data for *Ec*uL16 loop region Ala variants. Competitive fluorescence polarization (FP) assays were performed as described in the Methods. His_6_-*Ec*uL16 and variants thereof were used to compete with fluorescein-labeled *Ec*uL16-His_6_-Cys for binding to MBP-*Ec*YcaO.

**Table S5.** Expected fragments of *Ec*uL16 Ala variants when digested with trypsin. These tryptic peptides reveal the modification status of Arg81 (or the equivalent position in R81A), and thus only hydroxylation. Sites of Ala substitution are bolded if they appear in the tryptic peptide.

| ***Ec*uL16 Ala variant** | **tryptic peptide sequence** | **Expected [M+H]^+^** |
| --- | --- | --- |
| WT | VFPDKPITEKPLAVR | 1710.0 |
| P72A | **A**ITEKPLAVR | 1097.7 |
| T74A | VFPDKPI**A**EKPLAVR | 1680.0 |
| L78A | VFPDKPITEKP**A**AVR | 1668.0 |
| R81A | VFPDKPITEKPLAV**A**MGK | 1941.1 |
| M82A | VFPDKPITEKPLAVR | 1710.0 |
| G83A | VFPDKPITEKPLAVR | 1710.0 |
| G85A | VFPDKPITEKPLAVR | 1710.0 |
| V89A | VFPDKPITEKPLAVR | 1710.0 |


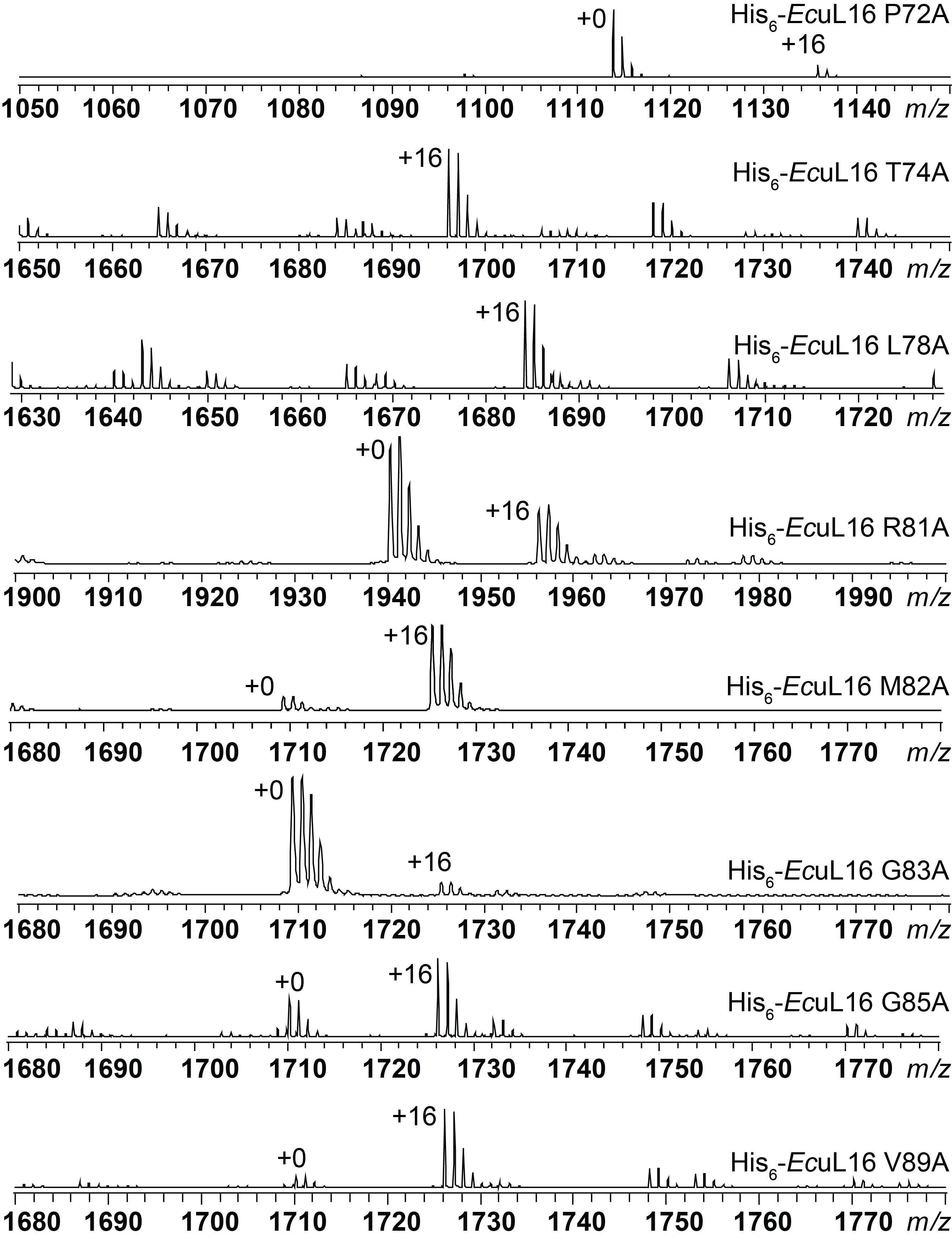


**Figure S28.** Trypsin fragments of modified *Ec*uL16 Ala variants. *Ec*uL16 Ala variants were expressed and purified as described in the Methods.

**Table S6.** Expected fragments of *Ec*uL16 Ala variants when digested with endoproteinase GluC. Both thioamidation and hydroxylation are visible. Sites of Ala substitution are bolded if they appear in the GluC digested peptide.

| ***Ec*uL16 Ala variant** | **GluC peptide sequence** | **Expected [M+H]^+^** |
| --- | --- | --- |
| WT | KPLAVRMGKGKGNVE | 1583.9 |
| P72A | KPLAVRMGKGKGNVE | 1583.9 |
| T74A | KPLAVRMGKGKGNVE | 1583.9 |
| L78A | KP**A**AVRMGKGKGNVE | 1541.9 |
| M82A | KPLAVR**A**GKGKGNVE | 1523.9 |
| G83A | KPLAVRM**A**KGKGNVE | 1597.9 |
| G85A | KPLAVRMGK**A**KGNVE | 1597.9 |
| V89A | KPLAVRMGKGKGN**A**E | 1555.9 |

**
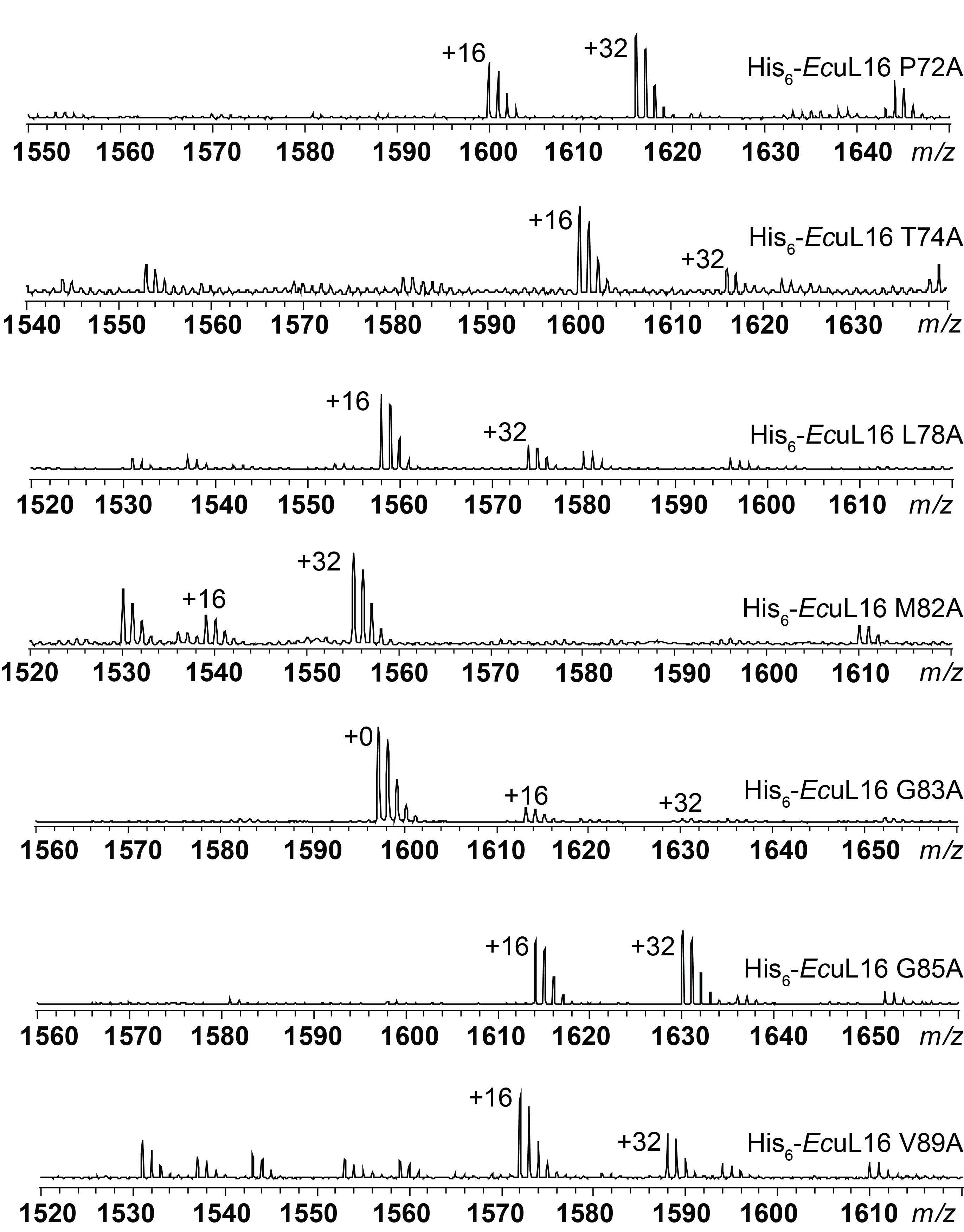
**

**Figure S29.** Endoproteinase GluC fragments of modified *Ec*uL16 Ala variants. *Ec*uL16 Ala variants were expressed and purified as described in the Methods.





**Figure S30.** MBP-*Ec*YcaO Ala variants mapped onto AlphaFold3 structure. *Ec*YcaO is depicted in salmon, and *Ec*uL16 is depicted in purple. The ipTM score for this model is 0.87. Individual residues which were substituted with Ala are depicted in green. This figure was generated using Chimera.^5^








**Figure S31.** FP data for MBP-*Ec*YcaO Ala variants. Fluorescence polarization (FP) assays were performed as described in the Methods. MBP-*Ec*YcaO and variants thereof were used to assess binding with fluorescein-labeled *Ec*uL16-His_6_-Cys.

**

**

**Figure S32.** Isoelectric point comparison of sequence similarity network (SSN) groups. (a) The isoelectric points (pI) of the largest 23 SSN groups are indicated on the SSN. (b) Box and whiskers plot of pI values per group. Group 2, which contains *Ec*YcaO, is red. Group 24 comprises all remaining nodes.


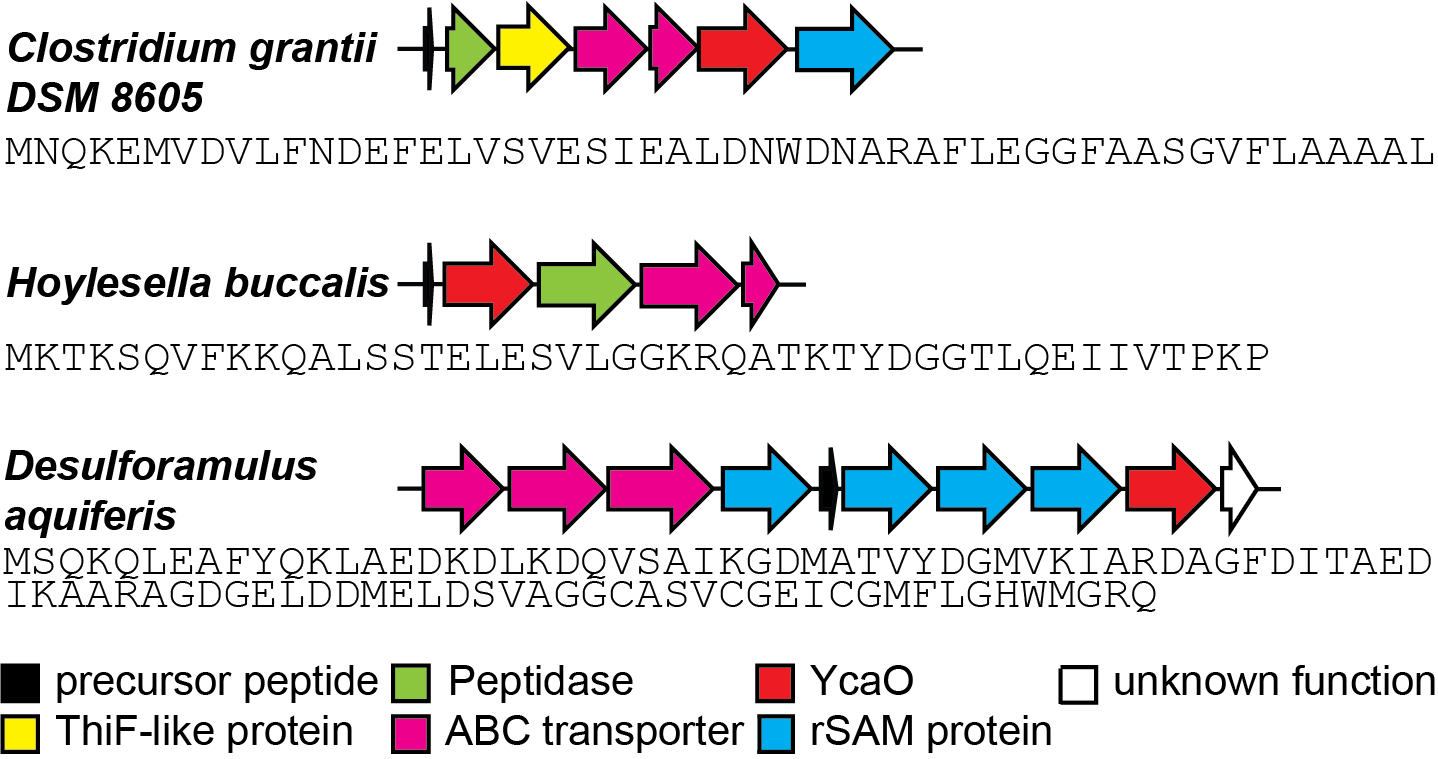


**Figure S33.** Scheme of putative RiPP biosynthetic gene clusters. The sequence of the putative precursor peptide is listed under each ORF diagram.


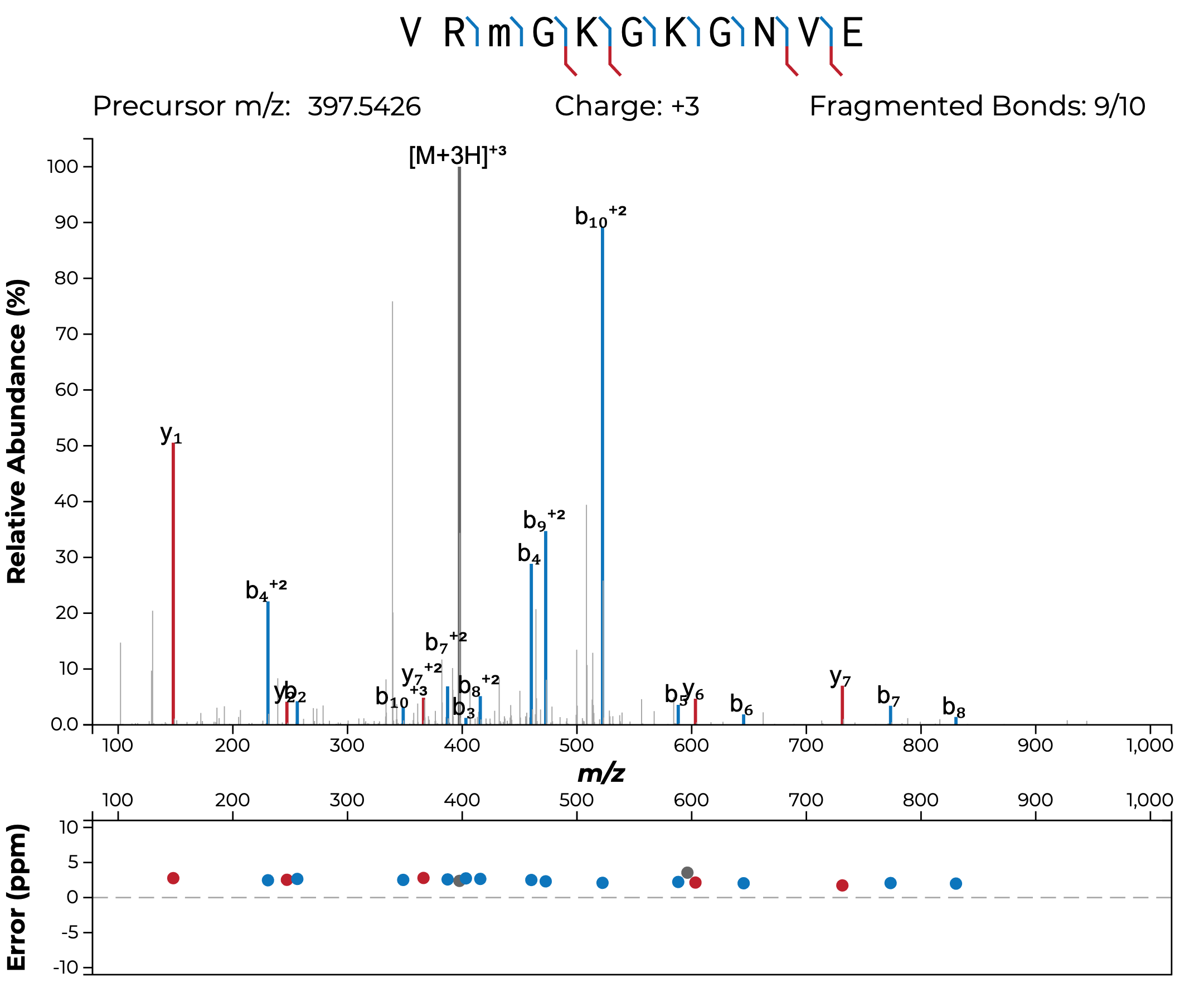


**Figure S34.** HR-MS/MS for thioamidated *Kp*uL16. *Kp*uL16 was co-expressed with *Kp*YcaO, digested with endoproteinase GluC, and analyzed by HR-MS/MS as described in the Methods. The additional +16 Da was confidently localized to the Met residue.


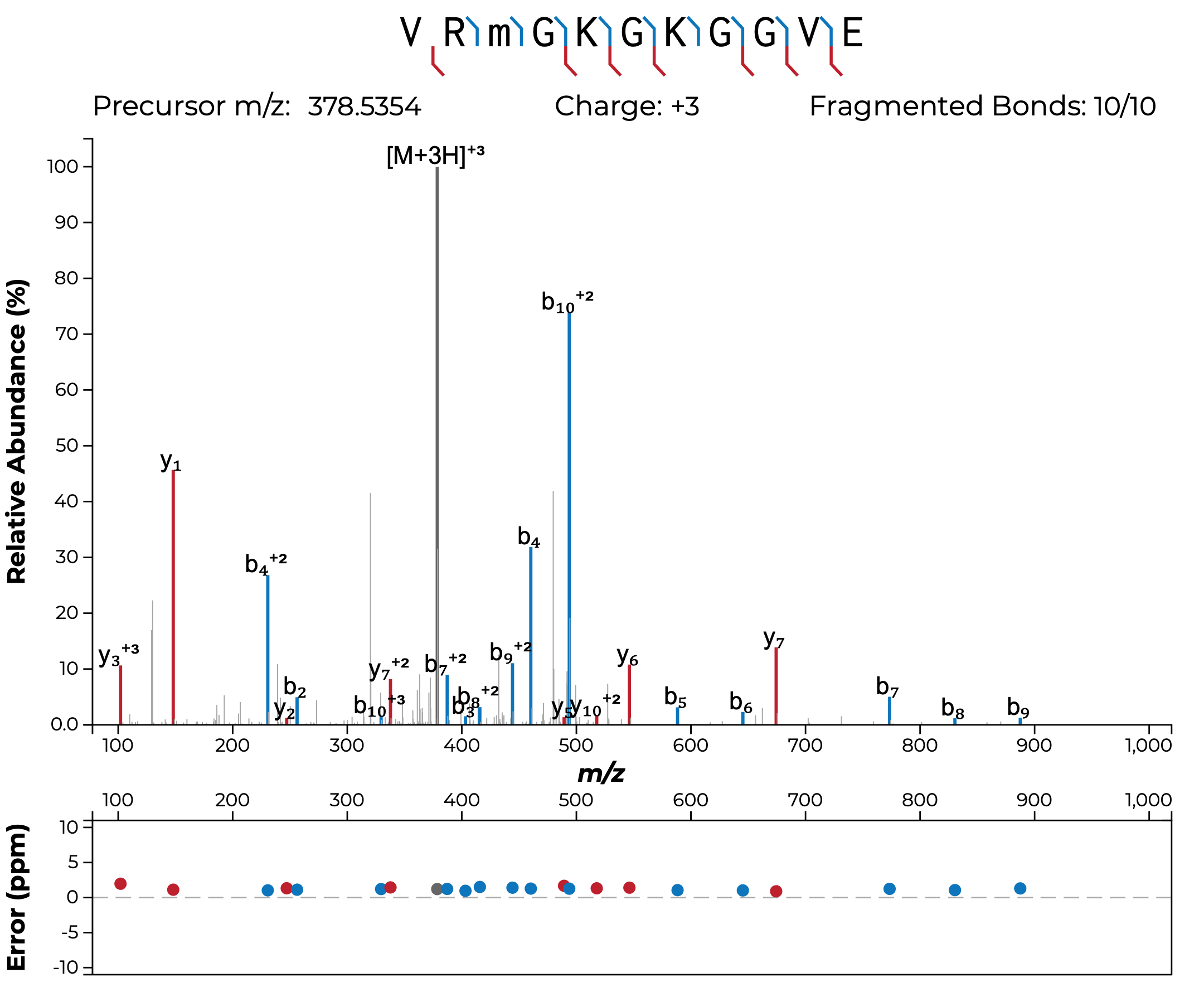


**Figure S35.** HR-MS/MS for thioamidated *Pa*uL16. *Pa*uL16 was co-expressed with *Pa*YcaO, digested with endoproteinase GluC, and analyzed by HR-MS/MS as described in the Methods. The additional +16 Da was confidently localized to the Met residue.

**
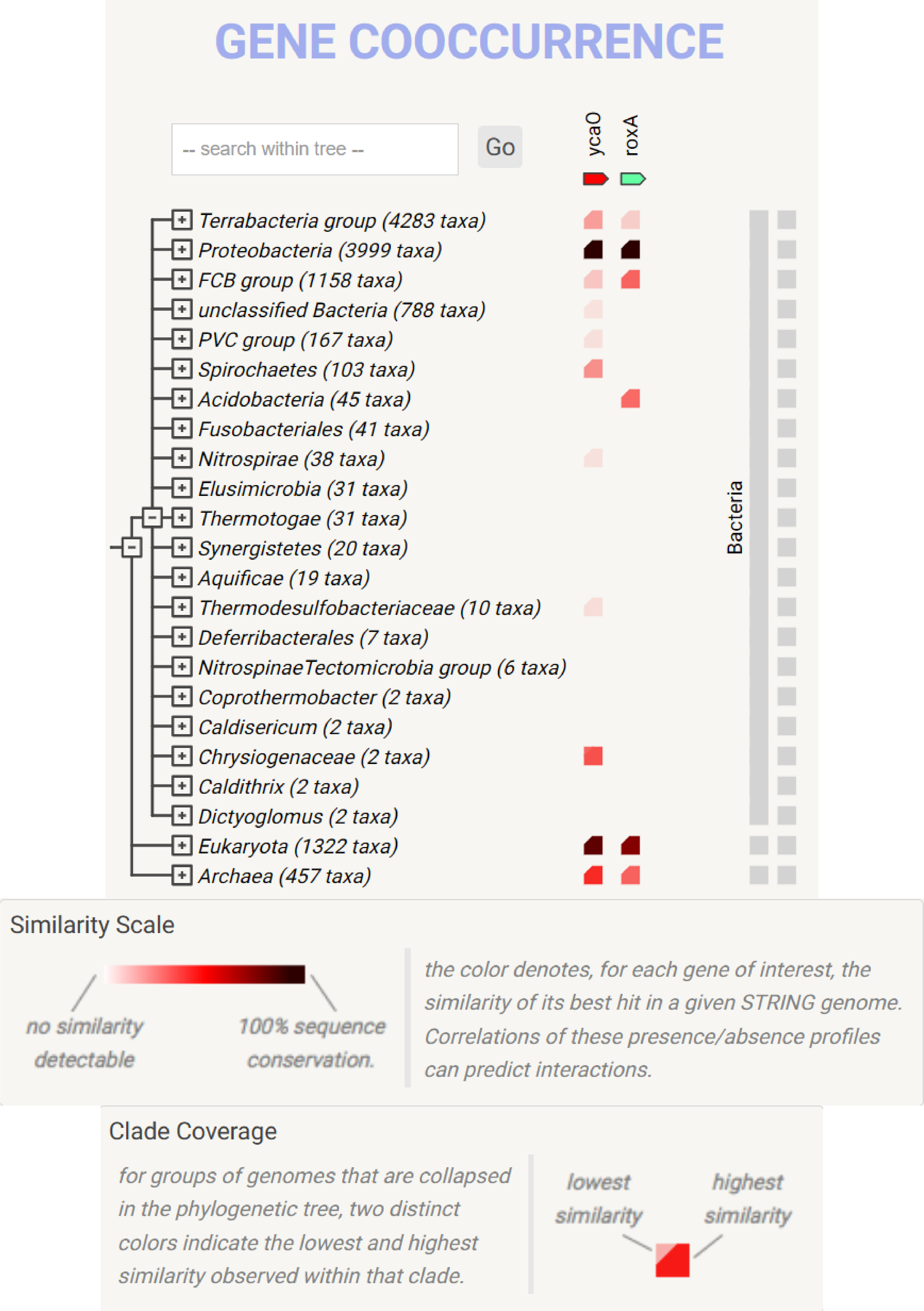
**

**Figure S36.** Genome-wide co-occurrence of *ycaO* and *roxA*. Both proteins are highly co-occurring in Proteobacteria (now reclassified as Pseudomonadota), with minimal sequence derivation. These results were retrieved from STRING.^7^


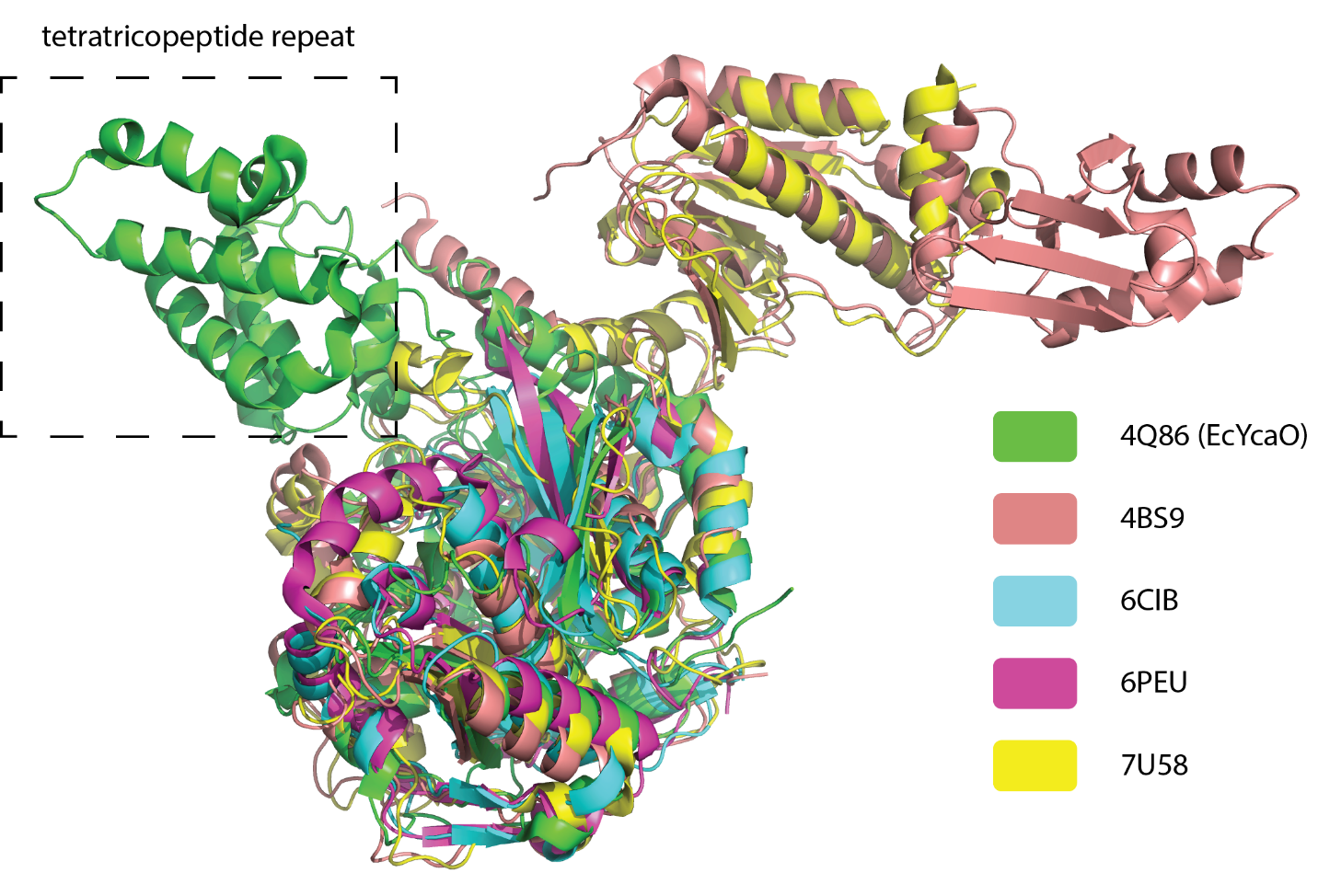


**Figure S37.** Structural alignment of *Ec*YcaO with reported YcaO structures. *Ec*YcaO (PDB: 4Q86), TruD (PDB: 4BS9), *Mk*YcaO (PDB: 6CIB), *Mj*YcaO (PDB: 6PEU), and MusD (PDB: 7U58) were used for structural alignment. The C-terminal tetratricopeptide repeat region of *Ec*YcaO was shown in dotted rectangle. This figure was generated using Pymol.

**
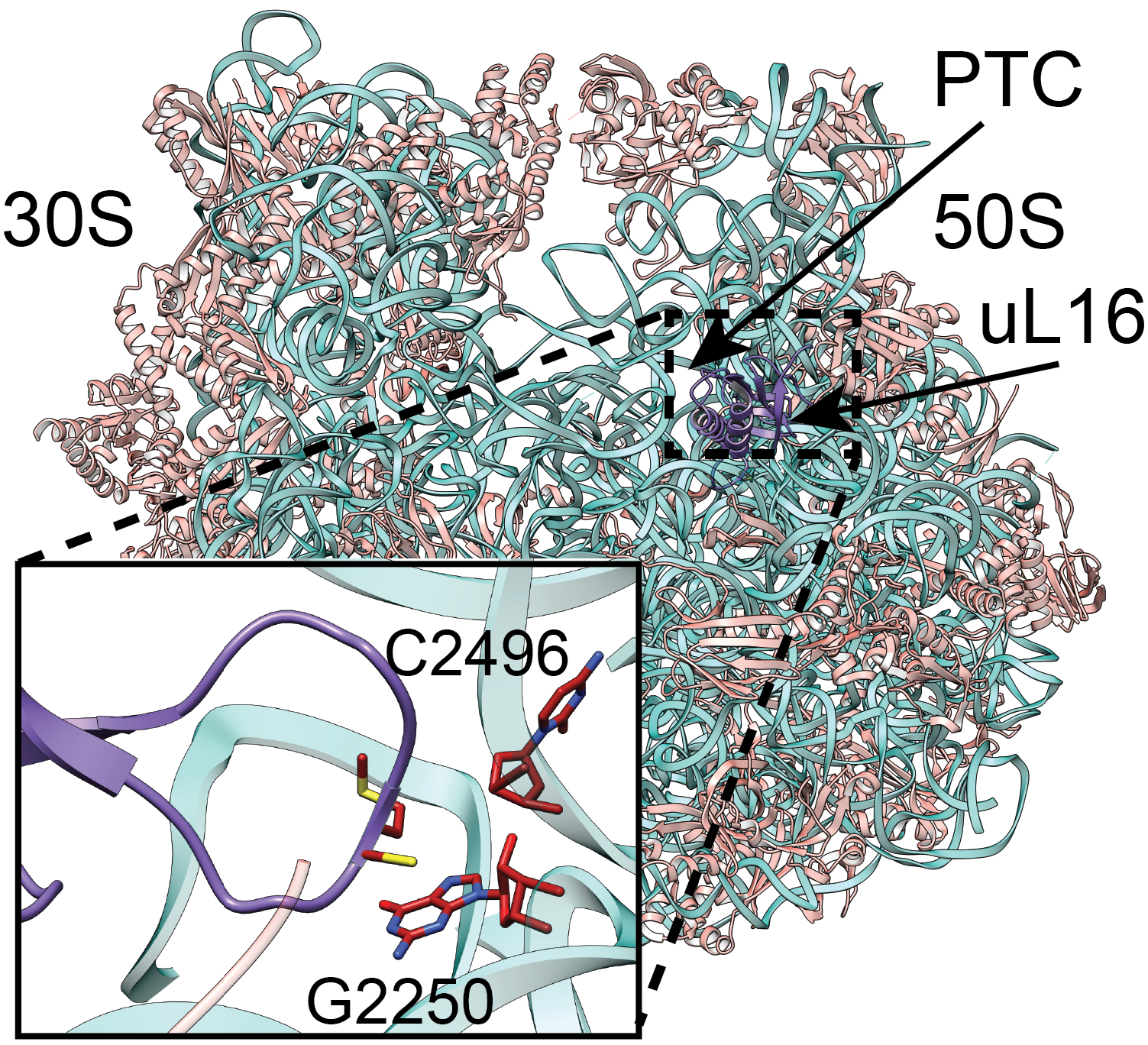
**

**Figure S38.** Met82 of *Ec*uL16 is oriented towards the peptidyl transferase center (PTC). PDB: 7K00.^6^ The inset focuses on the position of Met82 (red stick) of *Ec*uL16 (purple) within the PTC of the ribosome (teal and salmon). The two proximal RNA bases, C2496 and G2250, are implicated in translational speed and fidelity.^8^ This figure was generated using Chimera.^5^

**Supporting References**

(1) Anton, B. P.; Saleh, L.; Benner, J. S.; Raleigh, E. A.; Kasif, S.; Roberts, R. J. RimO, a MiaB-like Enzyme, Methylthiolates the Universally Conserved Asp88 Residue of Ribosomal Protein S12 in Escherichia Coli. *Proc. Natl. Acad. Sci.* **2008**, *105* (6), 1826–1831. https://doi.org/10.1073/pnas.0708608105.

(2) Lee, K.-H.; Saleh, L.; Anton, B. P.; Madinger, C. L.; Benner, J. S.; Iwig, D. F.; Roberts, R. J.; Krebs, C.; Booker, S. J. Characterization of RimO, a New Member of the Methylthiotransferase Subclass of the Radical SAM Superfamily. *Biochemistry* **2009**, *48* (42), 10162–10174. https://doi.org/10.1021/bi900939w.

(3) Landgraf, B. J.; Booker, S. J. Stereochemical Course of the Reaction Catalyzed by RimO, a Radical SAM Methylthiotransferase. *J. Am. Chem. Soc.* **2016**, *138* (9), 2889–2892. https://doi.org/10.1021/jacs.5b11035.

(4) Butland, G.; Peregrín-Alvarez, J. M.; Li, J.; Yang, W.; Yang, X.; Canadien, V.; Starostine, A.; Richards, D.; Beattie, B.; Krogan, N.; Davey, M.; Parkinson, J.; Greenblatt, J.; Emili, A. Interaction Network Containing Conserved and Essential Protein Complexes in Escherichia Coli. *Nature* **2005**, *433* (7025), 531–537. https://doi.org/10.1038/nature03239.

(5) Pettersen, E. F.; Goddard, T. D.; Huang, C. C.; Couch, G. S.; Greenblatt, D. M.; Meng, E. C.; Ferrin, T. E. UCSF Chimera—A Visualization System for Exploratory Research and Analysis. *J. Comput. Chem.* **2004**, *25* (13), 1605–1612. https://doi.org/10.1002/jcc.20084.

(6) Watson, Z. L.; Ward, F. R.; Méheust, R.; Ad, O.; Schepartz, A.; Banfield, J. F.; Cate, J. H. Structure of the Bacterial Ribosome at 2 Å Resolution. *eLife* **2020**, *9*, e60482. https://doi.org/10.7554/eLife.60482.

(7) Szklarczyk, D.; Kirsch, R.; Koutrouli, M.; Nastou, K.; Mehryary, F.; Hachilif, R.; Gable, A. L.; Fang, T.; Doncheva, N. T.; Pyysalo, S.; Bork, P.; Jensen, L. J.; von Mering, C. The STRING Database in 2023: Protein–Protein Association Networks and Functional Enrichment Analyses for Any Sequenced Genome of Interest. *Nucleic Acids Res.* **2023**, *51* (D1), D638–D646. https://doi.org/10.1093/nar/gkac1000.

(8) d’Aquino, A. E.; Azim, T.; Aleksashin, N. A.; Hockenberry, A. J.; Krüger, A.; Jewett, M. C. Mutational Characterization and Mapping of the 70S Ribosome Active Site. *Nucleic Acids Res.* **2020**, *48* (5), 2777–2789. https://doi.org/10.1093/nar/gkaa001.
